# Carbon peak and its mitigation implications for China in the post-pandemic era

**DOI:** 10.1038/s41598-022-07283-4

**Published:** 2022-03-02

**Authors:** Jiandong Chen, Chong Xu, Ming Gao, Ding Li

**Affiliations:** School of Public Administration, Southwestern University of Finance and Economics, Chengdu, China

**Keywords:** Climate sciences, Environmental social sciences, Energy science and technology

## Abstract

China’s carbon peak greatly impacts global climate targets. Limited studies have comprehensively analyzed the influence of the COVID-19 pandemic, changing emission network, and recent carbon intensity (CI) reduction on the carbon peak and the corresponding mitigation implications. Using a unique dataset at different levels, we project China’s CO_2_ emission by 2035 and analyze the time, volume, driver patterns, complex emission network, and policy implications of China’s carbon peak in the post- pandemic era. We develop an ensemble time-series model with machine learning approaches as the projection benchmark, and show that China’s carbon peak will be achieved by 2021–2026 with > 80% probability. Most Chinese cities and counties have not achieved carbon peaks response to the priority-peak policy and the current implementation of CI reduction should thus be strengthened. While there is a "trade off" between the application of carbon emission reduction technology and economic recovery in the post-pandemic era, a close cooperation of interprovincial CO_2_ emission is also warranted.

## Introduction

At the 2015 Paris climate conference, the Chinese government pledged to ensure that CO_2_ emissions peak around 2030. This expression has now been further updated to “strive to reach the peak of carbon emissions by 2030”. This strengthened goal has posed a great challenge, especially in the post-pandemic era. The COVID-19 pandemic has caused the largest reduction in human activities in history, reducing global CO_2_ emissions by approximately 6.4%^1^ in 2020 and 1.7–7.7% in China^2,3^. However, the economy has not fully recovered, and a CO_2_ emission spike may be possible. Therefore, it is too early to conclusively predict that China will reach its carbon peak by the aforementioned period.

China’s carbon emissions—the highest contributor—have been rising since its opening-up policy, but the emission growth rate has slowed in recent years. China’s economy has entered the “new normal” phase, with declining economic growth^4^ and by pursuing high-quality economic development. However, urbanization is still in progress. If the energy system is fundamentally the same (e.g., coal is still the primary source of energy in China), the increase of CO_2_ emissions seems inevitable.

To solve this, the Chinese government has set a series of carbon intensity (CI) targets. According to the Paris Agreement, the CO_2_ emissions per unit of GDP by 2030 shall be reduced to 60–65% below the 2005 levels (the target was recently updated by more than 65%). In 2019, China’s carbon emission intensity decreased by 48.1% compared with that in 2005 (National Bureau of Statistics of China (NBSC)^5^), achieving the reduced CI target of 40–45% by 2020 proposed during the 2009 Copenhagen Climate Change Conference. However, the decline rates of CI in many provinces (e.g., Liaoning and Guangxi) in recent years were significantly lower than those in the last 12th five-year plan period (2011–2015). If the decline rate of the CI continues, it may also affect the realization of China’s carbon peak target.

It is essential to evaluate the peak time and total amount of carbon emission to effectively generate relevant policies and achieve the long-term goal of carbon neutrality by 2060 for China. However, several challenges, such as the data support especially for emission inventory and comprehensive methods, hinder the accurate assessment of the carbon peak. The existing studies on China’s carbon peak exhibit great differences in data, methods, and perspectives, most of which are based on national^6^, provincial^7^, and sectoral data^8–11^, with limited studies on city-level data^12^ wherein the research scope was relatively limited. In terms of methodology, stochastic approaches are widely used such as the regression on population, affluence, and technology (STIRPAT) model^13^, scenario analysis^14,15^, IPAT model^16,17^, environmental Kuznets curve (EKC)^12^, integrated assessment model (IAM)^18^, and forecasting model^19^. In terms of perspectives, studies included energy structural adjustments^20^, industrial restructuring^21^, investment and energy efficiency^22^. Due to these differences, China’s estimated carbon peak period varied from 2020 to 2100 (e.g.,^6,12,22,23^).

In general, existing studies have the following shortcomings: (1) to the best of our knowledge, few studies have comprehensively focused on China’s carbon peak in the post-pandemic period. Although some recent studies attempted to combine carbon peak with the COVID-19 pandemic (e.g.,^24,25^), the carbon peak trajectory of China is inconclusive. Moreover, comprehensive challenges such as the “new normal” economy, the decline in CI and the impact of the COVID-19 pandemic that can affect the carbon peak target, have been largely ignored. (2) Owing to limited data, existing studies rarely consider changes in carbon emissions at every level in the country, which may influence the estimation of China’s carbon peak time and implementation of the important priority-peak policy among cities and counties. (3) The existing research methods are diverse; however, they lack in-depth comparisons that can provide a reference for analyzing carbon peak. (4) China’s carbon peak is seldom quantified on a large scale. Although the Chinese government has recently stressed the priority-peak policy at different levels for achieving a national carbon peak, the areas reaching the peak, especially for cities and counties, are generally unknown. China’s policy implementation follows a hierarchical diffusion process^26^, and it is practically significant to identify whether different levels (i.e., provinces, cities, and counties) have reached the peak. (5) In the post-pandemic era, it is unclear how the spatial pattern of China's sub-national carbon emissions would change for achieving the national carbon peak target. This requires close cooperation in the area of carbon emissions. As the spatial pattern of carbon emissions for regions is very complex, the analysis of complex networks for carbon emission under the dual background of COVID-19 and the carbon peak target, is thus needed.

This study made several contributions to the existing literature.

First, we comprehensively evaluated the time, volume, driver patterns, changing complex network, and policy implications of China’s carbon peak by considering the COVID-19 pandemic and the slowdown of CI reduction, the two factors above being ignored to a large extent. To this end, we developed an ensemble time-series (TS) forecasting model to predict China’s CO_2_ emission trajectories as the benchmark. The forecasting method utilized four machine learning (ML) and eight non-ML approaches and could reduce the prediction deviation caused by irregular data. Unlike most previous studies on carbon peak based on national and provincial datasets, we used a unique dataset on county-level CO_2_ emission data (1997–2019) to analyze China’s carbon peak for the first time. We updated China’s CO_2_ emission datasets in 2018 and 2019 for 30 provinces, 292 cities, and 2735 counties through a top-down framework. We also used the latest official economic growth data to explore scenarios on pathways of carbon peak and improve the timeliness of analysis. The improvements in both data and methodology can assist in providing a comprehensive analysis of China’s carbon peak in the post-pandemic era from multi-perspectives that are largely ignored.

Second, we quantified the status quo of carbon peaks at provincial, city, and county levels for the first time in order to support the priority-peak policy. As discussed above, although China has issued guidelines to reach the peak, determination of the areas at different levels that have already achieved the carbon peak is pending. This study thus can serve as a reference for implementing the priority-peak policy in the country.

Third, the study emphasized the importance of interprovincial closely cooperation in terms of CO_2_ emissions in complex networks for achieving the national carbon peak target. By innovatively introducing social network analysis in the context of carbon peak, we depicted the spatial pattern of interprovincial CO_2_ emission under scenarios adopting different emission reduction technology and economic recovery in the post-pandemic era.

Fourth, we indicated that China would achieve its carbon peak without any exogenous shocks during 2021–2026 at 11.7–13.1 Gt with high probability (> 80%), close to the results of Wang et al.^12^ and Yu et al.^21^ but earlier than that of Fang et al.^7^ and Chen et al.^8^. The estimated peak CO_2_ emission would be 11.7–13.1 Gt, lesser than that of Wang et al.^12^, and larger than that of Mi et al.^18^ and Yu et al.^21^. Gaps in China’s CO_2_ emissions between the business-as-usual (BAU) and the advanced emission reduction technology scenarios could be 8.4 Gt in 2030 and 13.4 Gt in 2035. However, the status quo of carbon peak remains undesirable as most provinces, cities, and counties in China have not achieved the carbon peak by 2019. The driver patterns of CO_2_ emission (e.g., CI), have changed in the post-Kyoto era for both Chinese provinces and cities categorized by population size and economic structure. Therefore, the current implementation of CI reduction should be strengthened through emission reduction technology innovation to reach the carbon peak by 2030. The realization of carbon peak target also requires cooperation of interprovincial CO_2_ emission while there is a "trade off" between application of carbon emission reduction technology and economic recovery in the post-pandemic era. Our study will provide new insights to assist policy implementations.

## Results and discussion

### Decomposition for provinces and city groups

**Figure 1:**
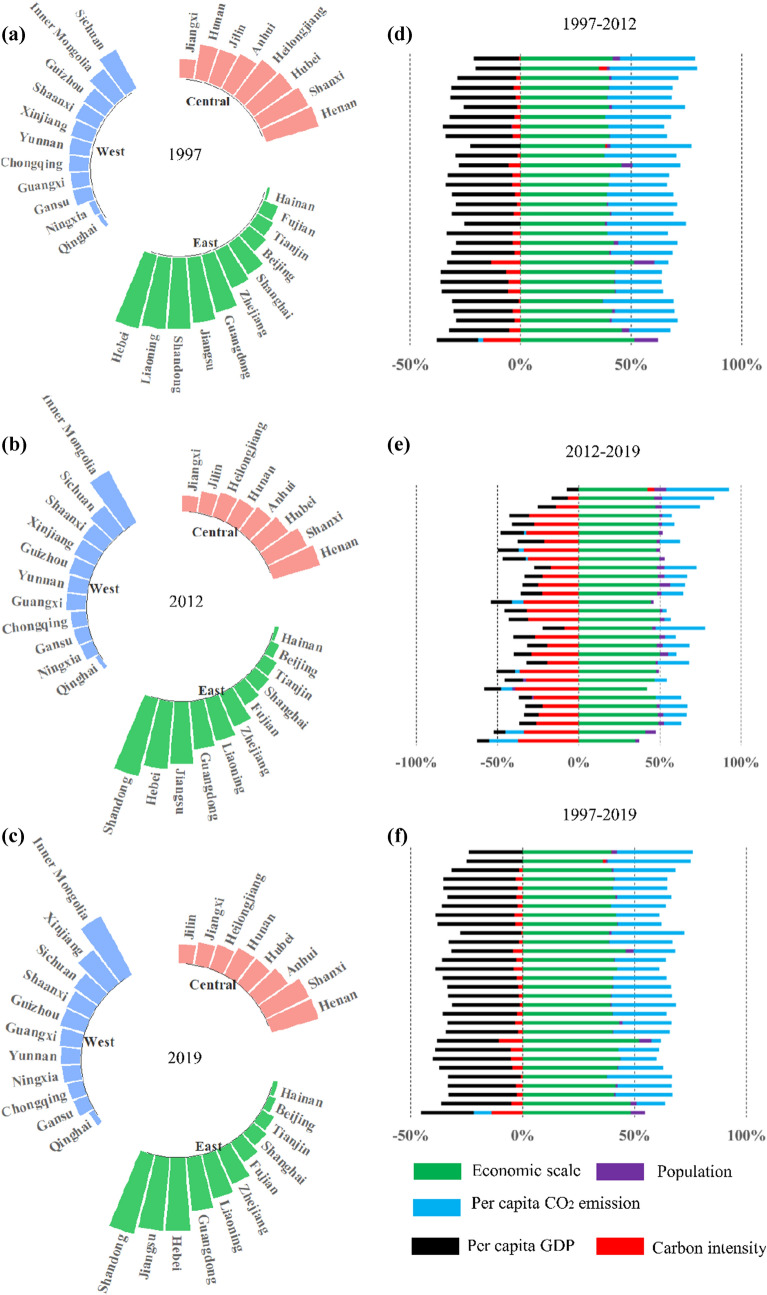
Changes in CO_2_ emissions of Chinese provinces and its five drivers in 1997–2019. (**a**–**c**) Rank changes in aggregate CO_2_ emission among the east, central, and west regions in 1997, 2012, and 2019. The bars from top to bottom for (**d**–**f**) represent Xinjiang, Ningxia, Qinghai, Gansu, Shaanxi, Yunnan, Guizhou, Sichuan, Chongqing, Hainan, Guangxi, Guangdong, Hunan, Henan, Shandong, Jiangxi, Fujian, Anhui, Zhejiang, Jiangsu, Shanghai, Heilongjiang, Jilin, Liaoning, Inner Mongolia, Shanxi, Hebei, Tianjin, and Beijing.

The distributions of the five drivers in 1997–2019 were similar to those in 1997–2012. In addition, the driver patterns of CO_2_ emission changed in the post-Kyoto era especially the role of reducing CI. The changes in ranks in terms of CO_2_ emissions among Chinese provinces were smaller in the post-Kyoto era than in the Kyoto era, especially in China’s central and eastern regions shown in Fig. 1b,c. The relative ranks in the west still changed in the post-Kyoto era, except the Inner Mongolia and Qinghai, which were mainly due to the abundant coal resources and low economic development^27^. In general, the relative ranks changed dramatically for most provinces in the Kyoto era (Fig. 1a,b). For example, Shandong became the greatest emitter among 30 Chinese provinces after the Kyoto era. These results implied that the realization China’s climate targets would rely on the CO_2_ emission performance of the western region. A tradeoff between CO_2_ emission performance and economic development must be considered as the west is the least economically developed among the three regions.

The GDP per capita (PY) and CI were inversely correlated to CO_2_ emission from 1997 to 2012 (Fig. 1d) and the whole study period (Fig. 1f) in provinces at varying levels. Following the environmental Kuznets curve^28,29^, the results were unsurprising since the PY in China increased since the opening-up policy, and the Chinese government’s pledge in 2009 to reduce the CI by 40–45% in 2020^30^ earned significant results, including those since the post-Kyoto era (Fig. 1e).

Conversely, the GDP (Y), population (P), and CO_2_ emission per capita (PC) in provinces were positively correlated to CO_2_ emissions in 1997–2012, in which Y and PC were the main drivers and generally consistent with Zhang et al.^22^. The Y became the dominant driver increasing the CO_2_ emission in the post-Kyoto era while the P had relatively minimal contribution due to the slow population growth. The inflow and outflow of population played a limited role in declining CO_2_ emission due to its mobility^31^. According to the World Bank^32^, China’s PC in 2011 was 7.242 metric tons per capita, which overtook that of the European Union (7.081 metric tons per capita) for the first time. With the rapid industrialization and urbanization, Y was growing at a high speed of 9.8% (NBSC), thus resulting in high CO_2_ emissions from coal that dominated China’s energy consumption structure^4^.

**Figure 2:**
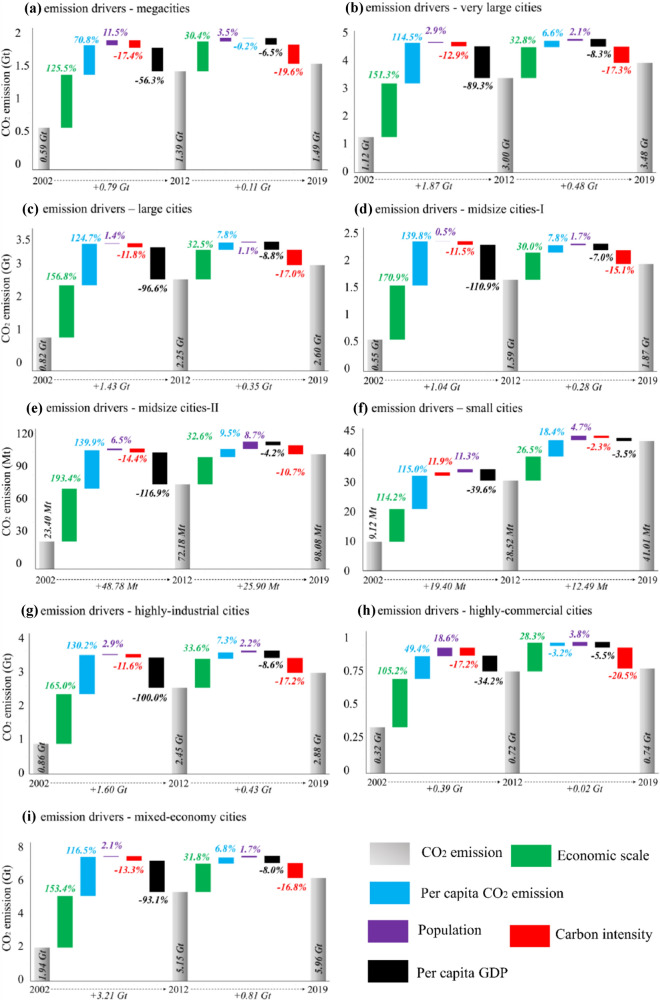
Drivers of changes in CO_2_ emissions among Chinese cities categorized by population size and economic structure in 2002–2019.

Among the six city groups, large cities and very large cities were the two greatest CO_2_ emitters, occupying 68.8% of the national population in 2015. These two city groups were followed by midsize cities-I and megacities. As shown in Fig. 2a–f, Y, PC, and P were the three greatest positive drivers of CO_2_ emissions from 1997 to 2012 in all city groups except in small cities, which were the same throughout the period except in megacities which may be caused by the slow population growth. Figure 2g–i showed that compared to highly commercial and mixed-economy cities, highly industrial cities’ CI contributed a decrease of CO_2_ emission from 11.6 to 17.3% in the post-Kyoto era.

The CO_2_ emissions linked to PY and CI were declining in all city groups, similar to provinces in 1997–2012. The strengthened implementation of CI reduction in the country made this possible. However, we found that the contributions of CI and PY increased with city types, i.e., from small cities to megacities. Therefore, assuming that the population and urbanization continue to expand, the CO_2_ emissions may increase (e.g., changing from midsize cities-II cities to midsize cities I). Hence, the role of CI reduction measures among cities becomes essential as the level of urbanization increases^33^.

### Carbon peaks in China

**Figure 3:**
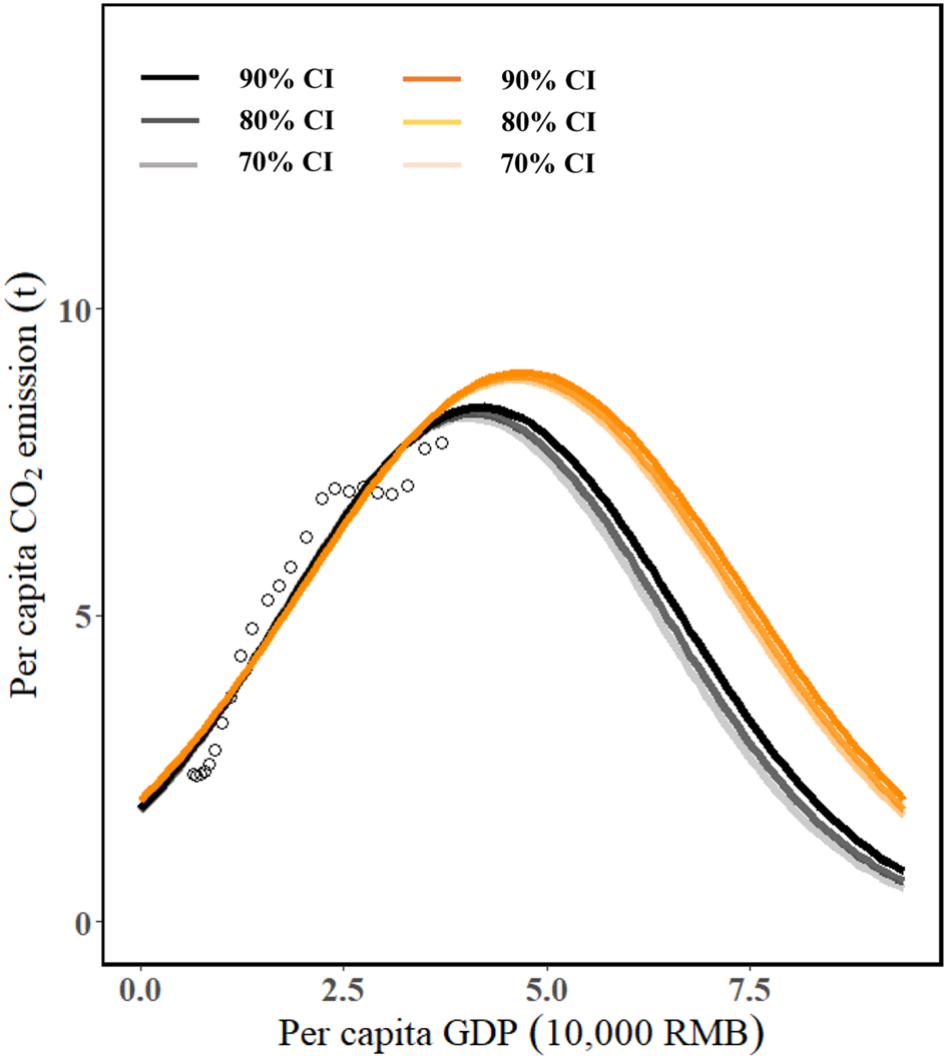
Relationship between annual GDP per capita and CO_2_ emissions per capita from China. The curves denote the GDP per capita and their corresponding CO_2_ emission per capita of Chinese provinces (black) and cities (orange) at 70%, 80%, and 90% confidence levels; the GDP per capita is based on the constant prices in 1997, and CO_2_ emission per capita in 1997–2019 is updated in the study.

Figure 3 depicted the relationship between PC and PY of provinces and cities. We applied a GKC (Eq. 11) to fit PY and PC for Chinese provinces and cities and then calculated the mean value together of $p{y}_{peak}$ with the confidence intervals 70%, 80%, and 90%, respectively, as described in methodology section. Therefore, we can calculate the national $p{y}_{peak}$ with different confidence intervals since we assumed that the national PC and PY would be constant for most provinces and cities, as also applied by Wang et al.^12^. We found that peak PCs were 8.3–9.3 ton/person. Based on the projections of China’s population and economic growth, we projected that China would achieve carbon peak between 2021 and 2026 with > 80% probability, close to the results of Wang et al.^12^ and Yu et al.^21^ but earlier than that of Fang et al.^7^ and Chen et al.^8^. The estimated peak CO_2_ emission would be 11.7–13.1 Gt, lesser than that of Wang et al.^12^, and larger than that of Mi et al.^18^ and Yu et al.^21^.

**Figure 4:**
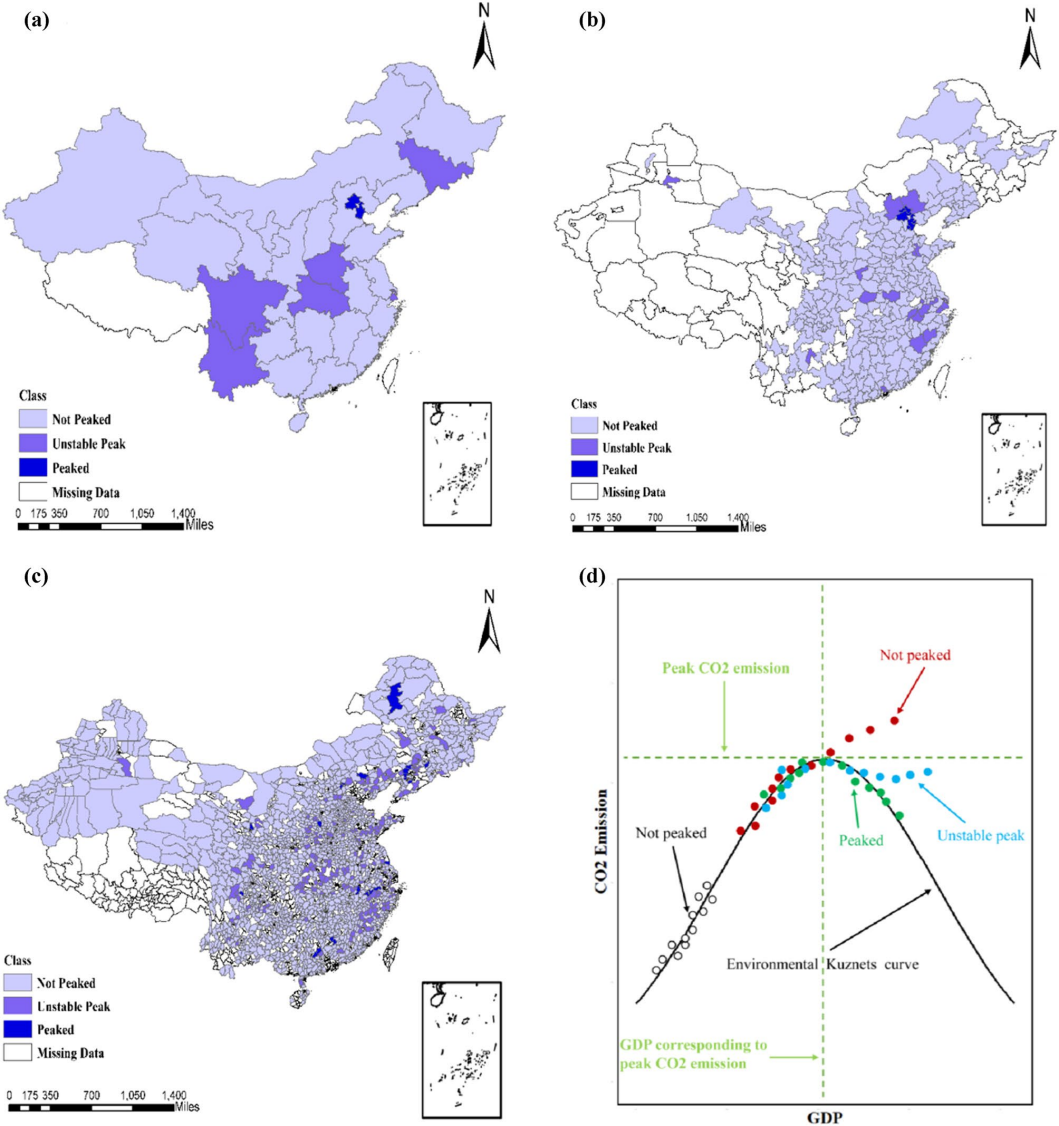
Carbon emission levels in Chinese provinces (**a**), cities (**b**) and counties (**c**). (**a**,**b**) are based on the observations in 2019, while (**c**) pertains to 2018 due to missing data. (**d**) Illustrates the positions of carbon emission levels in the GKC. (**a**–**c**) were produced by ArcGIS (10.2 https://support.esri.com/en/products/desktop/arcgis-desktop/arcmap/10-2-2#downloads). Figure (**d**) was generated by R software (3.6.1, https://cran.r-project.org/bin/windows/base/old/3.6.1/).

Figure 4 depicted the carbon emission levels in Chinese provinces, cities, and counties. The results showed that only Beijing and Tianjin cities achieved carbon peaks. A total of 21 cities had unstable carbon peaks while 239 cities did not achieve carbon peaks. Jilin, Shanghai, Henan, Hubei, Sichuan, and Yunnan provinces had unstable carbon peaks in 2019, and more than two-thirds of the provinces have not reached their carbon peaks. At the county level 22 counties achieved carbon peaks, 184 counties showed unstable carbon peaks, and 1526 counties did not achieve their carbon peaks. Policymakers can monitor and update the CO_2_ emission levels shown in Fig. 4 to guide in implementing priority-peak policies at local levels.

### Scenario analysis

**Figure 5:**
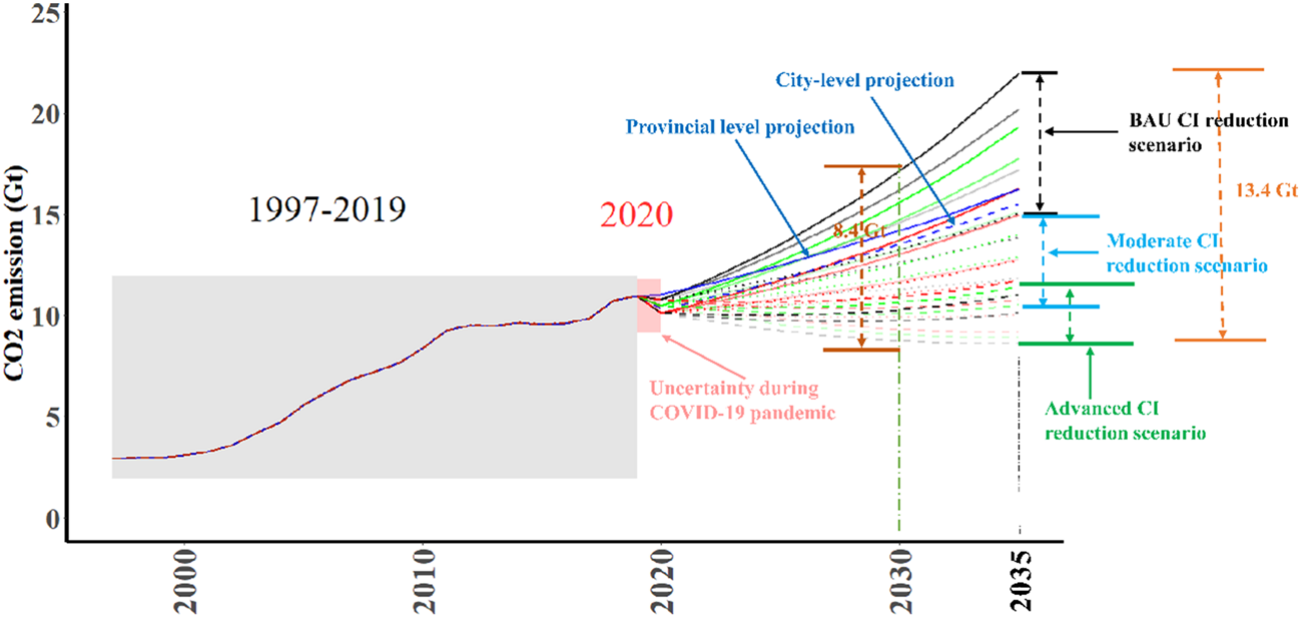
Projections of China’s CO_2_ emissions by 2035 under different scenarios.

Figure 5 depicts the trajectories of China’s CO_2_ emission under different scenarios by 2035 and illustrates the trajectories of China’s CO_2_ emission based on the ensemble TS forecasting model at provincial and city levels. China’s overall CO_2_ emissions decreased by approximately 0.18 Gt–0.84 Gt due to the COVID-19 pandemic.

We further showed that if China would follow the advanced scenario, 2020 could be the year of the county’s carbon peak. However, if China would implement the moderate scenario, it could achieve the carbon peak by 2030, depending on the implementation of CI reduction and economic growth. The future implementation of CI reduction in the past decade (2011–2020) may also contribute to a carbon peak in the country. If the rate of CI reduction from 13th FYP period is continued, China will not achieve carbon peak by 2030. In fact, compared with the CI reduction during 12th FYP period, China recently slowed its CI reduction efforts at provincial levels, according to a report by the government^34^. Therefore, strengthening the implementation of CI reduction in the future especially for the 14th FYP (2021–2025) is key for achieving national carbon peak.

Gaps of China’s CO_2_ emission under three scenarios could be 8.4 Gt in 2030 and 13.4 Gt in 2035. However, due to uncertain CI reduction and economic growth, the future trajectory of CO_2_ emission is likely to deviate from the assumed scenarios. Combined with the results of nonlinear estimation using GKC, the scenario analysis indicates that the uncertainty in the achievement of carbon peak by 2030 is primarily due to the pandemic and slowdown in CI reduction. However, we are optimistic that China will achieve its carbon peak target if the implementation of CI reduction is strengthened.

### Social network analysis

**Figure 6:**
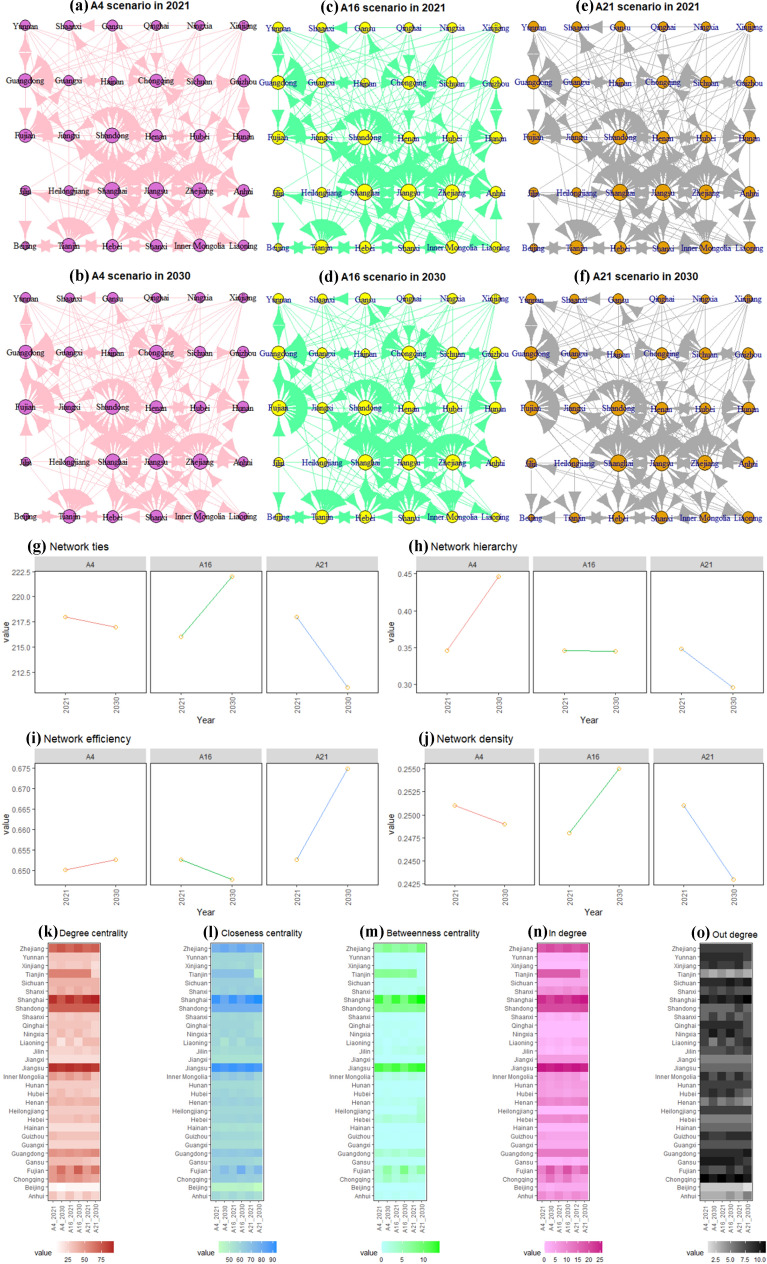
Results of social network analysis on provincial CO_2_ emissions in 2021 and 2030 in China under different scenarios.

To illustrate the changes of regional carbon emission spatial correlation network in China at the sub-national scale (provincial scale in the study) under different emission reduction technology scenarios in the post-pandemic era, we used three representative scenarios to conduct the social network analysis (SNA, the supplementary material S1). Figure 6a–f depicts complex networks of China’s CO_2_ emissions at provincial level under different scenarios in the post-pandemic era, while Fig. 6g–j, k–o show the overall and individual characteristics of provincial networks of CO_2_ emissions. Whether it is BAU or moderate or advanced emission reduction technology scenarios, the interprovincial carbon emission spatial correlation network presents a complex network structure. Due to the different scenarios of economic recovery in the post-pandemic era and emission reduction technology, the corresponding network characteristics show great differences.

The number of interprovincial carbon emission spatial correlation networks decreased, with different reasons as those under A4 and A21 scenarios. A4 shows that although the carbon emission reduction technology maintains the status quo, the rapid economic growth could widen the original economic gap among provinces and slightly impact carbon emission spatial network ties. Compared with other emission reduction technology scenarios, the use of advanced carbon emission reduction technology could increase the socioeconomic costs and impact the carbon emission spatial network ties. A16 scenario shows that moderate improvement of carbon emission reduction technology will improve the spatial correlation network of carbon emission, implying that there is a "trade off" between application of carbon emission reduction technology and socioeconomic cost of economic recovery.

The other characteristics of overall network also reflect the above patterns. For example, Fig. 6I shows that the overall network efficiency increases under A4 and A21 scenarios but decreases under A16 scenarios. This indicates that moderate carbon emission reduction technology is conducive to improving the connection number of interprovincial carbon emission spatial correlation network and enhancing the network stability. The higher the network density is, the closer the interprovincial carbon emission spatial correlation network is. The changing trend of network density under different scenarios is similar to that of Fig. 6g, which also reflects “trade off.” The results of network hierarchy analysis are slightly different. Figure 6h shows that the network hierarchy of A16 and A21 scenarios has declined except for the A4 scenario, indicating that maintaining the existing carbon emission reduction technology is not conducive to breaking the strict spatial correlation structure of carbon emissions. In contrast, by improving technological progress in carbon emission reduction, the strict spatial correlation structure of interprovincial other emissions can be further broken, whereas the interprovincial network interaction can be enhanced.

In terms of characteristics of individual network, the results of in degree and out degree show that the in degree of Tianjin, Hebei, Shanghai, Zhejiang, Fujian, Shandong, Henan, Guangdong and other provinces is not only higher than the national average in degree, but also higher than their own out degree under the three scenarios (Fig. 6n–o). Most of them are located in the central and eastern regions with developed economy and high carbon emissions, and they are highly dependent on the energy supply from other provinces, which may lead to carbon emission spillover from other provinces. The analysis on degree centrality is similar to those in degree and out degree results (Fig. 6k). In all scenarios, Tianjin, Shanxi, Inner Mongolia, Shanghai, Jiangsu, Zhejiang, Fujian, Shandong, Guangdong and other provinces with higher degree centrality than the national average are located in coastal areas except Inner Mongolia, suggesting that coastal areas have a strong impact on the spatial correlation network and spatial spillover effect of carbon emissions under different scenarios.

The results of betweenness centrality analysis show that Tianjin, Shanghai, Zhejiang, Fujian, Shandong, Guangdong and other provinces have a strong ability to influence the carbon emissions interaction among other provinces in the network (Fig. 6m). The closeness centrality of the above major provinces is also higher than the average value of the national closeness centrality (Fig. 6l), suggesting that these provinces can connect with other provinces faster in the carbon emission spatial network, that is, they play a central role in the network.

Although the above analysis highlights the common mode of interprovincial carbon emission spatial correlation network under different scenarios, future relevant policies should consider provinces with different performance including the strength of connections in terms of carbon emission network under different scenarios (Fig. 6a–f). Regional carbon emission reduction policies should fully consider Shandong, Guangdong, Tianjin, Zhejiang and other provinces with relative strong connections of spatial emission networks, and can be also conducted by guiding provinces with more energy transmission such as Inner Mongolia, Shanxi and Guizhou without harming economic growth, so as to affect the emission pattern of the eastern and central regions, realize the provincial carbon emission regulation under the national carbon peak target and promote China's carbon emissions into a stable downward path.

## Concluding remarks

For Chinese government’s goal of achieving carbon neutrality, achieving carbon peak before 2030 is imperative. The pandemic and slowdown in declining CI cannot be ignored. Based on the new and a large-scale dataset of China's CO_2_ emissions at provincial, city, and county levels, we developed several methods to analyze the time, volume, driver pattern, emission network, and policy implications of China’s carbon peak. For the first time, this study identified peak areas up to the county levels, providing an important reference in formulating priority-peak policies. The study emphasized the importance of interprovincial closely cooperation of CO_2_ emission in complex networks toward the national carbon peak and carbon neutrality targets.

This study showed that China would achieve its carbon peak without any exogenous shocks in 2021–2026 at 11.7–13.1 Gt with a high probability of > 80%. However, due to the COVID-19 pandemic and slowing rate of CI reduction, the achievement of the carbon peak by 2030 remains uncertain. Under scenarios between the BAU and the advanced emission reduction technology, gaps in China’s CO_2_ emissions could be 8.4 Gt in 2030 and 13.4 Gt in 2035. Further, the generalized Divisia index analysis indicated that CI reduction is more important for reducing CO_2_ emission in Chinese provinces and cities categorized by population size and economic structure in the post-Kyoto era. Therefore, the current implementation of CI reduction should be strengthened through emission reduction technology innovation to assist in the achievement of the carbon peak by 2030 and leading emissions into a stable downward path for achieving the carbon neutrality target by 2060. Since most provinces, cities and counties in China have not achieved their carbon peaks by 2019, a necessary condition for achieving the national targets above is to formulate close cooperation in terms of interprovincial CO_2_ emissions. However, the SNA showed that there is a "trade off" between application of carbon emission reduction technology and economic recovery in the post-pandemic era.

In this regard, we recommend the following policies. First, implementing green economy recovery after the coronavirus pandemic, increasing the scale of green investment, and balancing economic growth and emission reduction targets. Although China has the second-largest green investment scale in the world^35^, the current policies may be insufficient to achieve China's carbon peak and other climate goals. The investment in hydrogen energy, carbon capture and storage (CCS), energy storage, electric transport, electric heat, and renewable energy should be further increased in the future. Additionally, given that green finance is an important investment, the government should also standardize its green bond issuance as soon as possible, and the relevant standard system should be in line with the international standards, similar to those in Europe. In line with this, China can strengthen cooperation with the European Union and other regions to improve the scale and quality of green bonds and the contribution of China's green proposal to the global climate target below 1.5–2 °C.

Second, the Chinese government can establish a rapid response system of regional carbon peaks to implement a guideline for prioritizing various areas. Real-time monitoring is difficult when an area reaches the carbon peak. Therefore, increasing the timeliness of updating CO_2_ emission data with a unified carbon inventory accounting system based on the top-down and bottom-up methods is essential. Additionally, given the drivers important role in changing CO_2_ emissions, policy makers can also consider using different methods (e.g., generalized Divisia index method, GDIM) to track and project the trend of the regional CO_2_ emissions and carbon peak. Furthermore, when formulating regional peak strategies, policymakers should fully consider carbon sequestration based on vegetation and the differences of vertical management structure of regional carbon peak plans. For example, policymakers can formulate the relevant carbon peak policies from the differences of key industries and urbanization process in provinces, cities and counties.

Third, the government should manage the regional CI targets through dynamic optimization especially in the 14th FYP, a key period for achieving its carbon goals in the long term. The local governments, in particular, should ensure timely update of the CI reduction for dynamic management of the targets. If we maintain the CI’s decline rate as that during the 13th FYP (2016–2020), CO_2_ emissions may spike in the future. It is, therefore, necessary for the government to focus on CI reduction; however, considering the urgency of economic recovery, this may be difficult. Policymakers should also formulate more detailed regional emission reduction cooperation plans at city and county levels to balance the overall economic growth and the local emission reduction targets.

The study is not free from limitations. First, although the study set many scenarios to depict the trend of future carbon emission, as a result of the smooth setting of parameters, the scenario model may not be able to effectively capture the rebound effect of carbon emissions during a certain period, which is likely to occur with the increase of energy consumption during the economic recovery after COVID-19 pandemic. Actually, the increase in energy consumption caused by strong economic recovery in the post epidemic era may delay the time to achieve carbon peak to a certain extent. However, due to the large space for carbon peak, we are optimistic that China will finally achieve carbon peak by 2030. Second, due to the lack of city- and county-level sectoral carbon emission data, the study used the total carbon emission data of provinces, cities and counties and thus we did not analyze the carbon peak from the perspective of carbon emission structure. It is expected to make a breakthrough in developing city- and county-level sectoral carbon emission inventories in future, so as to better support carbon peak and carbon neutral policymaking. Third, although the study covered most provinces, cities and counties, there are still some cities and counties outside the carbon peak analysis due to the lack of data, leading to the weakened support for the full implementation of the regional priority carbon peak policy at the city and county level. Those deficiencies should be addressed in future studies.

## Methods

### Ensemble time-series forecasting model

The prediction based on TS encounters various uncertainties in the future. The prediction method based on ML can capture the nonlinear relationship of data changes at a high accuracy. Its explanatory power, however, is weak due to the “black box” in the operation process. In contrast, the traditional TS prediction method (i.e., non-ML method) usually has high explanatory power but unsatisfactory prediction accuracy relative to ML-based prediction methods. In this regard, integrating the two prediction methods is necessary to enhance the generalization ability and accuracy of TS forecasting.

**Figure 7:**
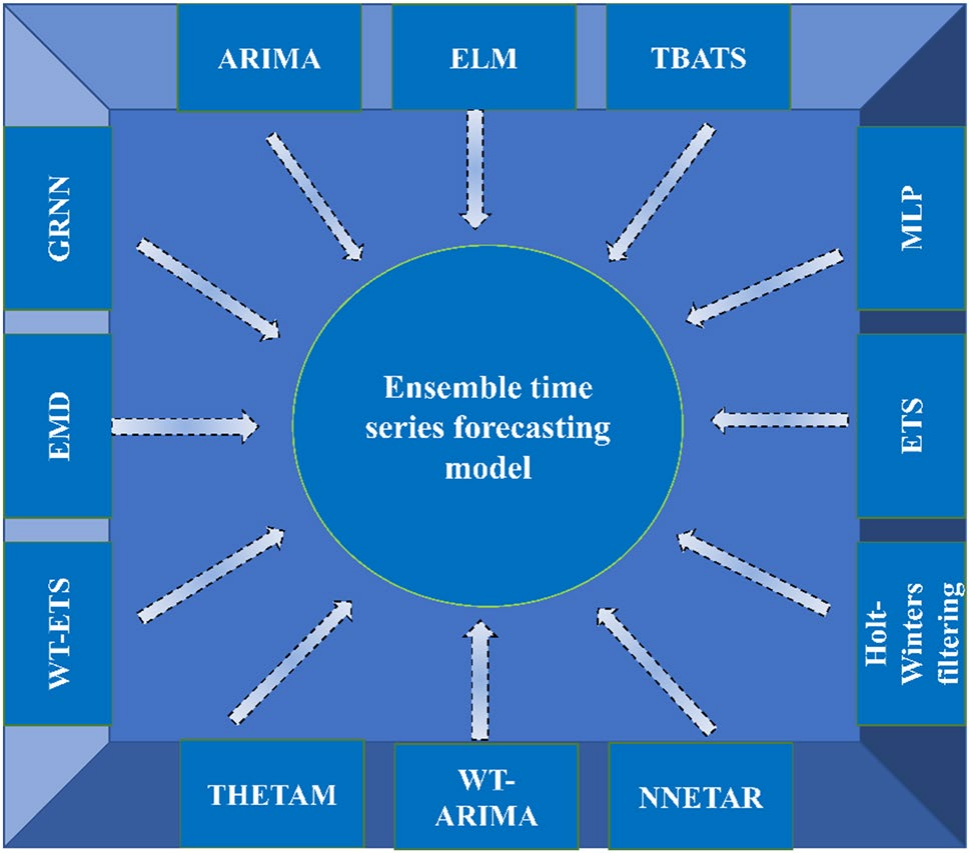
Framework for developing ensemble time-series forecasting model.

The ensemble TS forecasting model developed in the study consists of the following 12 methods (Fig. 7). The ML methods include the (1) extreme learning machine (ELM), (2) multilayer perceptron (MLP), (3) general regression neural network (GRNN), while the non-ML methods are the (4) autoregressive integrated moving average model (ARIMA), (5) Holt–Winters filtering, (6) empirical mode decomposition (EMD), (7) exponential smoothing state space model (ETS), (8) ARIMA-based wavelet transform (WT-ARIMA), (9) ETS-based wavelet transform (WT-ETS), (10) the theta method ‘model’ (THETAM), (11) feed-forward neural network TS forecast (NNETAR), and (12) exponential smoothing state space model with Box–Cox transformation, ARIMA errors, trend, and seasonal components (TBATS). ETS, THETAM, NNETAR, TBATS, and ARIMA were first integrated into a hybrid model in the forecastHybrid R package. Using the developed model, we projected detailed forecasts on China’s CO_2_ emission in county, city, and province levels by 2035. The details on the ensemble TS forecasting model can be seen in the supplemental method S1.

### Generalized Divisia decomposition approach

The GDIM proposed by Vaninsky^36^ was utilized to decompose the changes in aggregate CO2 emission in Chinese cities. In addition to GDIM approach, logarithmic mean Divisia index (LMDI)^37^ is another widely used decomposition approach. However, LMDI quantitatively describes the economic and population indicators while the intensity indicators (e.g., GDP per capita and per capita CO_2_ emission) are hardly analyzed in a single decomposition framework. In addition, the LMDI has different factorial decompositions due to varied factor models. On the contrast, The GDIM overcomes the disadvantages of LMDI above and has been used in energy or emission studies (e.g.,^22,38^).

Following the framework in Vaninsky^36^, we decompose the changes in China’s overall CO_2_ emission in 1997–2019 as follows:

1 $$ \begin{aligned} C & = \sum\limits_{i} {C_{i} } = \sum\limits_{i} {\frac{{C_{i} }}{{Y_{i} }} \times Y_{i} } = \sum\limits_{i} {\frac{{C_{i} }}{{P_{i} }} \times P_{i} } \\ & = \sum\limits_{i} {CI_{i} \times Y_{i} } = \sum\limits_{i} {PC_{i} \times P_{i} } \\ \end{aligned} $$

where $i$ represents a city ($i = 1, \, 2, \ldots ,262$); $C$ the CO_2_ emission, $Y$ the GDP, $P$ the population, $CI$ the carbon intensity, and $PC$ the CO_2_ emission per capita. Then, this equation is derived from Eq. (1):

2 $$ \frac{{Y_{i} }}{{P_{i} }} = \frac{{\left( {{{C_{i} } \mathord{\left/ {\vphantom {{C_{i} } {P_{i} }}} \right. \kern-\nulldelimiterspace} {P_{i} }}} \right)}}{{\left( {{{C_{i} } \mathord{\left/ {\vphantom {{C_{i} } {Y_{i} }}} \right. \kern-\nulldelimiterspace} {Y_{i} }}} \right)}} $$

To analyze the five factors, i.e., $CI$, $Y$, $PC$, $P$, and $PY$, in a single decomposition framework, we followed Vaninsky^36^, rewriting Eqs. (1) and (2) as follows:

3 $$ C_{i} = Y_{i} \cdot CI_{i} $$

4 $$ \Omega_{1} = Y_{i} \cdot CI_{i} - P_{i} \cdot PC_{i} = 0 $$

5 $$ \Omega_{2} = Y_{i} - P_{i} \cdot PY_{i} = 0 $$

In terms of $X$ ($X = \left[ {Y_{i} ,CI_{i} ,P_{i} ,PC_{i} ,PY_{i} } \right]$), the gradient of the function $C_{i} \left( X \right)$ and Jacobian matrix $\Phi_{X}$ are listed in Eqs. (6) and (7).

6 $$ \nabla C_{i} = \left\langle {CI_{i} ,Y_{i} ,0,0,0} \right\rangle^{T} $$

7 $$ \Phi_{X} = \left( \begin{gathered} CI_{i} \, Y_{i} \, - PC_{i} \, - P_{i} \, 0 \hfill \\ \, 1 \, 0 \, - PY_{i} \, 0 \, - P_{i} \hfill \\ \end{gathered} \right)^{T} $$

Due to the interconnections of different factors, decomposing changes in CO_2_ emission can be rewritten as the following:

8 $$ \Delta C_{i} \left[ {X|\Phi } \right] = \int_{Period} {\nabla C_{i}^{T} \left( {I - \Phi_{X} \Phi_{X}^{ + } } \right)dX} $$

where, in Eq. (8), $Period$ denotes the time span, $I$ the identity matrix, and “+” the generalized inverse matrix. When the columns of the matrix ${\Phi }_{X}$ satisfy the condition of linear independence, $\Phi_{X}^{ + } = \left( {\Phi_{X}^{T} \Phi_{X} } \right)^{ - 1} \Phi_{X}^{T}$.

Finally, changes in CO_2_ emission for city $i$ can be decomposed into the following drivers:

9 $$ \Delta C_{i} = \sum\limits_{m} {\Delta C_{i} \left( {X_{m} } \right)} $$

where $m$ denotes the corresponding drivers ($m = 1,2, \ldots ,5$). The change in CO_2_ emission for a specific city group can be decomposed as

10 $$ \Delta C_{g} = \sum\limits_{i}^{g} {\Delta C_{i} \left( {X_{m} } \right)} $$

where $g$ ($g = \left[ {g_{1} ,g_{2} , \cdots ,g_{n} } \right]$) denotes different city groups. Equations (9) and (10) consider five drivers, i.e., economic scale ($\Delta C_{Y}$), carbon intensity ($\Delta C_{CI}$), population ($\Delta C_{P}$), CO_2_ emission per capita ($\Delta C_{PC}$) and GDP per capita ($\Delta C_{PY}$).

### Gaussian Kuznets curve

According to the EKC theory^28,29^, pollution such as CO_2_ emission should increase with economic development and then decline after reaching a peak. Based on such assumption, we used this curve to link the CO_2_ emission and GDP in China.

The Gaussian Kuznets curve can be expressed as

11 $$ pc = a \cdot \exp \left[ { - \left( {\frac{py - b}{c}} \right)^{2} } \right] $$

where $pc$ denotes the CO_2_ emission per capita, $py$ the GDP per capita; parameters $a$, $b$, and $c$ reflect the peak CO_2_ emission per capita (maximum height of the function), the GDP per capita at vertex $a$ (position of the function along the horizontal axis), and the shape of the function, respectively.

We used the minpack.lm R package to obtain the abovementioned parameters for each province and city (see Figs. S3-1 and S3-2). Given that $py_{peak}$ in provinces and cities followed a normal distribution and logarithmic normal distribution in the study, we then obtained the overall peak by calculating the mean value from all provinces and cities at 70%, 80%, and 90% confidence intervals. We utilized the CO_2_ emission per capita in Eq. (11) as an exogenous variable to project China’s national CO_2_ emission peak at different confidence intervals.

Wang et al.^12^ also applied the same method to estimate China’s carbon peak based on 50 cities from 2000 to 2016. However, there remains uncertainty for estimating China’s carbon peak; hence, a large-scale study covering most cities and counties remains warranted. Moreover, due to recent changes in CO_2_ emission of China, a new and comprehensive analysis is required. The Chinese government conducted the priority-peak policy for China’s carbon peak while there remains no study quantifying the status quo of carbon peaks at local levels. The identification of carbon peaks at different levels, especially at city and county levels, is of great importance for formulating carbon peak strategies in the country and future carbon neutrality target by 2060.

For robust results, we estimated China’s overall carbon peak at provincial and city levels based on the updated datasets in 2019. Further, we classified the provinces, cities, and counties according to their position in the curve (Fig. 4d).

### Scenario analysis

The scenario analysis was conducted to consider the impacts of COVID-19 outbreak and the slump in CI decline. The scenarios were based on the changes in economic growth rates and CI, the greatest positive and negative drivers, respectively, contributing to the increase in CO_2_ emission based on the decomposition analysis.

To project the trajectories of CO_2_ emission, we made the following assumptions (supplemental method S1). We set three scenarios, namely the BAU, moderate, and advanced, to describe China’s economy in the next 15 years. In the BAU scenario, no significant changes in the emission reduction policies and technical progress will occur^39^. In the moderate scenario, the overall growth rate of the Chinese economy will be higher than that in the BAU scenario by implementing the double circulation strategy and increasing the investments in technological innovation. In the advanced scenario, a growth rate higher than that in the moderate scenario will occur by implementing an in-depth economic structural optimization and releasing high-tech benefits. We then calculated the economic AAGRs during the 13th FYP for the BAU scenario, both 12th and 13th FYP (2011–2020) for the moderate scenario and the 12th FYP for the advanced scenario. In the moderate scenario, we excluded the impact of the pandemic on the economy. Notably, using the latest 2020 economic growth data of China's economy improved the accuracy of scenarios and provided a new benchmark for carbon peak analysis. (Tables S2-1, S2-3, and S2-5).

We assumed three corresponding AAGRs to reduce the CI in 2021–2035 based on the three scenarios. In the BAU scenario, the AAGRs would be similar to the 13th FYP period, and the impact of the coronavirus on CI reduction would be short-term. In the moderate scenario, the AAGRs would be similar to those in the last decade (2011–2020), and the CI reduction would be less affected by the pandemic. In addition, low-carbon, energy-saving technologies, and new power generation factories would be established. In the advanced scenario, the AAGRs would be similar to those in the 12th FYP period, and strengthened CI reduction would be implemented as most provinces would exceed the targets during that period. The advanced scenario requires technological breakthroughs such as CCS and advanced nuclear energy technologies (Tables S2-2, S2-4 and S2-6).

### Social network analysis

SNA is an interdisciplinary analysis method for "relation data." SNA can be used for determining spatial pattern of many topics such as economic growth, energy consumption and carbon emission. In this study, we used SNA to capture the spatial pattern of interprovincial CO_2_ emission network in the post-pandemic era under the carbon peak background for China. According to Scott^40^ and Furht^41^, the network is defined as a group of nodes connected by links, in which “nodes” in the network indicate “participants”. “Nodes” in the study refer to “provinces” and thus “connection” represents the relationship between provinces.

To analyze the complex interprovincial carbon emission network, we use provincial CO2 emission data as the network “node”, and defined the “line” between two nodes in the network as spatial correlation of carbon emission. Similar to previous studies (e.g.,^42^), we used a modified gravity model to construct the spatial correlation of interprovincial carbon emission in China as follows:

12 $$ y_{ij} = \frac{{C_{i} }}{{C_{i} + C_{j} }} \times \frac{{\sqrt[3]{{P_{i} C_{i} G_{i} }} \times \sqrt[3]{{P_{j} C_{i} G_{j} }}}}{{\left( {\frac{{D_{ij} }}{{g_{i} - g_{j} }}} \right)^{2} }} $$

where $i$ and $j$ are compared provinces; $y_{ij}$ is the gravitation of carbon emission between province $i$ and province $j$; $C$ is carbon emission; $P$ and $G$ denote population scale and GDP; $g$ and $D$ represent GDP per capita and the spherical distance between the provincial capitals; $\frac{{C_{i} }}{{C_{i} + C_{j} }}$ reflects the gravity coefficient of carbon emission from province $i$ to province $j$.

Based on Eq. (12), we can construct the gravity matrix of interprovincial carbon emission and obtain the complex interprovincial carbon emission network above. We then further analyzed the network characteristics with emphasis on the overall network characteristics and individual network characteristics. We use network tie, network density, network hierarchy and network efficiency to describe the overall network characteristics, and use degree centrality, betweenness centrality and closeness centrality to analyze the individual networks characteristics (supplemental method S1).

### Data process

The CO_2_ emission data ($C$) of the provinces were collected from Shan et al.^43^ and Shan et al.^44^ while that of the cities and counties were gathered from Chen et al.,^45^. Furthermore, we updated the dataset of China’s CO_2_ emissions in 2018–2019 at all levels using a top-down approach where we found that the annual ratios of CO_2_ emissions at all levels to the national CO_2_ emission does not change significantly. We, therefore, assumed that the ratios in most areas at all levels would follow their changing trends in 2018 and 2019. We then used Holt–Winters filter method to forecast the CO_2_ emission at all levels. We found that the forecasting errors of aggregated CO_2_ emissions were 0.01% in 2018 and 2019 in provinces, − 0.10% in 2018 and − 0.08% in 2019 in cities, and 0.12% in 2018 and 0.27% in 2019 in counties (Supplemental data S1).

The GDP ($Y$) data of provinces, cities, and counties were obtained from the NBSC, China Premium Database (CEIC)^46^, and China County Statistical Yearbook (1999–2019)^47^, respectively. The population ($P$) data of the provinces were obtained from the NBSC, while that of the cities and counties were collected from the CEIC and China Stock Market Accounting Research (CSMAR)^48^, respectively, in which some missing values were completed by spline interpolation as the resident population of a place usually would not change dramatically during a certain period. The future population at provincial level in SNA analysis was collected from Chen et al.^49^. To minimize the impact of missing data on the analysis, we used the datasets from the provinces in 1997–2019, cities in 2002–2019, and counties in 2003–2018.

To determine the drivers of CO_2_ emission changes, we classified the cities into nine groups based on population scale and economic structure. Following the Chinese government’s classification scheme in 2014^50^, the cities were grouped into megacities (population of > 10 million), very large cities (population of 5–10 million), large cities (population of 3–5 million), midsize cities-I (population of 1–3 million), midsize cities-II (population of 0.5–1 million), and small cities (population of < 0.5 million). Similar to Ramaswami et al.^51^ and Tong et al.^52^, we also divided the cities into three city groups by economic structure: the highly industrial in which the secondary industrial GDP% was higher than the national average plus one standard deviation, highly commercial where the tertiary industrial GDP percentage was higher than the national average plus one standard deviation, and mixed-economy cities that did not fall in the abovementioned two types. We also classified the provinces into three regions, i.e., the eastern, central and western regions according to Chen et al.^53^.

**Figure 8:**
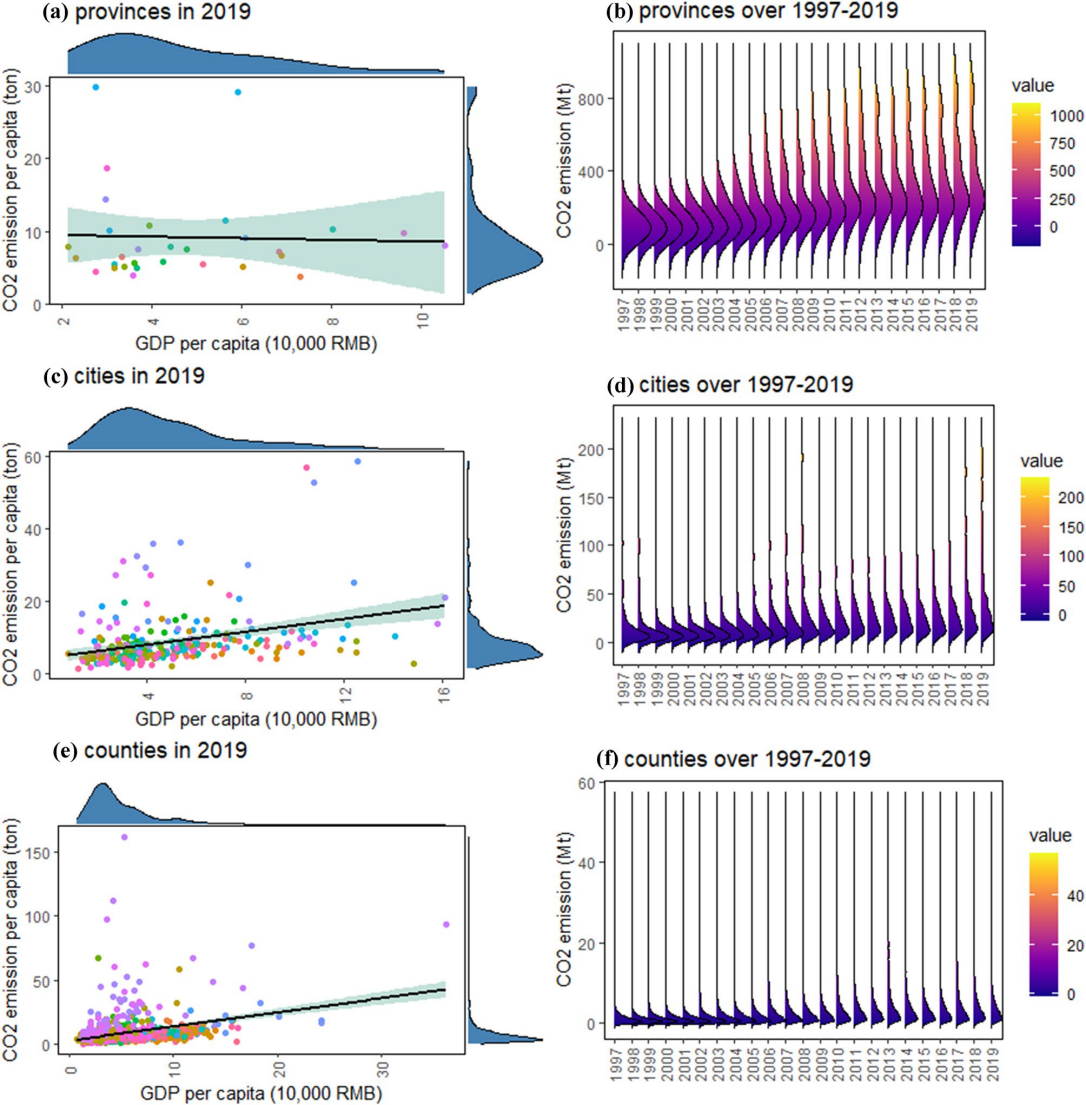
Changes in CO_2_ emissions of Chinese provinces, cities and counties over 1997–2019 and the corresponding relationships between GDP per capita and CO_2_ emission per capita in 2019. (**a**,**c**,**e**) The relationships between GDP per capita and CO_2_ emission per capita for 30 provinces, 262 cities and 928 counties in mainland China in 2019, in which GDP per capita was adjusted at the constant prices for provinces in 1997 and for cities in 2002, due to the completeness and availability of data. (**b**,**d**,**f**) Cover 30 provinces, 292 cities and 2735 counties in mainland China.

Figure 8a,c,e described the relationships between GDP per capita and CO_2_ emission per capita across regions in 2019, implying that there may exist a simple relationship between the two variables above despite the skewed spatial distributions. Further, Fig. 8b,d,f depicted the changing trends of CO_2_ emission in Chinese provinces, cities and counties over 1997–2019, indicating carbon emissions among regions also presented skewed distributions with increasing trends over time. Therefore, the heterogeneity of carbon emissions at different levels should not be neglected in carbon peak analysis.

## Supplementary Information

Supplementary Information.

## Data Availability

See the data process section for historical CO_2_ emission, GDP, population at all levels in the study and the estimated CO_2_ emission in 2018 and 2019 at all levels are available from the corresponding authors upon request.

## Code availability

The code that support the findings of this study are available from the corresponding authors upon request.
