# Supplementary material for: Carbon peak and its mitigation implications for China in the post-pandemic era
Source: Sci Rep. 2022 Mar 2;12:3473. doi: 10.1038/s41598-022-07283-4 (PMC8891329; doi:10.1038/s41598-022-07283-4)
Supplement: Supplementary file 1 — Supplementary Information. [file 41598_2022_7283_MOESM1_ESM.doc]

**Supporting Information for**

**Carbon peak and its mitigation implications for China in the post-pandemic era**

Authors:

Jiandong Chen^#^, Chong Xu^#^, Ming Gao, Ding Li^*^

Author Affiliations:

School of Public Administration, Southwestern University of Finance and Economics, Chengdu, China

^#^ The authors (Jiandong Chen and Chong Xu) contributed equally to this study.

^*^ Correspondence: liding@vip.sina.com

This document is 31 pages long. It contains 9 Tables and 8 Figures.

The Supporting Information (SI) provides additional information on: (1) Supplementary method; (2) Assumptions on average annual growth rates of economic growth and carbon intensity in China (2021-2035); (3) Gaussian Kuznets curve regression results; (4) Illustrations of category division of cities; (5) Sensitivity analysis.

**S1 Supplementary method**

**Ensemble time-series forecasting model**

The ensemble time-series forecasting model developed in the study consists of the following 12 methods (see Fig. 1 for illustration in the main text): The ML methods include the (1) extreme learning machine (ELM), (2) multilayer perceptron (MLP), (3) general regression neural network (GRNN), while the non-ML methods are the (4) autoregressive integrated moving average model (ARIMA), (5) Holt–Winters filtering, (6) empirical mode decomposition (EMD), (7) exponential smoothing state space model (ETS), (8) ARIMA-based wavelet transform (WT-ARIMA), (9) ETS-based wavelet transform (WT-ETS), (10) the theta method ‘model’ (THETAM), (11) feed-forward neural network TS forecast (NNETAR), and (12) exponential smoothing state space model with Box–Cox transformation, ARIMA errors, trend, and seasonal components (TBATS). ETS, THETAM, NNETAR, TBATS, and ARIMA were first integrated into a hybrid model in the forecastHybrid R package.

ELM is a kind of feedforward neural network, with the advantage that has good generalization performance and fast learning ability (Huang et al. 2006). ELM does not need back propagation based on gradient to adjust weights, but uses Moore Penrose generalized inverse to set weights. MLP is a neural network with feedforward structure to map a set of input vectors to a set of output vectors. The supervised learning method of back propagation algorithm is usually used to train MLP. MLP is a generalization of perceptron, overcoming the weakness that perceptron can't recognize linear indivisible data. GRNN is a radial basis function network based on mathematical statistics, which has good nonlinear approximation performance. A comparison on the three neural networks above can be seen Fig. S1-1. The further details can be seen in Huang et al. (2006), Ord et al. (2017) and Specht (1991). Further, the details on ETS, THETAM, NNETAR, TBATS can be referenced in forecastHybrid package. The details on WT-ARIMA and WT-ETS can be seen in Conejo et al. (2005), Joo and Kim (2015), Salles et al. (2019) and Stolojescu et al. (2010).

ARIMA model and Holt-Winters are two widely used models for time-series forecasting (Box et al. 2011; Holt, 1957; Winters, 1960). An ARIMA model can be determined by three parameters, i.e., p, d and q, where p denotes the number of autoregressive (AR), d presents the number of differences, q means the number of moving average (MA). If the time series is non-stationary due to the stochastic factors, the difference can be made according to the time period, and then ARIMA model can be transformed into ARMA model. Generally, ARIMA model can be written as follows:

$\left(1-\sum_{i=1}^{p} \Phi_{i}L^{i}\right)\left(1-L\right)^{d}X_{t}=\left(1+\sum_{i=1}^{q} \varpi_{i}L^{i}\right)\varepsilon_{t}$ (1)

where in Eq. (1), L is the lag operator, d is the positive number.

There are additive and multiplicative Holt-Winters Filtering models in time-series forecasting. Considering a time-series data ($X_{t}$) consists of trend component ($w_{t}$) and seasonal component ($s_{t}$), then the difference between the two Holt-Winters Filtering models lies in that the relationship between $w_{t}$ and $s_{t}$ is assumed to be additive for the additive model while the multiplicative model assumes the corresponding relationship is multiplicative. The additive Holt-Winters Filtering model can be expressed as follows:

$\left\{\begin{matrix} w_{t}=\alpha\times\left(X_{t}-s_{t-T}\right)+\left(1-\alpha\right)\times\left(w_{t-1}+v_{t-1}\right), \\ v_{t}=\beta\times\left(w_{t}-w_{t-1}\right)+\left(1-\beta\right)\times v_{t-1}, \\ s_{t}=\eta\times\left(X_{i}-w_{i}\right)+\left(1-\eta\right)\times s_{t-T} \\ X_{i+h}=w_{i}+h*v_{i}+s_{i-T+h} \end{matrix}\right.$ (2)

The multiplicative Holt-Winters Filtering model can be expressed as follows:

$\left\{\begin{matrix} w_{t}=\alpha\times\left(X_{t}/s_{t-T}\right)+\left(1-\alpha\right)\times\left(w_{t-1}+v_{t-1}\right), \\ v_{t}=\beta\times\left(w_{t}-w_{t-1}\right)+\left(1-\beta\right)\times v_{t-1}, \\ s_{t}=\eta\times\left(X_{i}/w_{i}\right)+\left(1-\eta\right)\times s_{t-T} \\ X_{i+h}=\left(w_{i}+h*v_{i}\right)s_{i-T+h} \end{matrix}\right.$ (3)

Where in Eqs. (2) and (3), $\alpha$, $\beta$ and $\eta$ are parameters ranging from 0 to 1, $v_{t}$ represents the linear increasing rate of the trend component $w_{t}$.

By those time-series forecasting models above, we can predict CO_2_ emission trajectory in future. Fig. S1-2 showed an example of Chengdu city, the largest city in western China. The models show both downwards and upwards trends of CO_2_ emission. To improve the he generalization ability and accuracy of time-series forecasting, we thus developed an ensemble model by weighting different forecasting models in the study. It should be noted that It is almost impossible to predict future changes perfectly and accurately. Therefore, the ensemble model in the study is only used as a benchmark in scenario analysis.


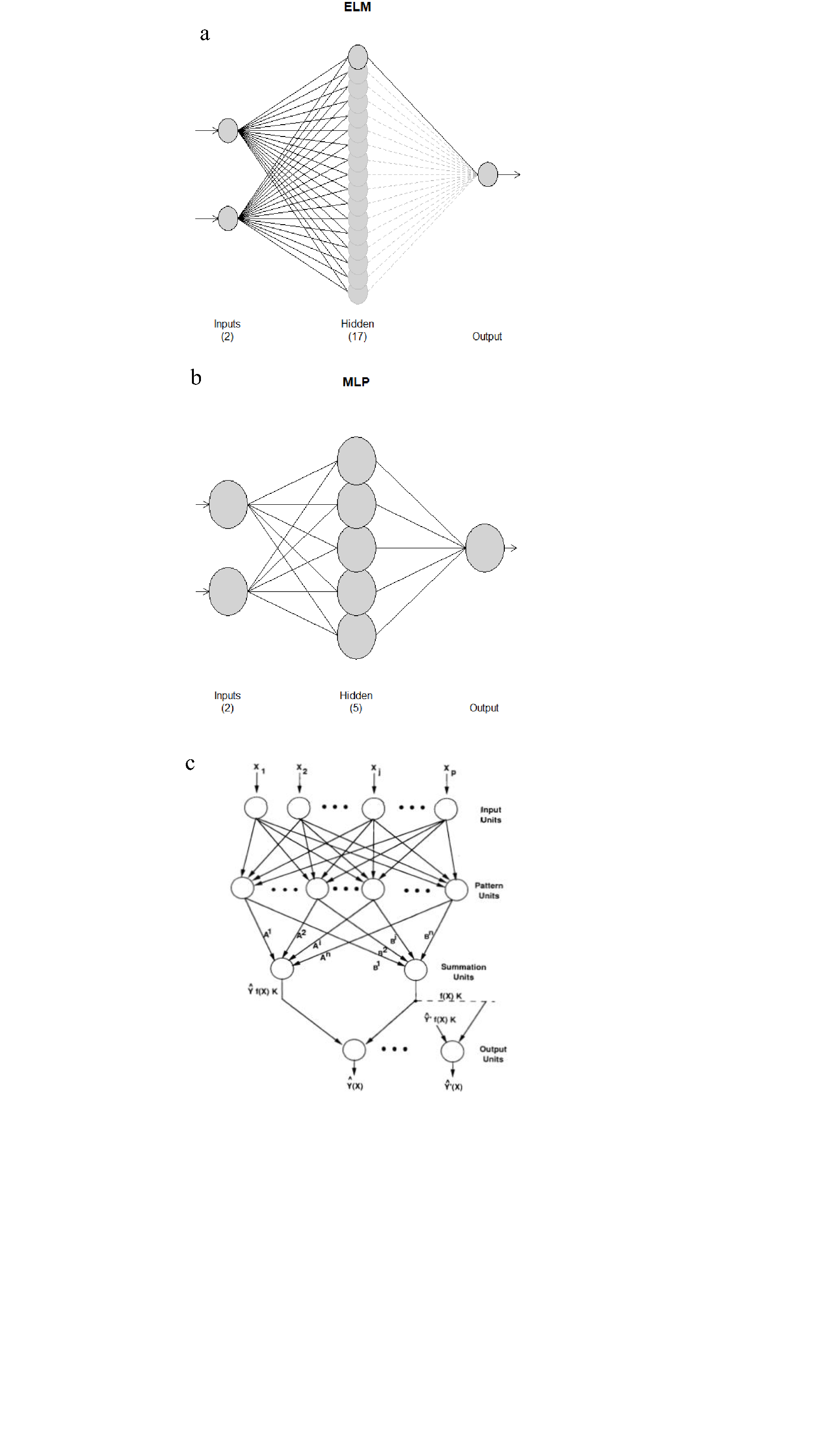


**Figure S1-1 An illustration of structure of extreme learning machine (a), multilayer perceptron (b) and general regression neural network (c). Note that Fig. S1-1c sources from Specht (1991).**


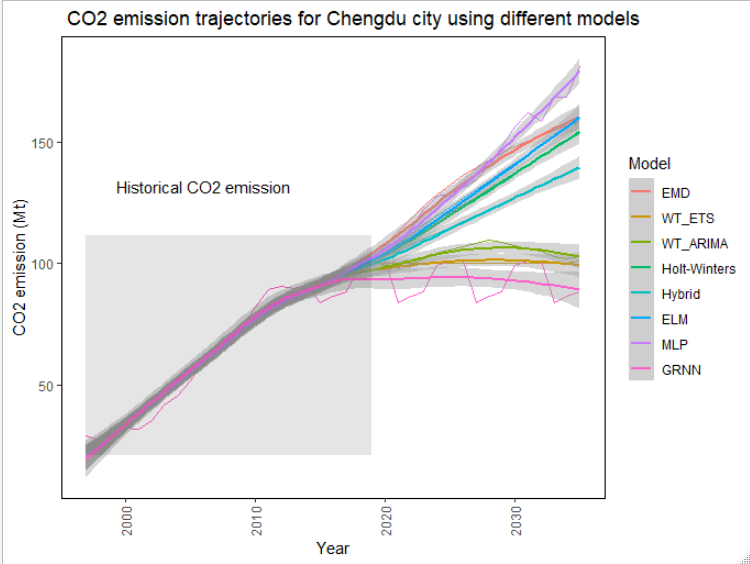


**Figure S1-2 An illustration of CO_2_ emission trajectories for Chengdu city using different time-series foresting model in 1997-2035.**

**Scenario analysis**

The scenario analysis was conducted to consider the COVID-19 outbreak and the slump in CI decline. The scenarios were based on the changes in economic growth rates and CI, the greatest positive and negative drivers, respectively, contributing to the increase in CO_2_ emission based on the decomposition analysis.

To project the trajectories of CO_2_ emission, we made the following assumptions. First, we calculated the economic average annual growth rates (AAGRs) at different periods, to Zhang et al. (2020) and Chen et al. (2020a). We considered the 12th (2011–2015) and 13th (2016–2020) five-year plan (FYP) periods. China’s economic growth has declined (Chen et al., 2020b), and therefore, it is reasonable to assume that the high growth rate (about 10%) of the Chinese economy in the past is unlikely in the next decade, but future economic growth may be similar to the recent periods, especially the 13^th^ FYP.

As the COVID-19 pandemic damaged the global economy (McKibbin and Fernando, 2020), the Chinese economy inevitably suffered a sharp decline. Some institutions estimated the growth rate of China in 2020 fell to 1%–3% (World Bank; IMF; Institute for Advanced Research of Shanghai University of Finance and Economics, IAR-SUFE; NBSC; China Macroeconomy Forum, CMF). However, the Chinese economy is predicted to recover at a high growth rate (8.2% by the IMF) in 2021. Hence, the COVID-19 pandemic will not fundamentally change the overall trend of China’s economic growth in the long term. We thus assumed that the average economic growth in the next 15 years (2021–2035) would follow the general trend.

We set three scenarios, i.e., the business-as-usual (BAU), moderate, and advanced, to describe China’s economy in the next 15 years. In the BAU scenario, no significant changes in the emission reduction policies and technical progress will occur (Chen et al., 2020a). In the moderate scenario, the overall growth rate of the Chinese economy will be higher than that in the BAU scenario by implementing the double circulation strategy and increasing the investments in technological innovation. In the advanced scenario, a growth rate higher than that in the moderate scenario will occur by implementing an in-depth economic structural optimization and releasing high-tech benefits. We then calculated the economic AAGRs during the 13th FYP for the BAU scenario, both 12th and 13th FYP (2011–2020) for the moderate scenario and the 12th FYP for the advanced scenario. In the moderate scenario, we excluded the impact of the pandemic on the economy. It is noteworthy that using the latest 2020 economic growth data of China's economy improved the accuracy of scenarios and provided a new benchmark for carbon peak analysis. The details on the AAGRs of economic growth during 2021-2035 can be found in Tables S2-1, S2-3 and S2-5.

Although China exceeded its CI reduction target in the 2009 Copenhagen Climate Change Conference, many provinces slowed their declining rates of CI during the 13th FYP period (Department of Ecology and Environment in Inner Mongolia, 2020). Those provinces may finally achieve the targets in the next period as coronavirus has decreased the global CO_2_ emission to 6.4% (Liu et al., 2020). However, the coronavirus outbreak was sudden, and the CI would gradually decrease if other factors do not change.

We assumed three corresponding AAGRs to reduce the CI in 2021–2035 based on the three scenarios. In the BAU scenario, the AAGRs would be similar to the 13th FYP period, and the impact of the coronavirus on CI reduction would be short-term. In the moderate scenario, the AARGs would be similar to those in the last decade (2011–2020), and the CI reduction would be less affected by the pandemic. In addition, low-carbon, energy-saving technologies, and new power generation factories would be established. In the advanced scenario, the AAGRs would be similar to those in the 12th FYP period, and strengthened CI reduction would be implemented as most provinces would exceed the targets during that period. The advanced scenario requires technological breakthroughs such as carbon capture and storage (CCS) and advanced nuclear energy technologies. The details on the AAGRs of economic growth during 2021-2035 can be seen in Tables S2-2, S2-4 and S2-6.

**Table S1-1**

**Defininations of different scenarios on economic growth and decline in carbon intensity in the study.**

| Object | Basic scenario | Description | Parameters |
| --- | --- | --- | --- |
| Economic growth | BAU | No significant changes in the emission reduction policies and technical progress will occur | The economic AAGRs during the 13th FYP (2016-2020) |
|  | Moderate | The overall growth rate of the Chinese economy will be higher than that in the low scenario by implementing the double circulation strategy and increasing the investments in technological innovation | The economic AAGRs during the 12th and 13th FYP (2011–2020) |
|  | Advanced | A growth rate higher than that in the middle scenario will occur by implementing an in-depth economic structural optimization and releasing high-tech benefits | The economic AAGRs during the 12th FYP (2011-2015) |
| Carbon intensity | BAU | The decline in carbon intensity would be as-usual, and the impact of the coronavirus on CI reduction would be short-term | The carbon intensity AAGRs during 13th FYP (2016-2020) |
|  | Moderate | The CI reduction would be less affected by the pandemic. Low-carbon, energy-saving technologies, and new power generation factories would be established | The carbon intensity AAGRs during the 12th and 13th FYP (2011–2020) |
|  | Advanced | Strengthened CI reduction would be implemented as most provinces would exceed the targets during that period, requring technological breakthroughs such as carbon capture and storage (CCS) and advanced nuclear energy technologies. | The carbon intensity AAGRs during 12th FYP (2011-2015) |

Note: AAGRs denotes average annual growth rates; CI deotes carbon intensity; to consider the historical effects and uncertainties of policy implementations in accordance with previous studies (e.g., Zhang et al. (2020) and Chen et al. (2020a)), we further set baseline and best levels based on the middle level of economic growth AAGRs for each basic scenario, with 1 percent fluctuation of the middle level, respectively (see Tables S2-1, S2-3 and S2-5 for details); we set baseline and best levels based on the middle level of carbon intenisty AAGRs for each basic scenario according to different esitamtions of carbon intensity during the sample period (see Tables S2-2, S2-4 and S2-6 for details). In the scenario analysis, parameters are used as described in Table S5-1 since the decomposition results indicated that CI reduction is more important for reducing CO_2_ emission in Chinese provinces and cities categorized by population size and economic structure in the post-Kyoto era.

**Social network analysis**

Social network analysis (SNA) is an interdisciplinary analysis method for "relation data". This study here used SNA to capture the spatial pattern of interprovincial CO_2_ emission network in the post-pandemic era under the carbon peak background for China.

According to Scott (1988) and Furht (2010), the network is defined a group of nodes connected by links, in which “node” in the network are “participants”. “Node” in the study refer to “provinces” and thus “connection” means the relationship between the provinces.

To analyze the complex interprovincial carbon emission network, we use provincial CO_2_ emission data as the network “node”, and defined the “line” between two nodes in the network as spatial correlation of carbon emission. Similar to previous studies (e.g., Bu et al. (2020)), we used a modified gravity model to construct the spatial correlation of interprovincial carbon emission in China as follows:

(4)

where in Eq. (4), $i$ and $j$ are compared provinces; $y_{ij}$ is the gravitation of carbon emission between province $i$ and province $j$; $C$ are carbon emission; $P$ and $G$denote population scale and gross domestic production (GDP); $g$ and $D$ represent GDP per capita and the spherical distance between the provincial capitals; $\frac{C_{i}}{C_{i}+C_{j}}$ reflects the gravity coefficient of carbon emission from province $i$ to province $j$.

Based on Eq. (4), we can construct the gravity matrix of interprovincial carbon emission and obtain the complex interprovincial carbon emission network above. We then further analyzed the network characteristics with emphasis on the overall network characteristics and individual network characteristics.

The study uses network tie, network density, network hierarchy and network efficiency to describe the overall network characteristics.

Network tie refers to the number of all actual network connections.

Network density can be calculated as follows:

$D=\frac{M}{N\cdot\left(N-1\right)}$ (5)

where $D$ is the density, $N$ and $M$ are the number of nodes in the network and network tie. Network density reflects the degree of spatial correlation of carbon intensity. The larger the value is, the closer the relationship the interprovincial carbon emission is, and thus the network structure of spatial correlation of carbon intensity has a greater impact on the carbon emission of each province.

Network hierarchy can be calculated as:

$H=1-\frac{K}{Max(K)}$ (6)

where in Eq. (6), $H$ is the hierarchy, $K$ is the number of groups. Network hierarchy refers to the degree of asymmetric accessibility in network nodes. The higher the network hierarchy is, the more strict the hierarchical structure of carbon emission spatial correlation network is.

Network efficiency can be calculated as:

Network efficiency reflects the number of carbon emission spatial correlation channels. The lower the efficiency is, the more carbon emission spatial correlation channels are, the more stable the carbon emission spatial correlation network is.

Individual networks are characterized by degree centrality, betweenness centrality and closeness centrality.

Degree centrality can be expressed as:

$De=\frac{L}{N\cdot\left(N-1\right)-1}$ (7)

where in Eq. (7), $De$ represents the degree centrality and $L$ means the number of provinces directly connected to a specific province. Degree centrality describes the degree to which each province's carbon emission spatial correlation network is in the center. The more connections in the spatial correlation network, the higher degree centrality, which indicates that the province has more connections with other provinces in the carbon emission spatial correlation network, and the province is also in the center of the network. The in degree and out degree are degree centrality indicators when input data is not symmetric (see Scoot (1998) for further details).

Betweenness centrality can be expressed as:

$C_{b}=\sum_{j}^{n} \sum_{k}^{n} b_{jk}\left(i\right)$ for $j\neq k\neq i$ and $j<k$ (8)

where $C_{b}$ and $b_{jk}$ represent betweenness centrality and the ability of node $i$ to affect the connection between nodes $j$ and $k$. Betweenness centrality measures the degree to which a province controls the relationship between other provinces. The higher the betweenness centrality, the more the province can affect the interaction between other provinces' carbon emission, and the more the province is in the center of the network.

Closeness centrality can be expressed as:

$C_{C}^{-1}=\sum_{j=1}^{n} d_{ij}$ (9)

where in Eq. (9) $C_{C}^{-1}$ and $d_{ij}$ represent closeness centrality and the distance between node $i$ and $j$.

Closeness centrality describes the degree to which a province is "not controlled by other provinces" in the spatial correlation network of carbon emission. The higher the closeness centrality is, there are more direct correlations between the province's carbon emission and other provinces.

**S2 Assumptions on average annual growth rates of economic growth and carbon intensity in China (2021-2035).**

**Table S2-1**

Assumptions on average annual growth rates of economic growth in China based on business-as-usual (BAU) scenario (Unit: %).

|  | 2021-2025 | | |  | 2026-2030 | | |  | 2031-2035 | | |
| --- | --- | --- | --- | --- | --- | --- | --- | --- | --- | --- | --- |
| Province | Best | Middle | Baseline |  | Best | Middle | Baseline |  | Best | Middle | Baseline |
| Beijing | 6.43 | 5.43 | 4.43 |  | 6.43 | 5.43 | 4.43 |  | 6.43 | 5.43 | 4.43 |
| Tianjin | 4.52 | 3.52 | 2.52 |  | 4.52 | 3.52 | 2.52 |  | 4.52 | 3.52 | 2.52 |
| Hebei | 6.53 | 5.53 | 4.53 |  | 6.53 | 5.53 | 4.53 |  | 6.53 | 5.53 | 4.53 |
| Shanxi | 6.53 | 5.53 | 4.53 |  | 6.53 | 5.53 | 4.53 |  | 6.53 | 5.53 | 4.53 |
| Inner Mongolia | 5.17 | 4.17 | 3.17 |  | 5.17 | 4.17 | 3.17 |  | 5.17 | 4.17 | 3.17 |
| Liaoning | 5.39 | 4.39 | 3.39 |  | 5.39 | 4.39 | 3.39 |  | 5.39 | 4.39 | 3.39 |
| Jilin | 4.74 | 3.74 | 2.74 |  | 4.74 | 3.74 | 2.74 |  | 4.74 | 3.74 | 2.74 |
| Heilongjiang | 5.34 | 4.34 | 3.34 |  | 5.34 | 4.34 | 3.34 |  | 5.34 | 4.34 | 3.34 |
| Shanghai | 6.48 | 5.48 | 4.48 |  | 6.48 | 5.48 | 4.48 |  | 6.48 | 5.48 | 4.48 |
| Jiangsu | 6.56 | 5.56 | 4.56 |  | 6.56 | 5.56 | 4.56 |  | 6.56 | 5.56 | 4.56 |
| Zhejiang | 6.98 | 5.98 | 4.98 |  | 6.98 | 5.98 | 4.98 |  | 6.98 | 5.98 | 4.98 |
| Anhui | 7.55 | 6.55 | 5.55 |  | 7.55 | 6.55 | 5.55 |  | 7.55 | 6.55 | 5.55 |
| Fujian | 7.55 | 6.55 | 5.55 |  | 7.55 | 6.55 | 5.55 |  | 7.55 | 6.55 | 5.55 |
| Jiangxi | 7.92 | 6.92 | 5.92 |  | 7.92 | 6.92 | 5.92 |  | 7.92 | 6.92 | 5.92 |
| Shandong | 6.36 | 5.36 | 4.36 |  | 6.36 | 5.36 | 4.36 |  | 6.36 | 5.36 | 4.36 |
| Henan | 7.15 | 6.15 | 5.15 |  | 7.15 | 6.15 | 5.15 |  | 7.15 | 6.15 | 5.15 |
| Hubei | 7.32 | 6.32 | 5.32 |  | 7.32 | 6.32 | 5.32 |  | 7.32 | 6.32 | 5.32 |
| Hunan | 7.40 | 6.40 | 5.40 |  | 7.40 | 6.40 | 5.40 |  | 7.40 | 6.40 | 5.40 |
| Guangdong | 6.68 | 5.68 | 4.68 |  | 6.68 | 5.68 | 4.68 |  | 6.68 | 5.68 | 4.68 |
| Guangxi | 6.53 | 5.53 | 4.53 |  | 6.53 | 5.53 | 4.53 |  | 6.53 | 5.53 | 4.53 |
| Hainan | 6.21 | 5.21 | 4.21 |  | 6.21 | 5.21 | 4.21 |  | 6.21 | 5.21 | 4.21 |
| Chongqing | 6.95 | 5.95 | 4.95 |  | 6.95 | 5.95 | 4.95 |  | 6.95 | 5.95 | 4.95 |
| Sichuan | 7.45 | 6.45 | 5.45 |  | 7.45 | 6.45 | 5.45 |  | 7.45 | 6.45 | 5.45 |
| Guizhou | 8.43 | 7.43 | 6.43 |  | 8.43 | 7.43 | 6.43 |  | 8.43 | 7.43 | 6.43 |
| Yunnan | 8.16 | 7.16 | 6.16 |  | 8.16 | 7.16 | 6.16 |  | 8.16 | 7.16 | 6.16 |
| Shaanxi | 7.07 | 6.07 | 5.07 |  | 7.07 | 6.07 | 5.07 |  | 7.07 | 6.07 | 5.07 |
| Gansu | 5.54 | 4.54 | 3.54 |  | 5.54 | 4.54 | 3.54 |  | 5.54 | 4.54 | 3.54 |
| Qinghai | 6.73 | 5.73 | 4.73 |  | 6.73 | 5.73 | 4.73 |  | 6.73 | 5.73 | 4.73 |
| Ningxia | 6.83 | 5.83 | 4.83 |  | 6.83 | 5.83 | 4.83 |  | 6.83 | 5.83 | 4.83 |
| Xinjiang | 6.53 | 5.53 | 4.53 |  | 6.53 | 5.53 | 4.53 |  | 6.53 | 5.53 | 4.53 |

**Table S2-2**

**Assumptions on average annual growth rates of carbon intensity in China based on business-as-usual (BAU) scenario (Unit: %).**

|  | 2021-2025 | | |  | 2026-2030 | | |  | 2031-2035 | | |
| --- | --- | --- | --- | --- | --- | --- | --- | --- | --- | --- | --- |
| Province | Best | Middle | Baseline |  | Best | Middle | Baseline |  | Best | Middle | Baseline |
| Beijing | -9.30 | -8.48 | -7.86 |  | -9.30 | -8.48 | -7.86 |  | -9.30 | -8.48 | -7.86 |
| Tianjin | -4.38 | -3.52 | -2.86 |  | -4.38 | -3.52 | -2.86 |  | -4.38 | -3.52 | -2.86 |
| Hebei | -4.91 | -4.05 | -3.40 |  | -4.91 | -4.05 | -3.40 |  | -4.91 | -4.05 | -3.40 |
| Shanxi | -2.73 | -1.86 | -1.19 |  | -2.73 | -1.86 | -1.19 |  | -2.73 | -1.86 | -1.19 |
| Inner Mongolia | -0.39 | 0.51 | 1.20 |  | -0.39 | 0.51 | 1.20 |  | -0.39 | 0.51 | 1.20 |
| Liaoning | -3.82 | -2.96 | -2.29 |  | -3.82 | -2.96 | -2.29 |  | -3.82 | -2.96 | -2.29 |
| Jilin | -4.37 | -3.52 | -2.85 |  | -4.37 | -3.52 | -2.85 |  | -4.37 | -3.52 | -2.85 |
| Heilongjiang | -5.10 | -4.25 | -3.60 |  | -5.10 | -4.25 | -3.60 |  | -5.10 | -4.25 | -3.60 |
| Shanghai | -6.42 | -5.58 | -4.93 |  | -6.42 | -5.58 | -4.93 |  | -6.42 | -5.58 | -4.93 |
| Jiangsu | -4.12 | -3.26 | -2.60 |  | -4.12 | -3.26 | -2.60 |  | -4.12 | -3.26 | -2.60 |
| Zhejiang | -4.48 | -3.63 | -2.97 |  | -4.48 | -3.63 | -2.97 |  | -4.48 | -3.63 | -2.97 |
| Anhui | -5.09 | -4.25 | -3.59 |  | -5.09 | -4.25 | -3.59 |  | -5.09 | -4.25 | -3.59 |
| Fujian | -2.81 | -1.94 | -1.27 |  | -2.81 | -1.94 | -1.27 |  | -2.81 | -1.94 | -1.27 |
| Jiangxi | -4.26 | -3.40 | -2.74 |  | -4.26 | -3.40 | -2.74 |  | -4.26 | -3.40 | -2.74 |
| Shandong | -4.87 | -4.02 | -3.37 |  | -4.87 | -4.02 | -3.37 |  | -4.87 | -4.02 | -3.37 |
| Henan | -6.11 | -5.27 | -4.62 |  | -6.11 | -5.27 | -4.62 |  | -6.11 | -5.27 | -4.62 |
| Hubei | -5.31 | -4.46 | -3.80 |  | -5.31 | -4.46 | -3.80 |  | -5.31 | -4.46 | -3.80 |
| Hunan | -4.34 | -3.49 | -2.82 |  | -4.34 | -3.49 | -2.82 |  | -4.34 | -3.49 | -2.82 |
| Guangdong | -3.78 | -2.92 | -2.26 |  | -3.78 | -2.92 | -2.26 |  | -3.78 | -2.92 | -2.26 |
| Guangxi | -3.08 | -2.22 | -1.55 |  | -3.08 | -2.22 | -1.55 |  | -3.08 | -2.22 | -1.55 |
| Hainan | -2.20 | -1.33 | -0.65 |  | -2.20 | -1.33 | -0.65 |  | -2.20 | -1.33 | -0.65 |
| Chongqing | -4.87 | -4.02 | -3.36 |  | -4.87 | -4.02 | -3.36 |  | -4.87 | -4.02 | -3.36 |
| Sichuan | -6.38 | -5.55 | -4.90 |  | -6.38 | -5.55 | -4.90 |  | -6.38 | -5.55 | -4.90 |
| Guizhou | -5.74 | -4.90 | -4.25 |  | -5.74 | -4.90 | -4.25 |  | -5.74 | -4.90 | -4.25 |
| Yunnan | -4.16 | -3.30 | -2.64 |  | -4.16 | -3.30 | -2.64 |  | -4.16 | -3.30 | -2.64 |
| Shaanxi | -5.16 | -4.31 | -3.66 |  | -5.16 | -4.31 | -3.66 |  | -5.16 | -4.31 | -3.66 |
| Gansu | -4.20 | -3.34 | -2.68 |  | -4.20 | -3.34 | -2.68 |  | -4.20 | -3.34 | -2.68 |
| Qinghai | -5.00 | -4.15 | -3.50 |  | -5.00 | -4.15 | -3.50 |  | -5.00 | -4.15 | -3.50 |
| Ningxia | 2.71 | 3.63 | 4.34 |  | 2.71 | 3.63 | 4.34 |  | 2.71 | 3.63 | 4.34 |
| Xinjiang | -1.42 | -0.54 | 0.14 |  | -1.42 | -0.54 | 0.14 |  | -1.42 | -0.54 | 0.14 |

**Table S2-3**

**Assumptions on average annual growth rates of economic growth in China based on moderate scenario (Unit: %).**

|  | 2021-2025 | | |  | 2026-2030 | | |  | 2031-2035 | | |
| --- | --- | --- | --- | --- | --- | --- | --- | --- | --- | --- | --- |
| Province | Best | Middle | Baseline |  | Best | Middle | Baseline |  | Best | Middle | Baseline |
| Beijing | 7.50 | 6.50 | 5.50 |  | 7.50 | 6.50 | 5.50 |  | 7.50 | 6.50 | 5.50 |
| Tianjin | 4.93 | 3.93 | 2.93 |  | 4.93 | 3.93 | 2.93 |  | 4.93 | 3.93 | 2.93 |
| Hebei | 7.63 | 6.63 | 5.63 |  | 7.63 | 6.63 | 5.63 |  | 7.63 | 6.63 | 5.63 |
| Shanxi | 7.63 | 6.63 | 5.63 |  | 7.63 | 6.63 | 5.63 |  | 7.63 | 6.63 | 5.63 |
| Inner Mongolia | 5.80 | 4.80 | 3.80 |  | 5.80 | 4.80 | 3.80 |  | 5.80 | 4.80 | 3.80 |
| Liaoning | 6.10 | 5.10 | 4.10 |  | 6.10 | 5.10 | 4.10 |  | 6.10 | 5.10 | 4.10 |
| Jilin | 5.23 | 4.23 | 3.23 |  | 5.23 | 4.23 | 3.23 |  | 5.23 | 4.23 | 3.23 |
| Heilongjiang | 6.03 | 5.03 | 4.03 |  | 6.03 | 5.03 | 4.03 |  | 6.03 | 5.03 | 4.03 |
| Shanghai | 7.57 | 6.57 | 5.57 |  | 7.57 | 6.57 | 5.57 |  | 7.57 | 6.57 | 5.57 |
| Jiangsu | 7.67 | 6.67 | 5.67 |  | 7.67 | 6.67 | 5.67 |  | 7.67 | 6.67 | 5.67 |
| Zhejiang | 8.23 | 7.23 | 6.23 |  | 8.23 | 7.23 | 6.23 |  | 8.23 | 7.23 | 6.23 |
| Anhui | 9.00 | 8.00 | 7.00 |  | 9.00 | 8.00 | 7.00 |  | 9.00 | 8.00 | 7.00 |
| Fujian | 9.00 | 8.00 | 7.00 |  | 9.00 | 8.00 | 7.00 |  | 9.00 | 8.00 | 7.00 |
| Jiangxi | 9.50 | 8.50 | 7.50 |  | 9.50 | 8.50 | 7.50 |  | 9.50 | 8.50 | 7.50 |
| Shandong | 7.40 | 6.40 | 5.40 |  | 7.40 | 6.40 | 5.40 |  | 7.40 | 6.40 | 5.40 |
| Henan | 8.47 | 7.47 | 6.47 |  | 8.47 | 7.47 | 6.47 |  | 8.47 | 7.47 | 6.47 |
| Hubei | 8.70 | 7.70 | 6.70 |  | 8.70 | 7.70 | 6.70 |  | 8.70 | 7.70 | 6.70 |
| Hunan | 8.80 | 7.80 | 6.80 |  | 8.80 | 7.80 | 6.80 |  | 8.80 | 7.80 | 6.80 |
| Guangdong | 7.83 | 6.83 | 5.83 |  | 7.83 | 6.83 | 5.83 |  | 7.83 | 6.83 | 5.83 |
| Guangxi | 7.63 | 6.63 | 5.63 |  | 7.63 | 6.63 | 5.63 |  | 7.63 | 6.63 | 5.63 |
| Hainan | 7.20 | 6.20 | 5.20 |  | 7.20 | 6.20 | 5.20 |  | 7.20 | 6.20 | 5.20 |
| Chongqing | 8.19 | 7.19 | 6.19 |  | 8.19 | 7.19 | 6.19 |  | 8.19 | 7.19 | 6.19 |
| Sichuan | 8.87 | 7.87 | 6.87 |  | 8.87 | 7.87 | 6.87 |  | 8.87 | 7.87 | 6.87 |
| Guizhou | 10.20 | 9.20 | 8.20 |  | 10.20 | 9.20 | 8.20 |  | 10.20 | 9.20 | 8.20 |
| Yunnan | 9.83 | 8.83 | 7.83 |  | 9.83 | 8.83 | 7.83 |  | 9.83 | 8.83 | 7.83 |
| Shaanxi | 8.36 | 7.36 | 6.36 |  | 8.36 | 7.36 | 6.36 |  | 8.36 | 7.36 | 6.36 |
| Gansu | 6.29 | 5.29 | 4.29 |  | 6.29 | 5.29 | 4.29 |  | 6.29 | 5.29 | 4.29 |
| Qinghai | 7.90 | 6.90 | 5.90 |  | 7.90 | 6.90 | 5.90 |  | 7.90 | 6.90 | 5.90 |
| Ningxia | 8.03 | 7.03 | 6.03 |  | 8.03 | 7.03 | 6.03 |  | 8.03 | 7.03 | 6.03 |
| Xinjiang | 7.63 | 6.63 | 5.63 |  | 7.63 | 6.63 | 5.63 |  | 7.63 | 6.63 | 5.63 |

**Table S2-4**

Assumptions on average annual growth rates of carbon intensity in China based on moderate scenario (Unit: %).

|  | 2021-2025 | | |  | 2026-2030 | | |  | 2031-2035 | | |
| --- | --- | --- | --- | --- | --- | --- | --- | --- | --- | --- | --- |
| Province | Best | Middle | Baseline |  | Best | Middle | Baseline |  | Best | Middle | Baseline |
| Beijing | -8.51 | -8.15 | -7.87 |  | -8.51 | -8.15 | -7.87 |  | -8.51 | -8.15 | -7.87 |
| Tianjin | -7.80 | -7.43 | -7.15 |  | -7.80 | -7.43 | -7.15 |  | -7.80 | -7.43 | -7.15 |
| Hebei | -5.77 | -5.39 | -5.10 |  | -5.77 | -5.39 | -5.10 |  | -5.77 | -5.39 | -5.10 |
| Shanxi | -4.26 | -3.88 | -3.59 |  | -4.26 | -3.88 | -3.59 |  | -4.26 | -3.88 | -3.59 |
| Inner Mongolia | -4.80 | -4.43 | -4.14 |  | -4.80 | -4.43 | -4.14 |  | -4.80 | -4.43 | -4.14 |
| Liaoning | -4.20 | -3.82 | -3.53 |  | -4.20 | -3.82 | -3.53 |  | -4.20 | -3.82 | -3.53 |
| Jilin | -7.63 | -7.26 | -6.98 |  | -7.63 | -7.26 | -6.98 |  | -7.63 | -7.26 | -6.98 |
| Heilongjiang | -5.06 | -4.68 | -4.39 |  | -5.06 | -4.68 | -4.39 |  | -5.06 | -4.68 | -4.39 |
| Shanghai | -7.23 | -6.87 | -6.58 |  | -7.23 | -6.87 | -6.58 |  | -7.23 | -6.87 | -6.58 |
| Jiangsu | -5.01 | -4.63 | -4.34 |  | -5.01 | -4.63 | -4.34 |  | -5.01 | -4.63 | -4.34 |
| Zhejiang | -6.27 | -5.89 | -5.61 |  | -6.27 | -5.89 | -5.61 |  | -6.27 | -5.89 | -5.61 |
| Anhui | -4.98 | -4.60 | -4.31 |  | -4.98 | -4.60 | -4.31 |  | -4.98 | -4.60 | -4.31 |
| Fujian | -7.42 | -7.05 | -6.77 |  | -7.42 | -7.05 | -6.77 |  | -7.42 | -7.05 | -6.77 |
| Jiangxi | -4.13 | -3.75 | -3.45 |  | -4.13 | -3.75 | -3.45 |  | -4.13 | -3.75 | -3.45 |
| Shandong | -6.23 | -5.86 | -5.58 |  | -6.23 | -5.86 | -5.58 |  | -6.23 | -5.86 | -5.58 |
| Henan | -7.94 | -7.57 | -7.29 |  | -7.94 | -7.57 | -7.29 |  | -7.94 | -7.57 | -7.29 |
| Hubei | -9.12 | -8.76 | -8.48 |  | -9.12 | -8.76 | -8.48 |  | -9.12 | -8.76 | -8.48 |
| Hunan | -6.46 | -6.09 | -5.80 |  | -6.46 | -6.09 | -5.80 |  | -6.46 | -6.09 | -5.80 |
| Guangdong | -5.87 | -5.49 | -5.21 |  | -5.87 | -5.49 | -5.21 |  | -5.87 | -5.49 | -5.21 |
| Guangxi | -5.05 | -4.68 | -4.39 |  | -5.05 | -4.68 | -4.39 |  | -5.05 | -4.68 | -4.39 |
| Hainan | -3.96 | -3.57 | -3.28 |  | -3.96 | -3.57 | -3.28 |  | -3.96 | -3.57 | -3.28 |
| Chongqing | -8.43 | -8.07 | -7.79 |  | -8.43 | -8.07 | -7.79 |  | -8.43 | -8.07 | -7.79 |
| Sichuan | -7.37 | -7.00 | -6.72 |  | -7.37 | -7.00 | -6.72 |  | -7.37 | -7.00 | -6.72 |
| Guizhou | -6.66 | -6.29 | -6.01 |  | -6.66 | -6.29 | -6.01 |  | -6.66 | -6.29 | -6.01 |
| Yunnan | -8.33 | -7.97 | -7.69 |  | -8.33 | -7.97 | -7.69 |  | -8.33 | -7.97 | -7.69 |
| Shaanxi | -6.41 | -6.04 | -5.75 |  | -6.41 | -6.04 | -5.75 |  | -6.41 | -6.04 | -5.75 |
| Gansu | -5.81 | -5.44 | -5.15 |  | -5.81 | -5.44 | -5.15 |  | -5.81 | -5.44 | -5.15 |
| Qinghai | -2.66 | -2.27 | -1.97 |  | -2.66 | -2.27 | -1.97 |  | -2.66 | -2.27 | -1.97 |
| Ningxia | -3.59 | -3.21 | -2.92 |  | -3.59 | -3.21 | -2.92 |  | -3.59 | -3.21 | -2.92 |
| Xinjiang | 0.81 | 1.21 | 1.51 |  | 0.81 | 1.21 | 1.51 |  | 0.81 | 1.21 | 1.51 |

**Table S2-5**

Assumptions on average annual growth rates of economic growth in China based on advanced scenario (Unit: %).

|  | 2021-2025 | | |  | 2026-2030 | | |  | 2031-2035 | | |
| --- | --- | --- | --- | --- | --- | --- | --- | --- | --- | --- | --- |
| Province | Best | Middle | Baseline |  | Best | Middle | Baseline |  | Best | Middle | Baseline |
| Beijing | 7.46 | 6.46 | 5.46 |  | 7.46 | 6.46 | 5.46 |  | 7.46 | 6.46 | 5.46 |
| Tianjin | 8.57 | 7.57 | 6.57 |  | 8.57 | 7.57 | 6.57 |  | 8.57 | 7.57 | 6.57 |
| Hebei | 7.66 | 6.66 | 5.66 |  | 7.66 | 6.66 | 5.66 |  | 7.66 | 6.66 | 5.66 |
| Shanxi | 6.94 | 5.94 | 4.94 |  | 6.94 | 5.94 | 4.94 |  | 6.94 | 5.94 | 4.94 |
| Inner Mongolia | 7.62 | 6.62 | 5.62 |  | 7.62 | 6.62 | 5.62 |  | 7.62 | 6.62 | 5.62 |
| Liaoning | 5.62 | 4.62 | 3.62 |  | 5.62 | 4.62 | 3.62 |  | 5.62 | 4.62 | 3.62 |
| Jilin | 7.08 | 6.08 | 5.08 |  | 7.08 | 6.08 | 5.08 |  | 7.08 | 6.08 | 5.08 |
| Heilongjiang | 6.85 | 5.85 | 4.85 |  | 6.85 | 5.85 | 4.85 |  | 6.85 | 5.85 | 4.85 |
| Shanghai | 7.43 | 6.43 | 5.43 |  | 7.43 | 6.43 | 5.43 |  | 7.43 | 6.43 | 5.43 |
| Jiangsu | 8.42 | 7.42 | 6.42 |  | 8.42 | 7.42 | 6.42 |  | 8.42 | 7.42 | 6.42 |
| Zhejiang | 8.03 | 7.03 | 6.03 |  | 8.03 | 7.03 | 6.03 |  | 8.03 | 7.03 | 6.03 |
| Anhui | 9.35 | 8.35 | 7.35 |  | 9.35 | 8.35 | 7.35 |  | 9.35 | 8.35 | 7.35 |
| Fujian | 9.41 | 8.41 | 7.41 |  | 9.41 | 8.41 | 7.41 |  | 9.41 | 8.41 | 7.41 |
| Jiangxi | 9.50 | 8.50 | 7.50 |  | 9.50 | 8.50 | 7.50 |  | 9.50 | 8.50 | 7.50 |
| Shandong | 8.22 | 7.22 | 6.22 |  | 8.22 | 7.22 | 6.22 |  | 8.22 | 7.22 | 6.22 |
| Henan | 8.66 | 7.66 | 6.66 |  | 8.66 | 7.66 | 6.66 |  | 8.66 | 7.66 | 6.66 |
| Hubei | 9.14 | 8.14 | 7.14 |  | 9.14 | 8.14 | 7.14 |  | 9.14 | 8.14 | 7.14 |
| Hunan | 9.10 | 8.10 | 7.10 |  | 9.10 | 8.10 | 7.10 |  | 9.10 | 8.10 | 7.10 |
| Guangdong | 7.96 | 6.96 | 5.96 |  | 7.96 | 6.96 | 5.96 |  | 7.96 | 6.96 | 5.96 |
| Guangxi | 8.48 | 7.48 | 6.48 |  | 8.48 | 7.48 | 6.48 |  | 8.48 | 7.48 | 6.48 |
| Hainan | 8.06 | 7.06 | 6.06 |  | 8.06 | 7.06 | 6.06 |  | 8.06 | 7.06 | 6.06 |
| Chongqing | 10.10 | 9.10 | 8.10 |  | 10.10 | 9.10 | 8.10 |  | 10.10 | 9.10 | 8.10 |
| Sichuan | 9.05 | 8.05 | 7.05 |  | 9.05 | 8.05 | 7.05 |  | 9.05 | 8.05 | 7.05 |
| Guizhou | 10.73 | 9.73 | 8.73 |  | 10.73 | 9.73 | 8.73 |  | 10.73 | 9.73 | 8.73 |
| Yunnan | 9.78 | 8.78 | 7.78 |  | 9.78 | 8.78 | 7.78 |  | 9.78 | 8.78 | 7.78 |
| Shaanxi | 9.13 | 8.13 | 7.13 |  | 9.13 | 8.13 | 7.13 |  | 9.13 | 8.13 | 7.13 |
| Gansu | 8.31 | 7.31 | 6.31 |  | 8.31 | 7.31 | 6.31 |  | 8.31 | 7.31 | 6.31 |
| Qinghai | 8.91 | 7.91 | 6.91 |  | 8.91 | 7.91 | 6.91 |  | 8.91 | 7.91 | 6.91 |
| Ningxia | 8.62 | 7.62 | 6.62 |  | 8.62 | 7.62 | 6.62 |  | 8.62 | 7.62 | 6.62 |
| Xinjiang | 8.92 | 7.92 | 6.92 |  | 8.92 | 7.92 | 6.92 |  | 8.92 | 7.92 | 6.92 |

**Table S2-6**

Assumptions on average annual growth rates of carbon intensity in China based on advanced scenario (Unit: %).

|  | 2021-2025 | | |  | 2026-2030 | | |  | | 2031-2035 | | |
| --- | --- | --- | --- | --- | --- | --- | --- | --- | --- | --- | --- | --- |
| Province | Best | Middle | Baseline |  | Best | Middle | Baseline | |  | Best | Middle | Baseline |
| Beijing | -7.84 | -7.44 | -7.04 |  | -7.84 | -7.44 | -7.04 |  | | -7.84 | -7.44 | -7.04 |
| Tianjin | -10.63 | -10.23 | -9.83 |  | -10.63 | -10.23 | -9.83 |  | | -10.63 | -10.23 | -9.83 |
| Hebei | -7.30 | -6.90 | -6.50 |  | -7.30 | -6.90 | -6.50 |  | | -7.30 | -6.90 | -6.50 |
| Shanxi | -6.62 | -6.22 | -5.82 |  | -6.62 | -6.22 | -5.82 |  | | -6.62 | -6.22 | -5.82 |
| Inner Mongolia | -9.17 | -8.77 | -8.37 |  | -9.17 | -8.77 | -8.37 |  | | -9.17 | -8.77 | -8.37 |
| Liaoning | -5.83 | -5.43 | -5.03 |  | -5.83 | -5.43 | -5.03 |  | | -5.83 | -5.43 | -5.03 |
| Jilin | -10.74 | -10.34 | -9.94 |  | -10.74 | -10.34 | -9.94 |  | | -10.74 | -10.34 | -9.94 |
| Heilongjiang | -5.55 | -5.15 | -4.75 |  | -5.55 | -5.15 | -4.75 |  | | -5.55 | -5.15 | -4.75 |
| Shanghai | -8.57 | -8.17 | -7.77 |  | -8.57 | -8.17 | -7.77 |  | | -8.57 | -8.17 | -7.77 |
| Jiangsu | -6.39 | -5.99 | -5.59 |  | -6.39 | -5.99 | -5.59 |  | | -6.39 | -5.99 | -5.59 |
| Zhejiang | -8.01 | -7.61 | -7.21 |  | -8.01 | -7.61 | -7.21 |  | | -8.01 | -7.61 | -7.21 |
| Anhui | -5.21 | -4.81 | -4.41 |  | -5.21 | -4.81 | -4.41 |  | | -5.21 | -4.81 | -4.41 |
| Fujian | -10.38 | -9.98 | -9.58 |  | -10.38 | -9.98 | -9.58 |  | | -10.38 | -9.98 | -9.58 |
| Jiangxi | -3.62 | -3.22 | -2.82 |  | -3.62 | -3.22 | -2.82 |  | | -3.62 | -3.22 | -2.82 |
| Shandong | -8.00 | -7.60 | -7.20 |  | -8.00 | -7.60 | -7.20 |  | | -8.00 | -7.60 | -7.20 |
| Henan | -10.03 | -9.63 | -9.23 |  | -10.03 | -9.63 | -9.23 |  | | -10.03 | -9.63 | -9.23 |
| Hubei | -13.76 | -13.36 | -12.96 |  | -13.76 | -13.36 | -12.96 |  | | -13.76 | -13.36 | -12.96 |
| Hunan | -9.07 | -8.67 | -8.27 |  | -9.07 | -8.67 | -8.27 |  | | -9.07 | -8.67 | -8.27 |
| Guangdong | -8.62 | -8.22 | -7.82 |  | -8.62 | -8.22 | -7.82 |  | | -8.62 | -8.22 | -7.82 |
| Guangxi | -8.42 | -8.02 | -7.62 |  | -8.42 | -8.02 | -7.62 |  | | -8.42 | -8.02 | -7.62 |
| Hainan | -3.99 | -3.59 | -3.19 |  | -3.99 | -3.59 | -3.19 |  | | -3.99 | -3.59 | -3.19 |
| Chongqing | -11.19 | -10.79 | -10.39 |  | -11.19 | -10.79 | -10.39 |  | | -11.19 | -10.79 | -10.39 |
| Sichuan | -7.85 | -7.45 | -7.05 |  | -7.85 | -7.45 | -7.05 |  | | -7.85 | -7.45 | -7.05 |
| Guizhou | -8.73 | -8.33 | -7.93 |  | -8.73 | -8.33 | -7.93 |  | | -8.73 | -8.33 | -7.93 |
| Yunnan | -13.30 | -12.90 | -12.50 |  | -13.30 | -12.90 | -12.50 |  | | -13.30 | -12.90 | -12.50 |
| Shaanxi | -6.86 | -6.46 | -6.06 |  | -6.86 | -6.46 | -6.06 |  | | -6.86 | -6.46 | -6.06 |
| Gansu | -6.51 | -6.11 | -5.71 |  | -6.51 | -6.11 | -5.71 |  | | -6.51 | -6.11 | -5.71 |
| Qinghai | -1.67 | -1.27 | -0.87 |  | -1.67 | -1.27 | -0.87 |  | | -1.67 | -1.27 | -0.87 |
| Ningxia | -8.34 | -7.94 | -7.54 |  | -8.34 | -7.94 | -7.54 |  | | -8.34 | -7.94 | -7.54 |
| Xinjiang | 2.92 | 3.32 | 3.72 |  | 2.92 | 3.32 | 3.72 |  | | 2.92 | 3.32 | 3.72 |

**S3 Gaussian Kuznets curve regression results**


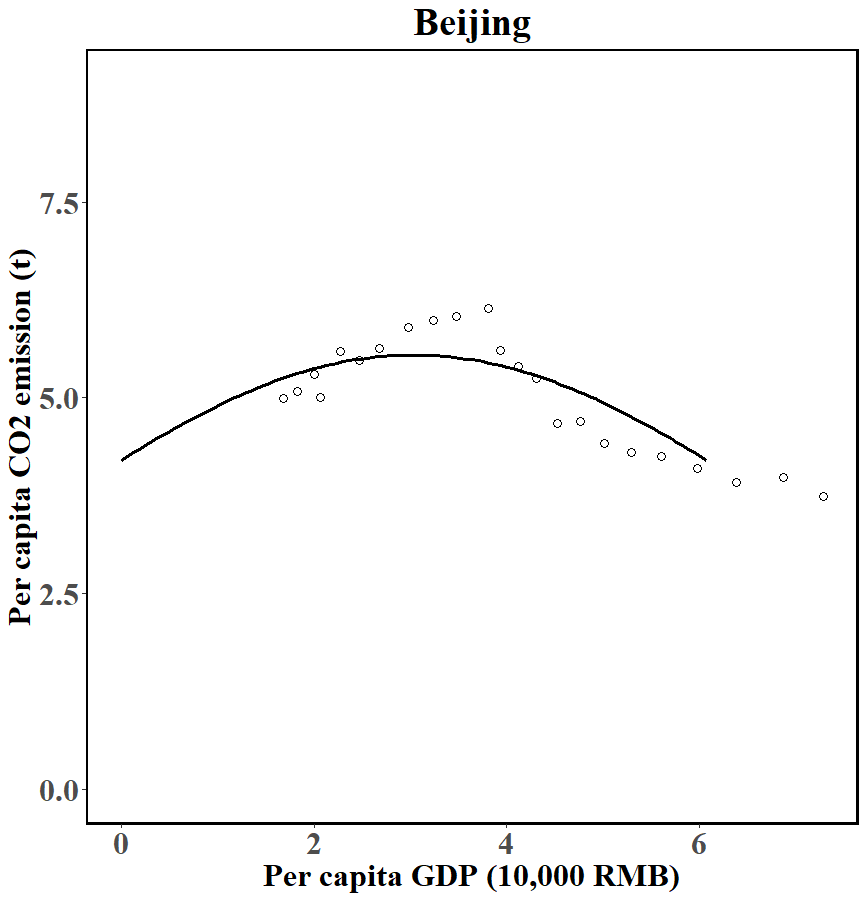

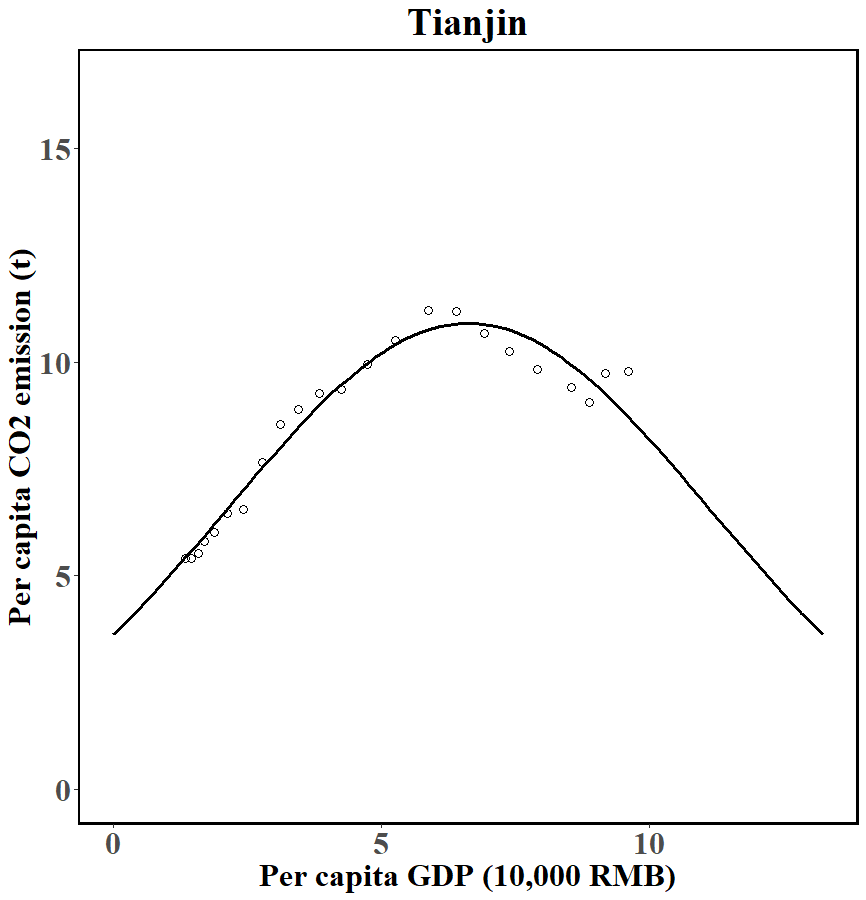

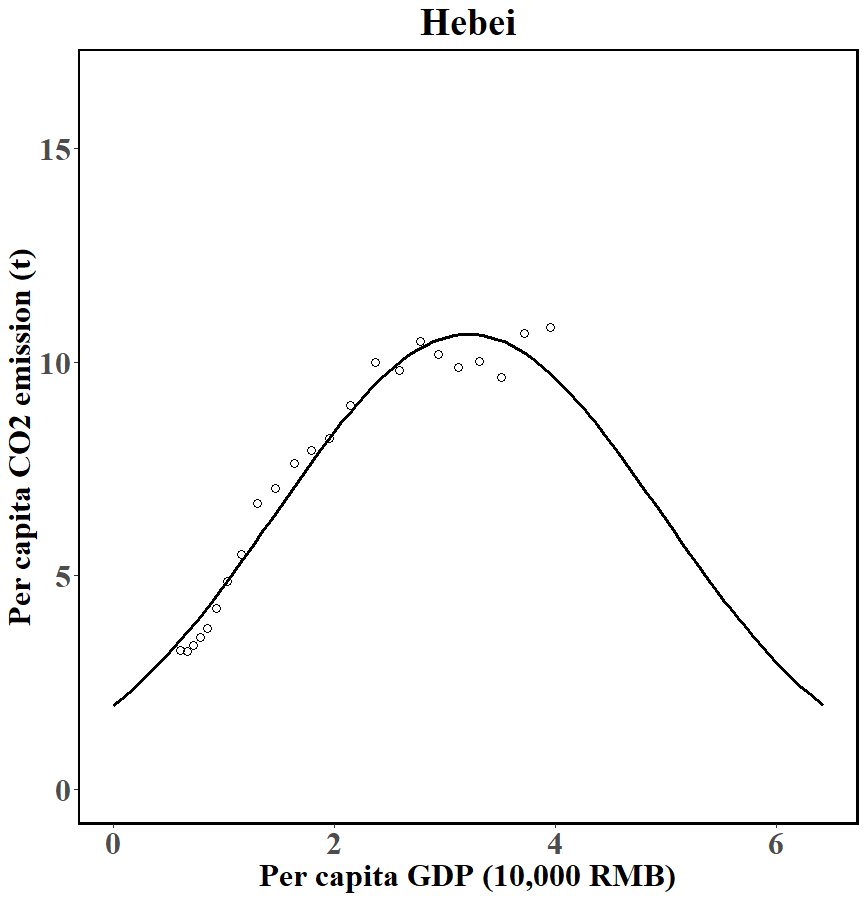

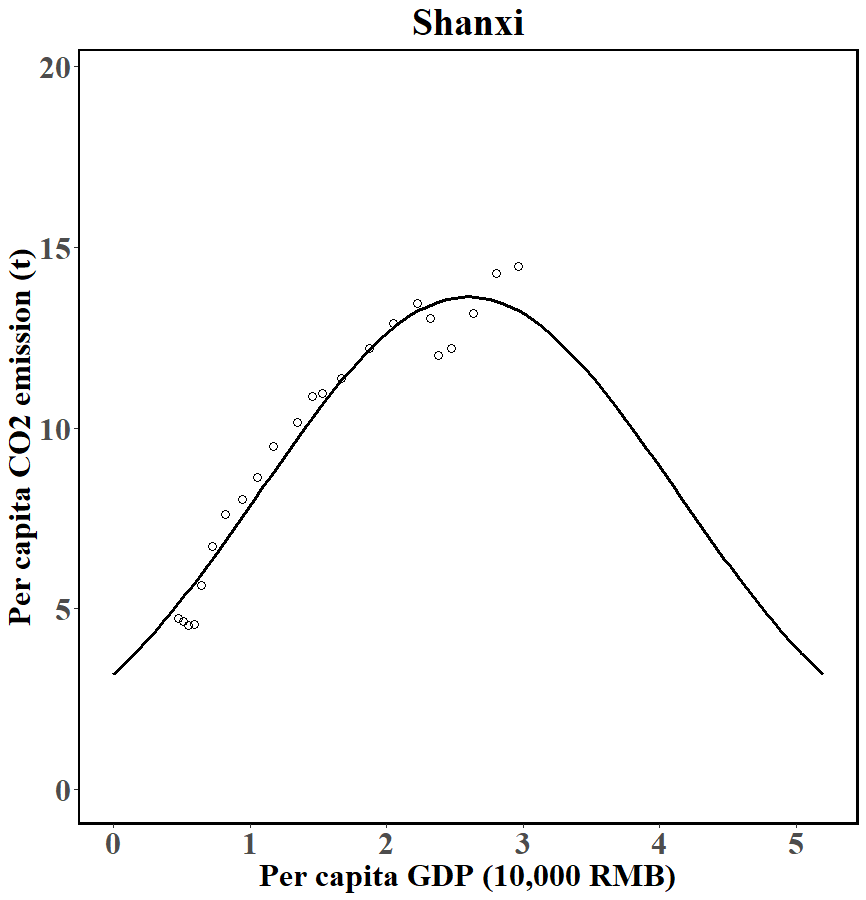

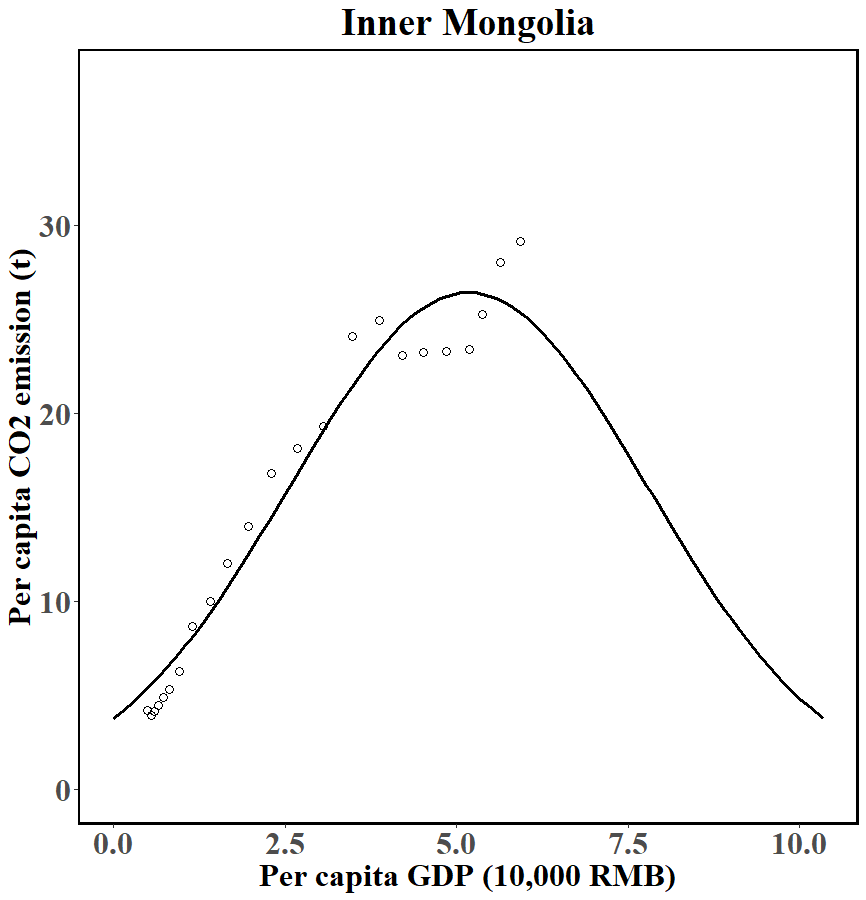


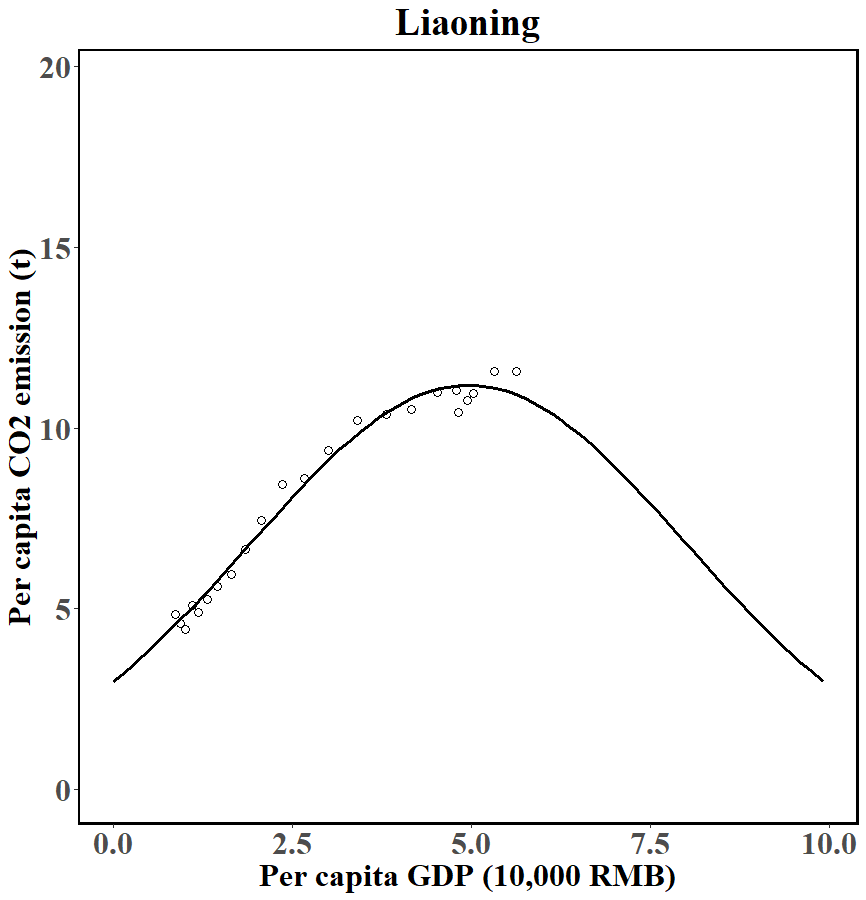

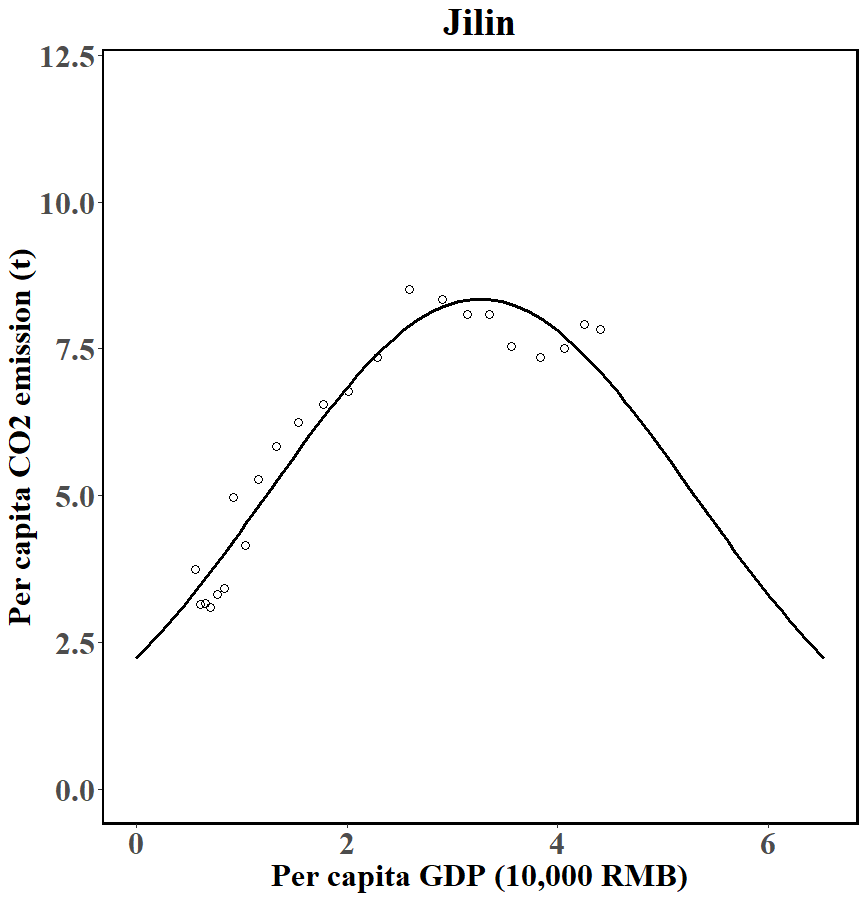

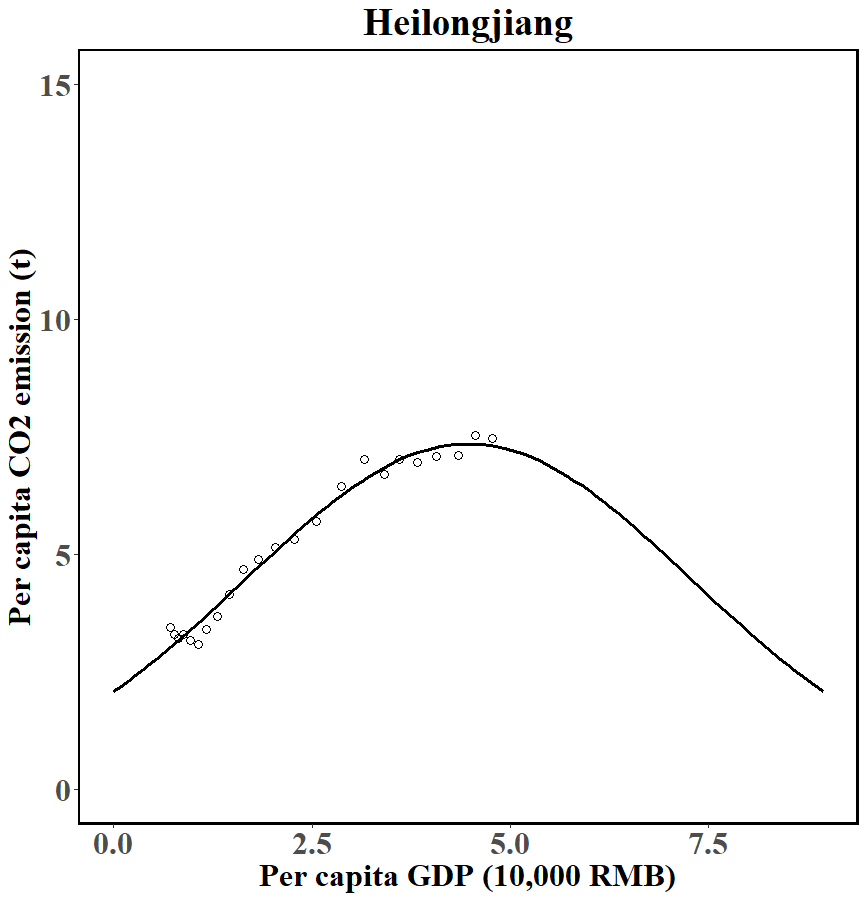

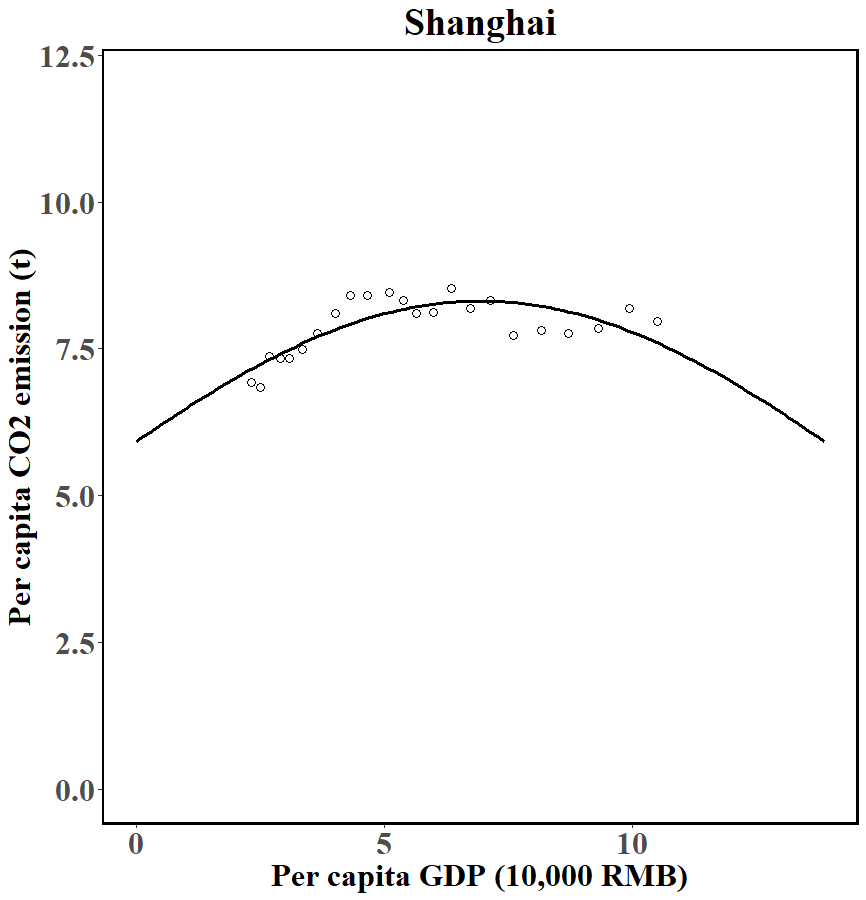

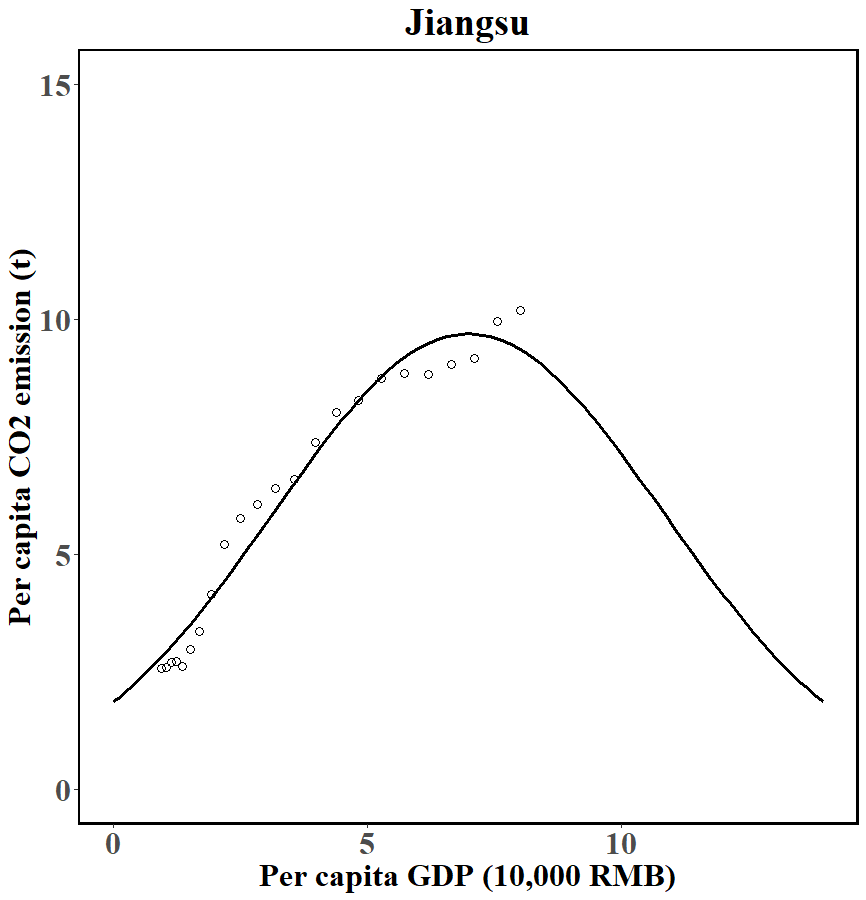


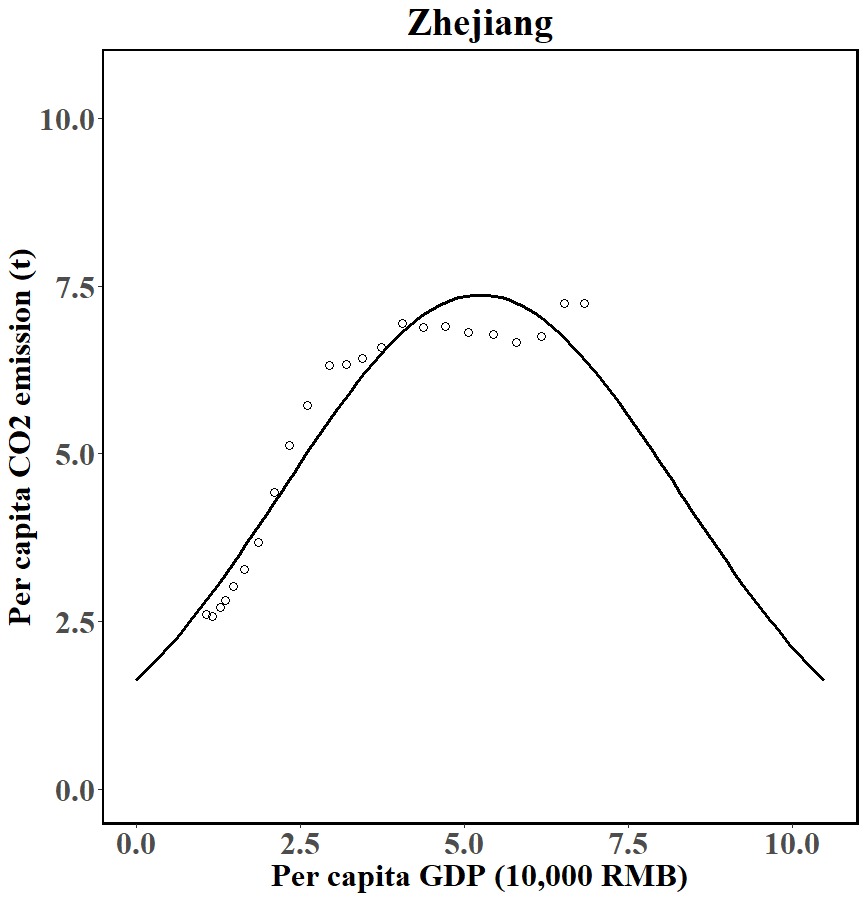

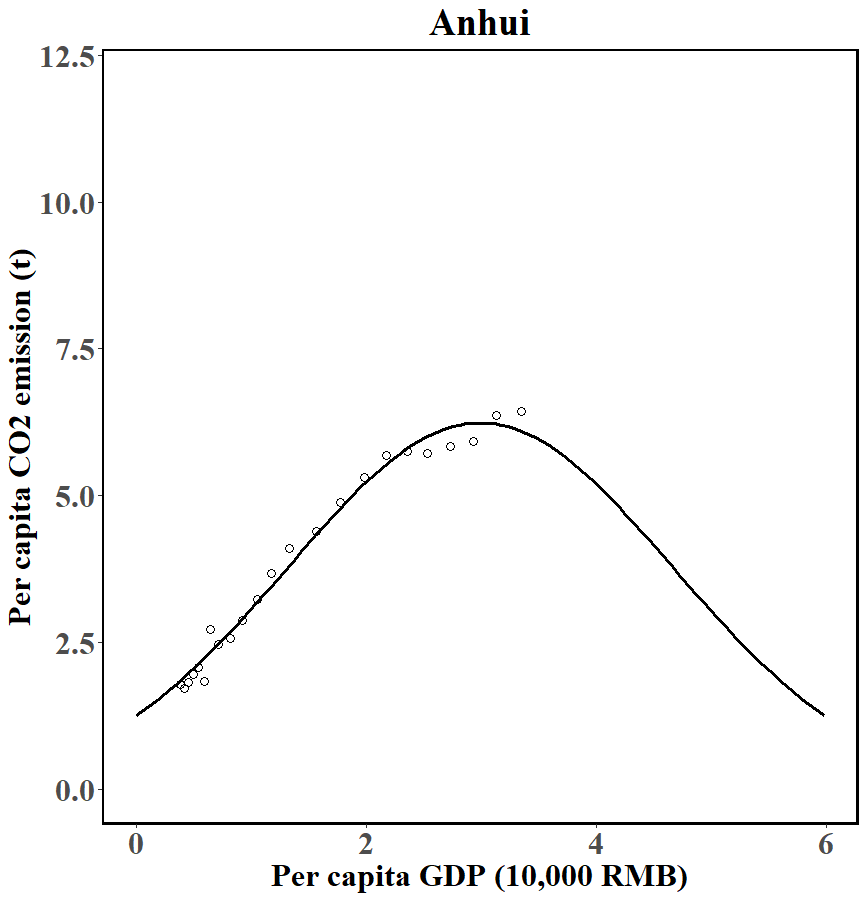

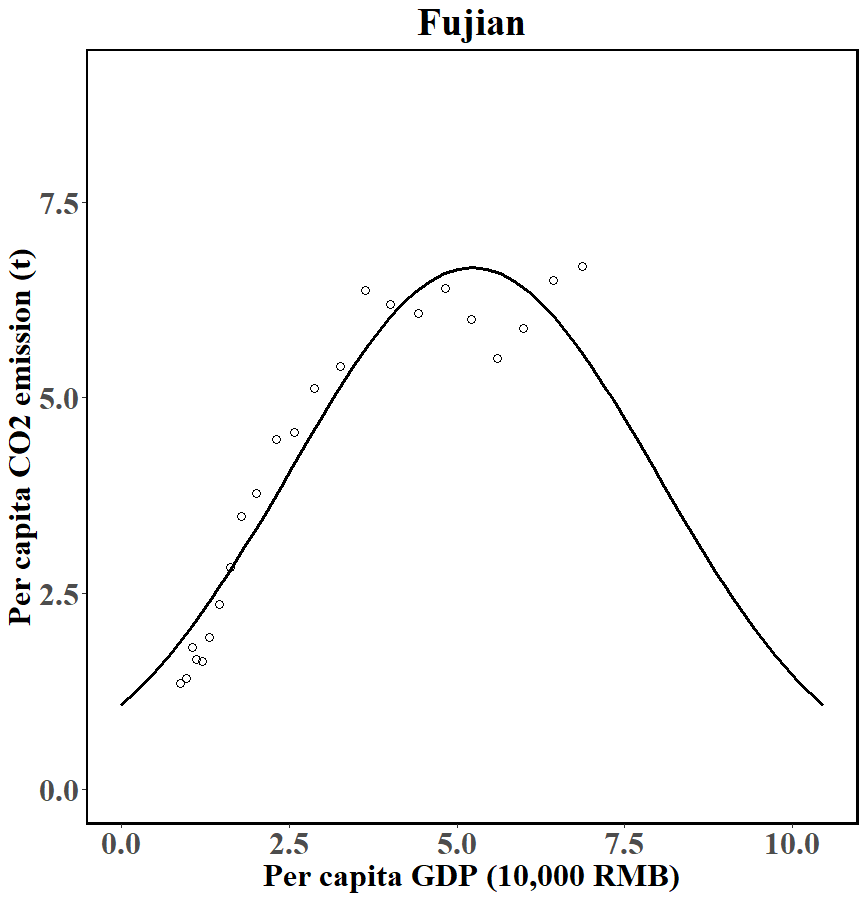

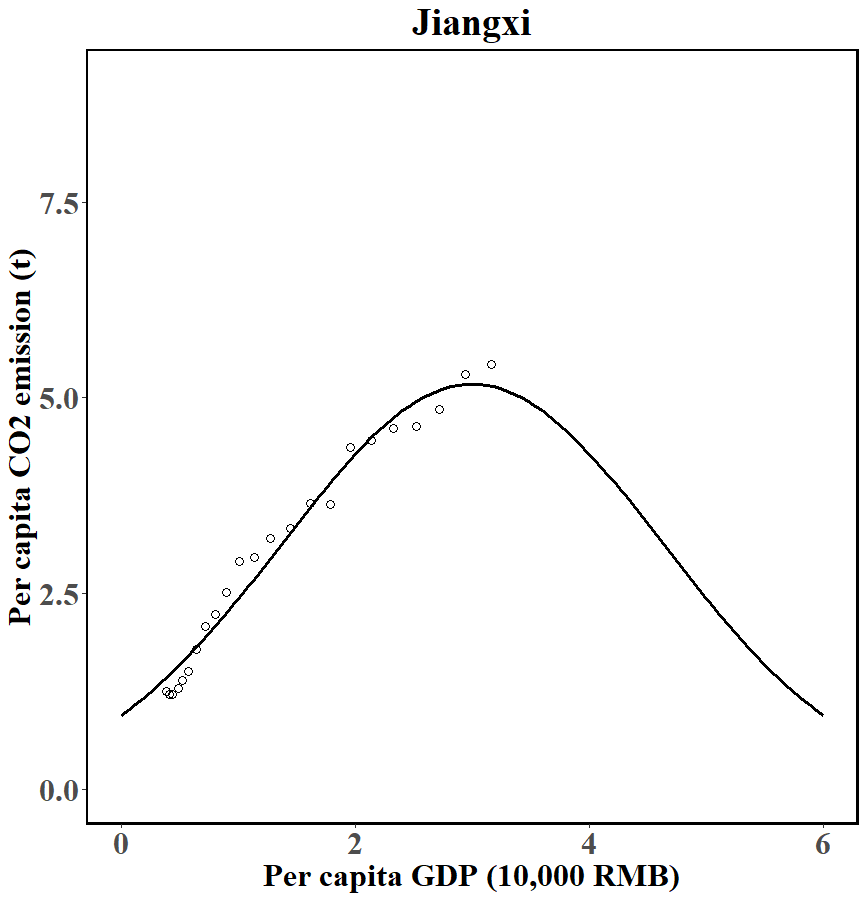

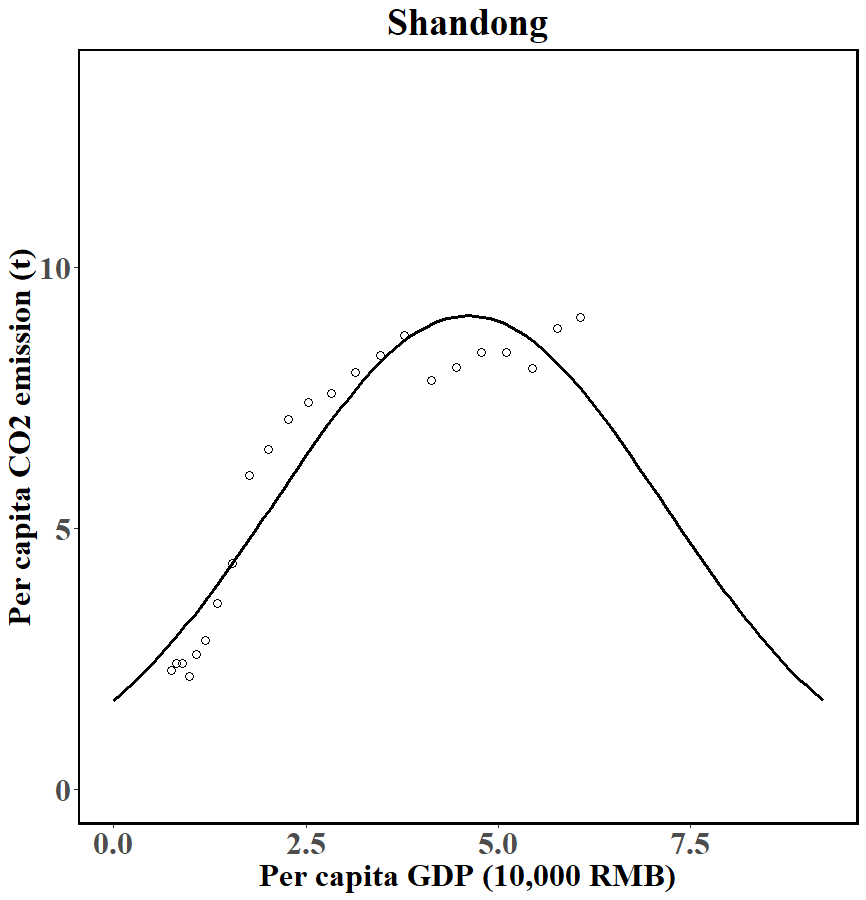


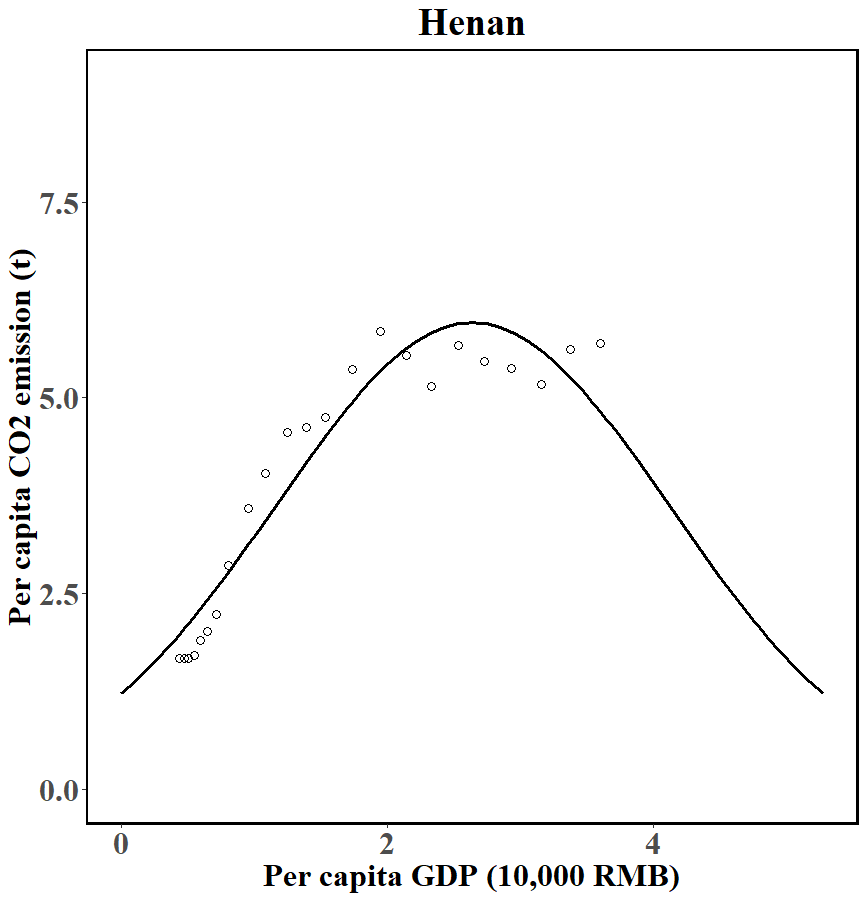

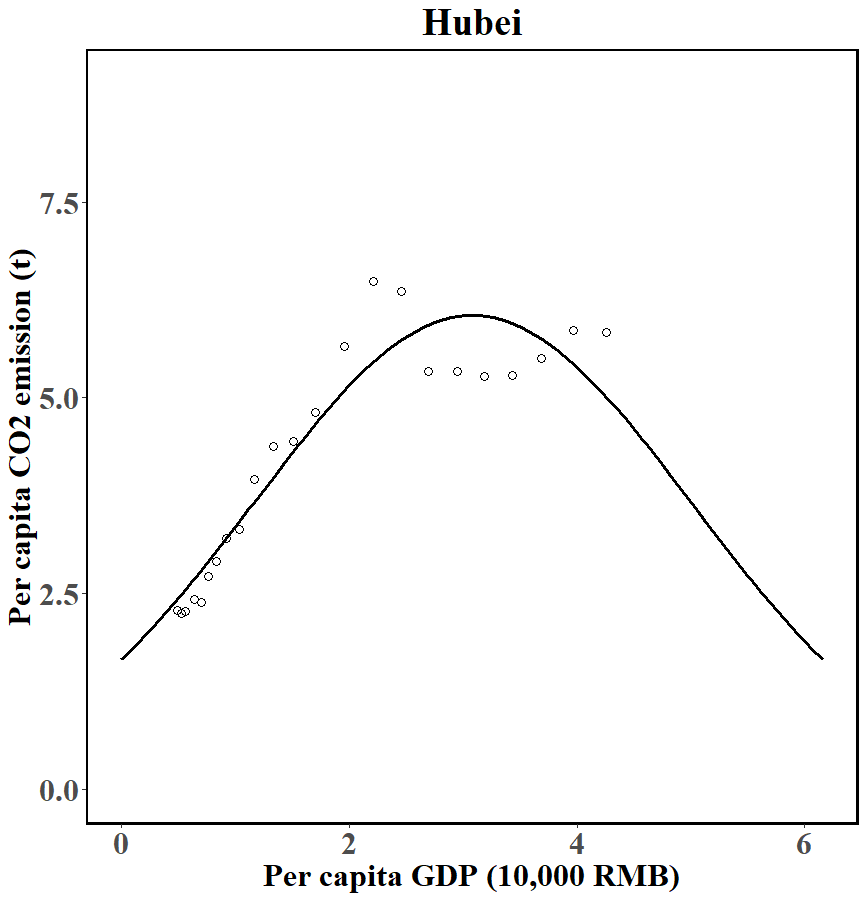

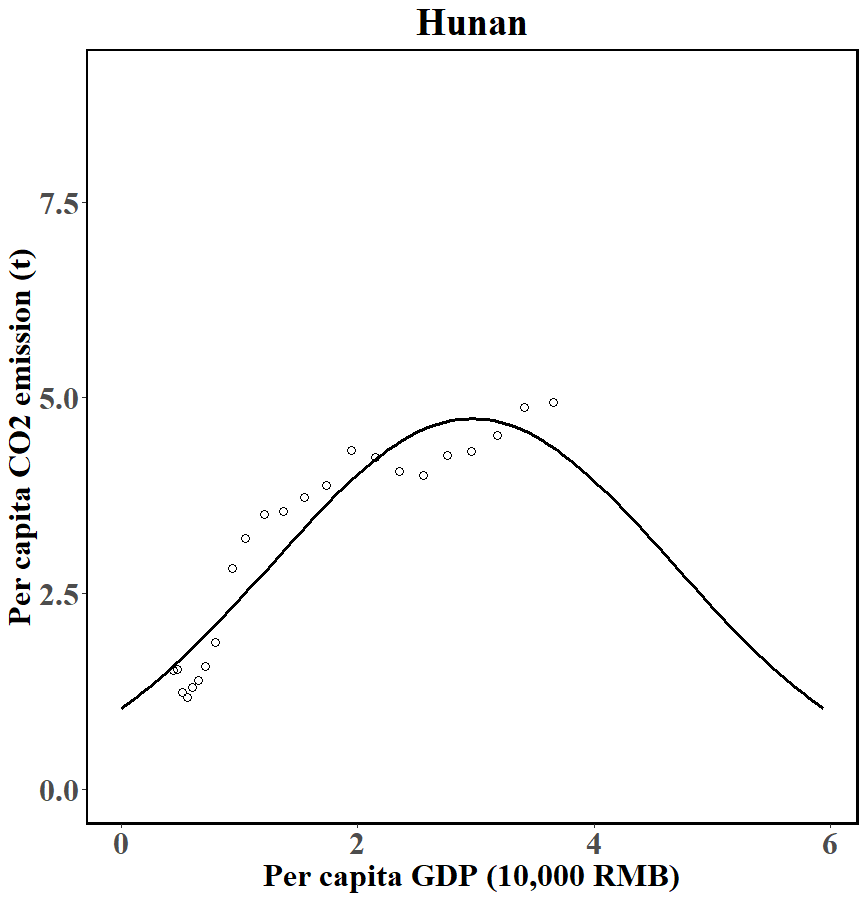

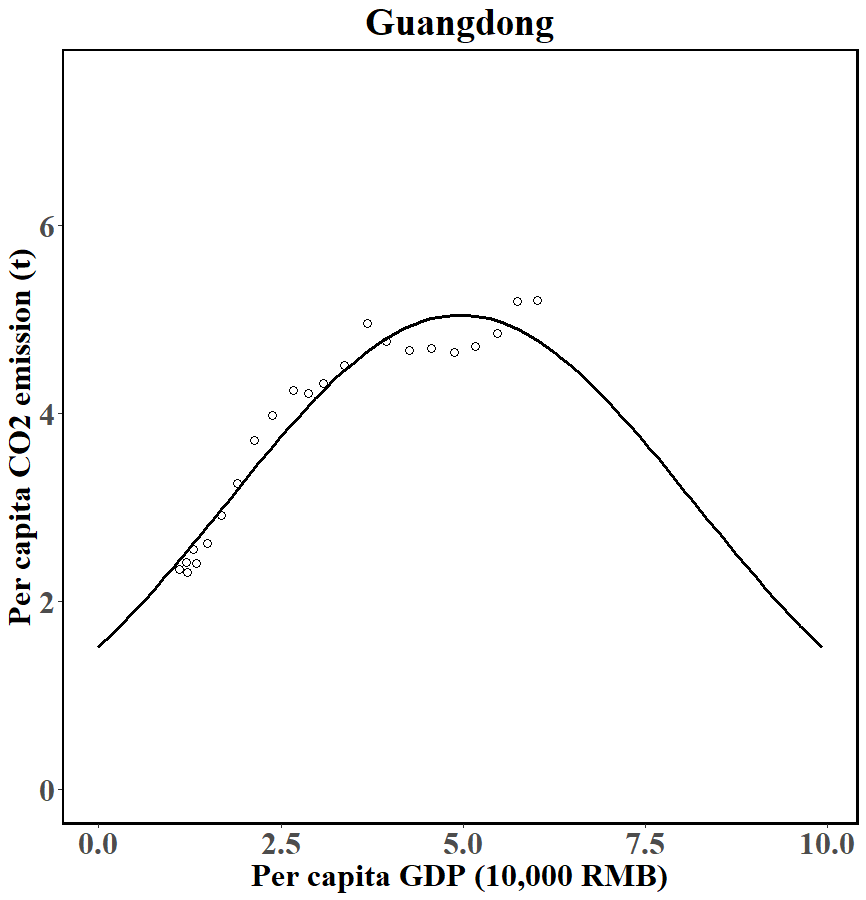

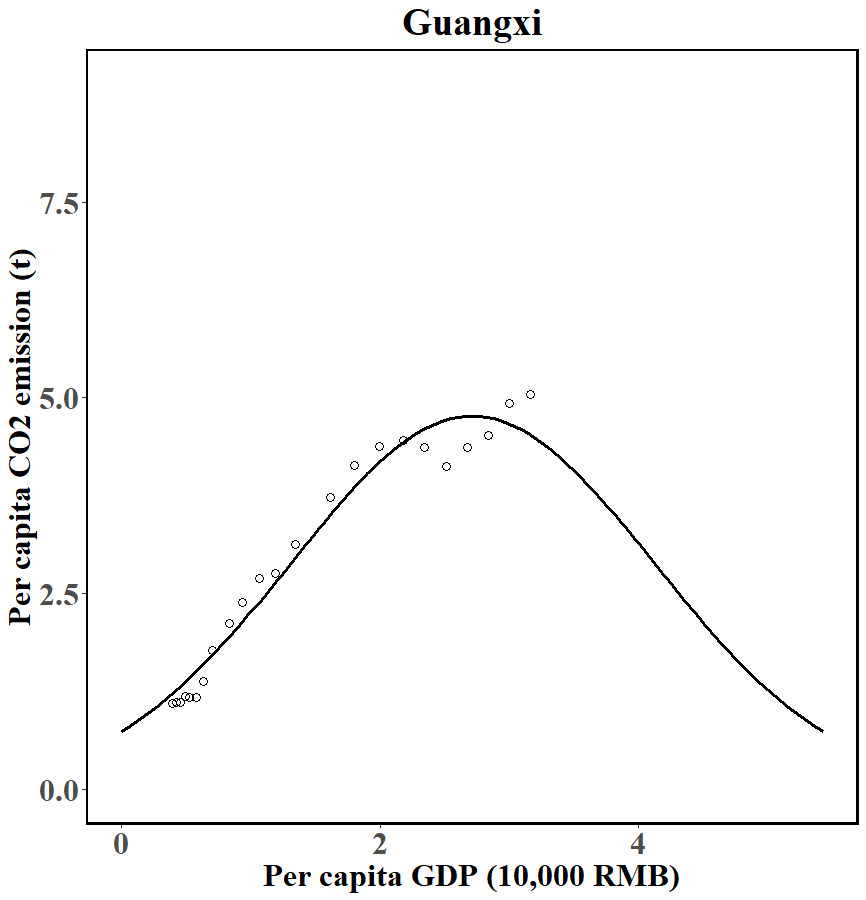


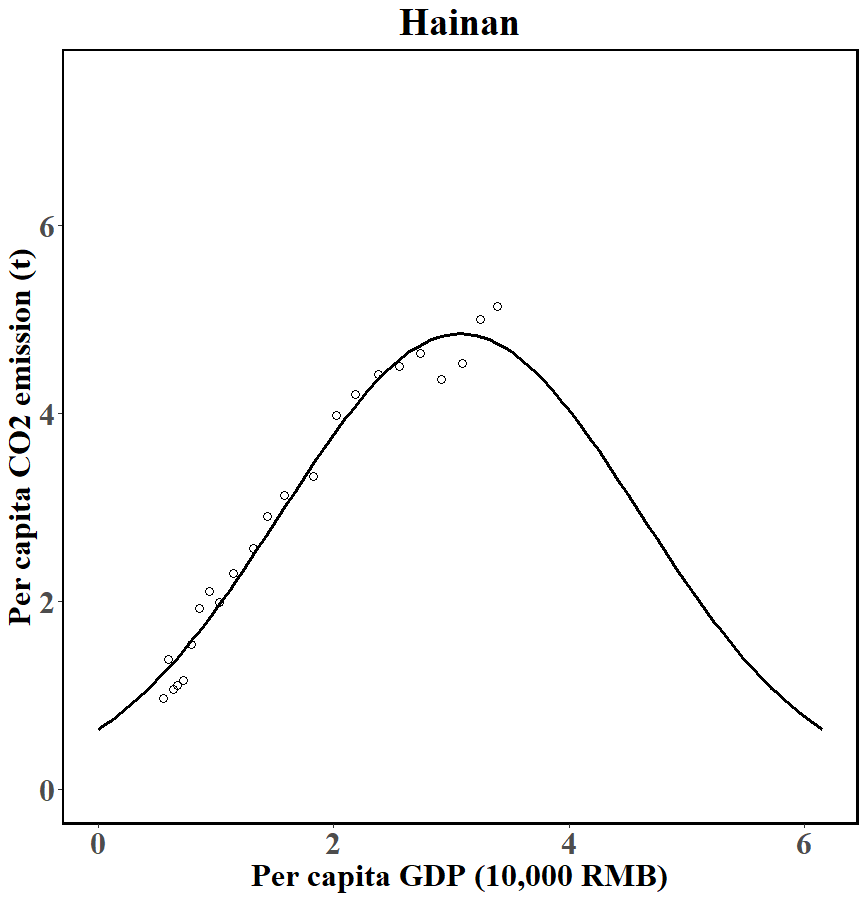

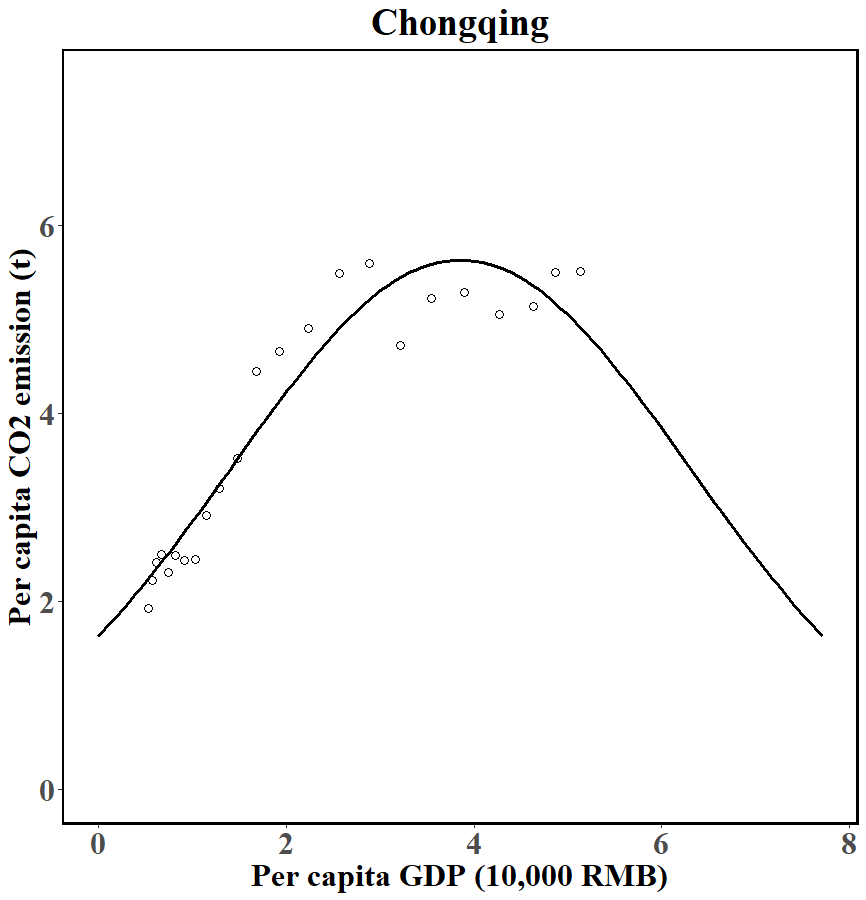

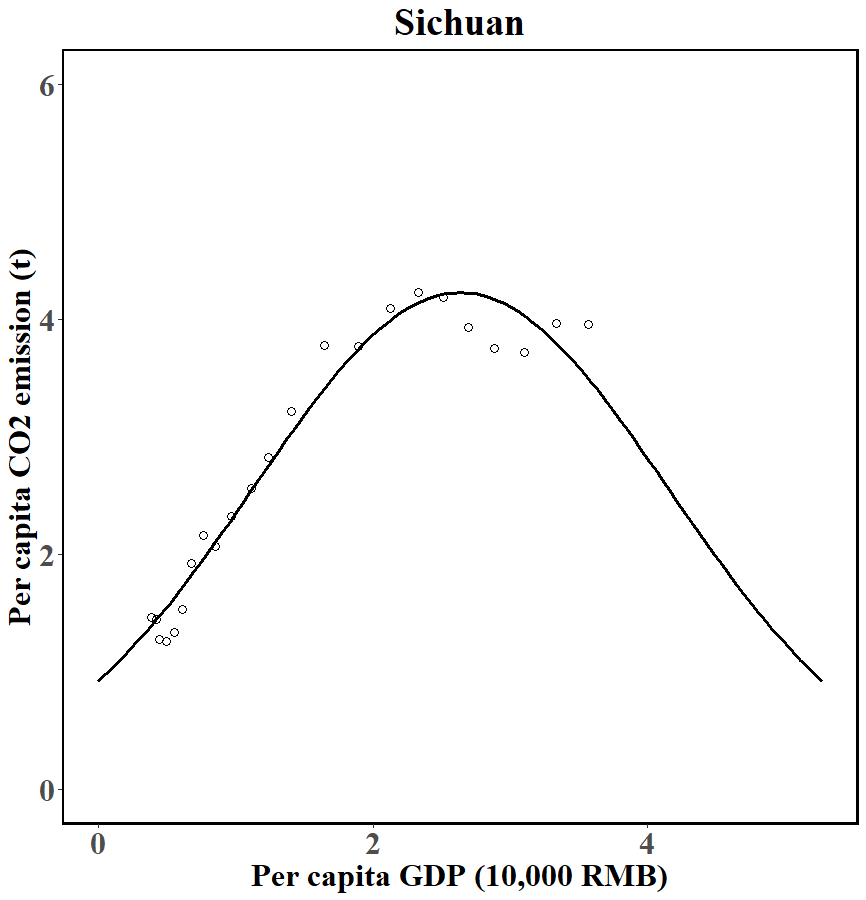

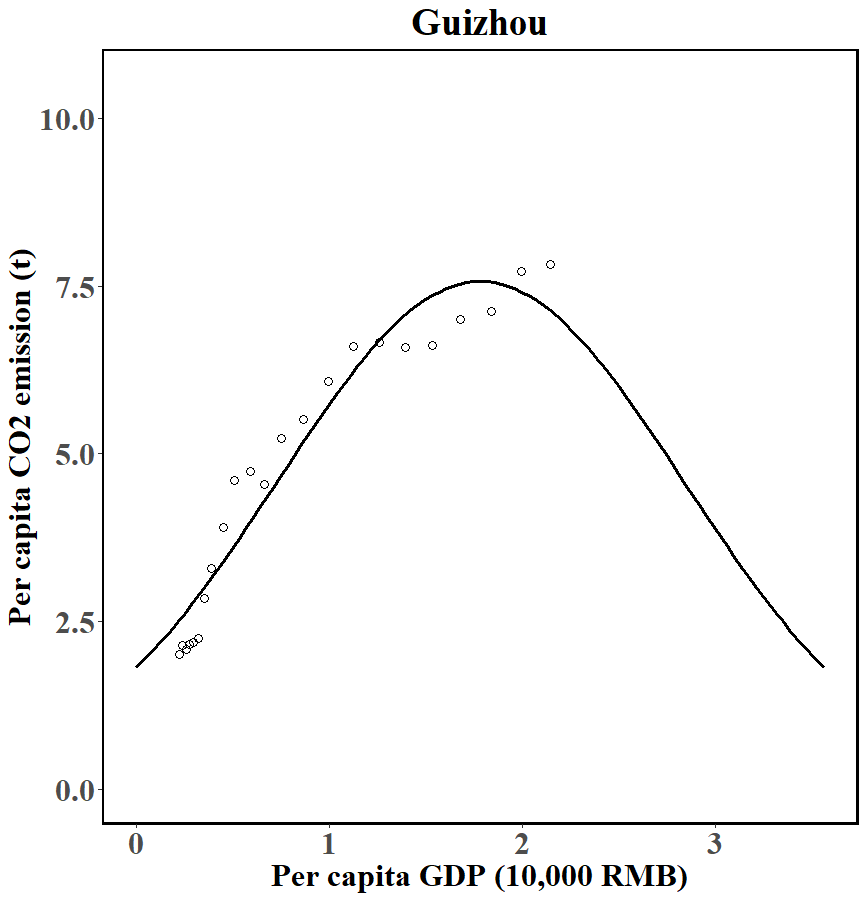

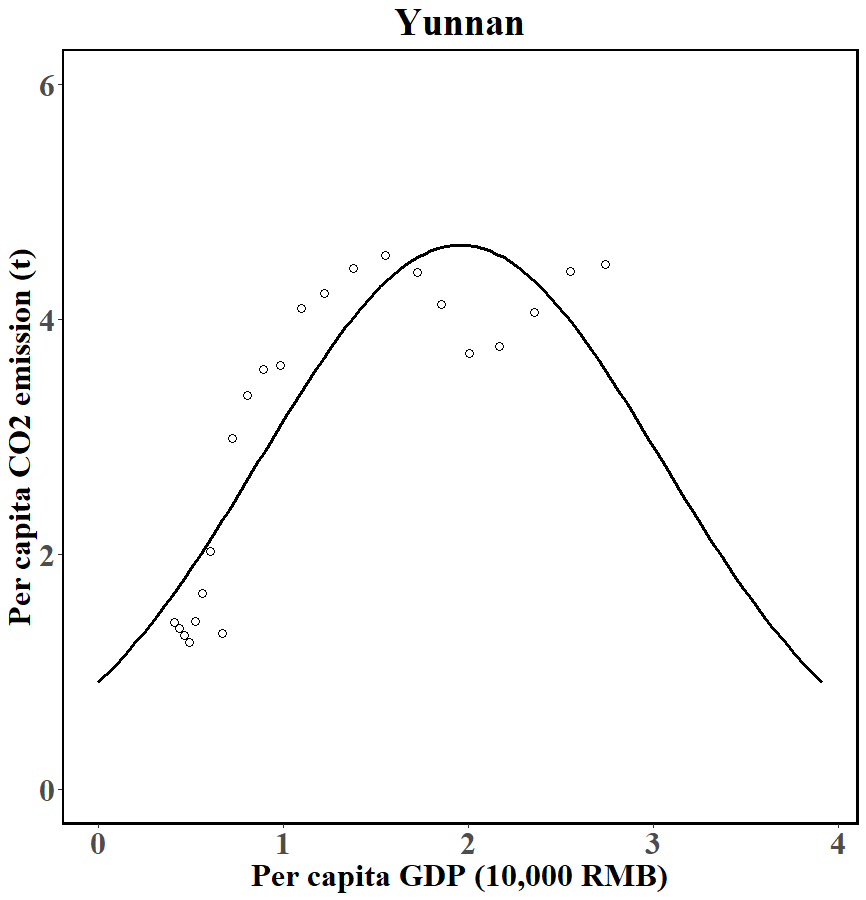


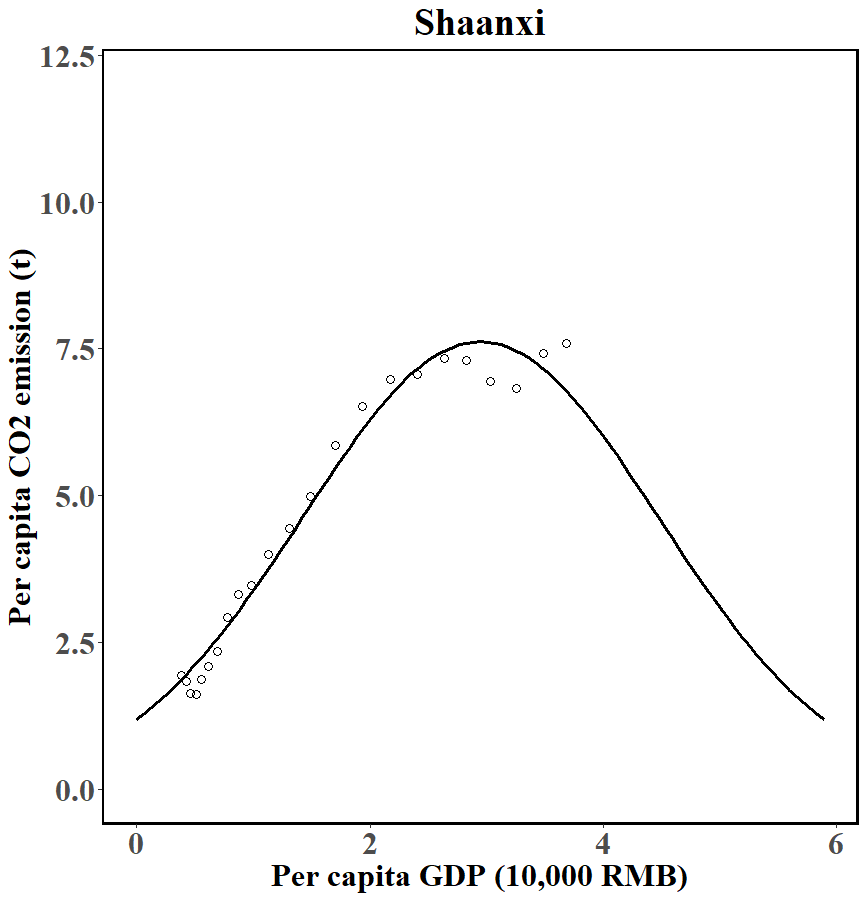

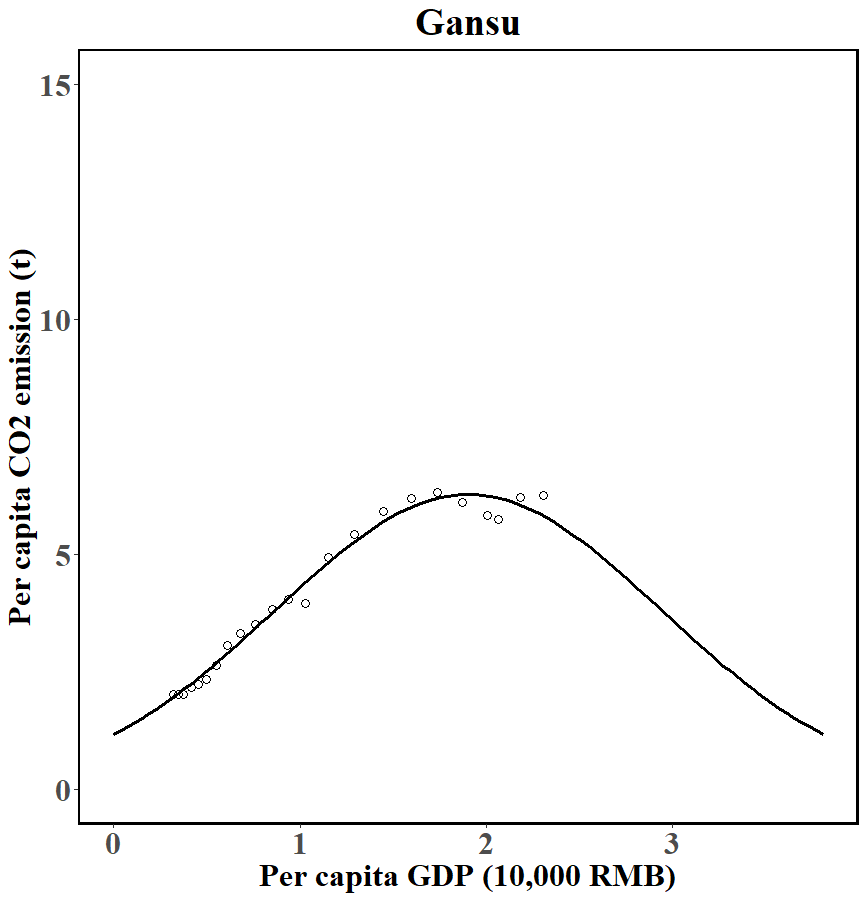

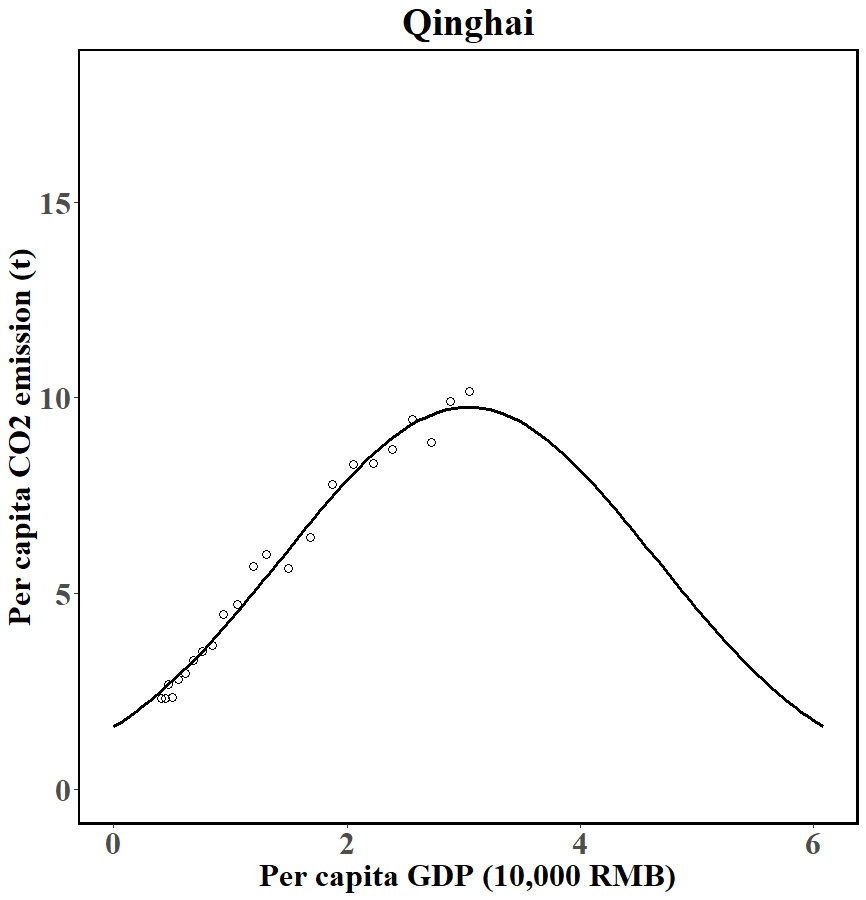

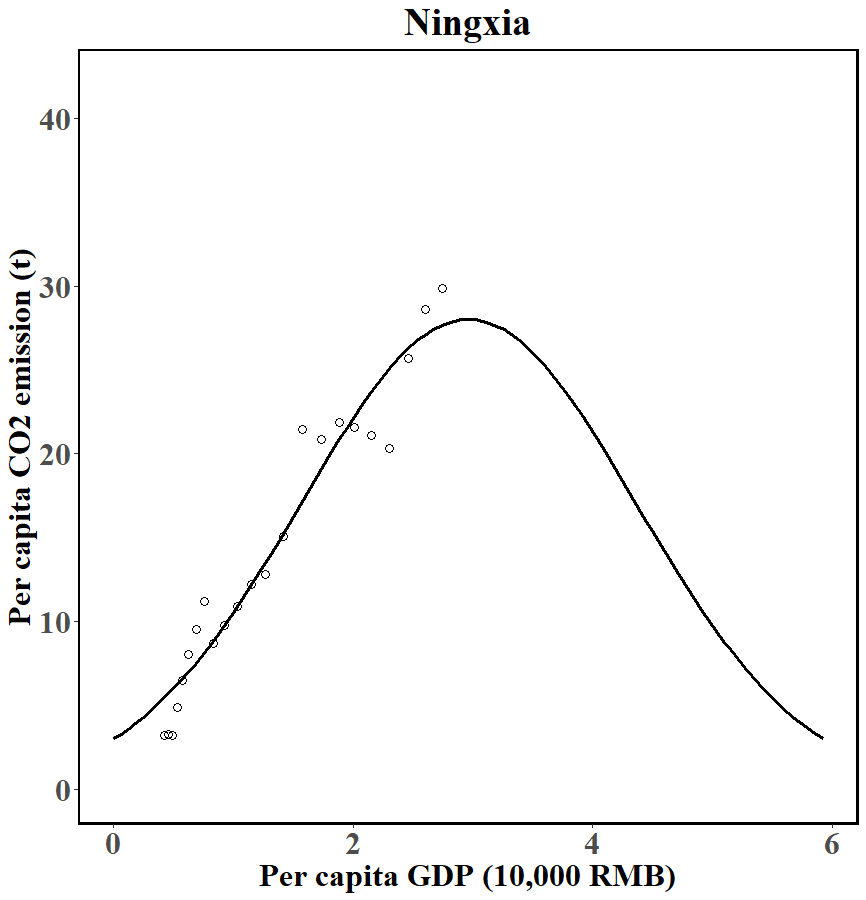

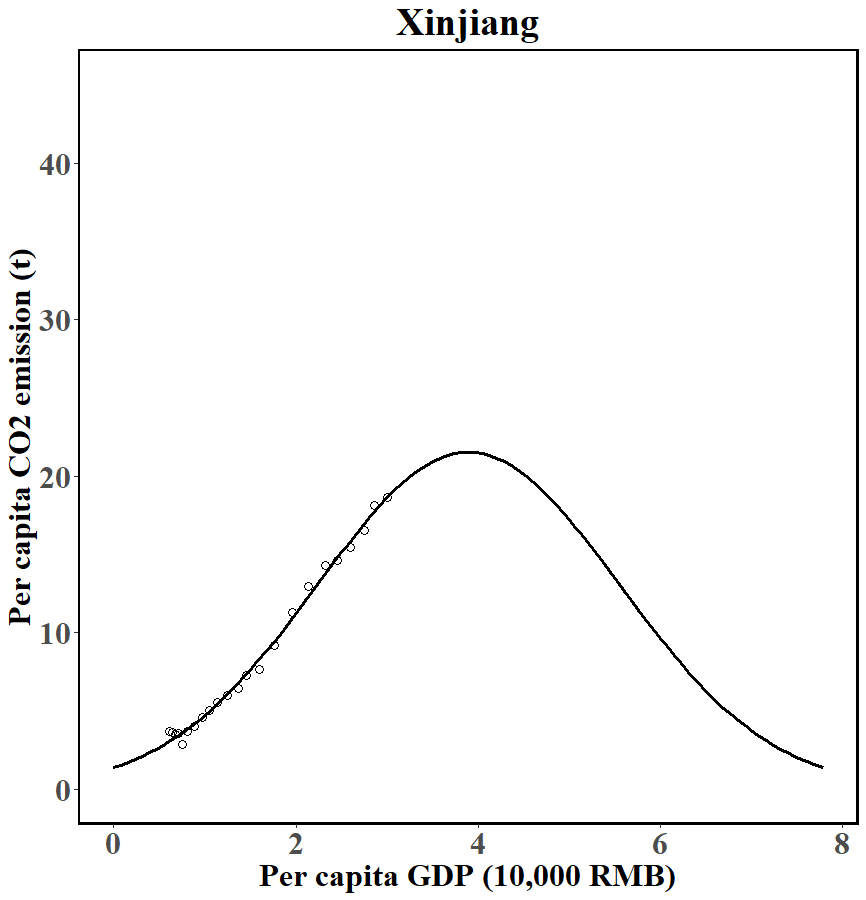


**Figure S3-1** **Gaussian Kuznets curve regression of per capita GDP and CO2 emission per capita for each of the 30 Chinese provinces over 1997-2019. Note that the GDP per capita is based on the constant prices in 1997.**


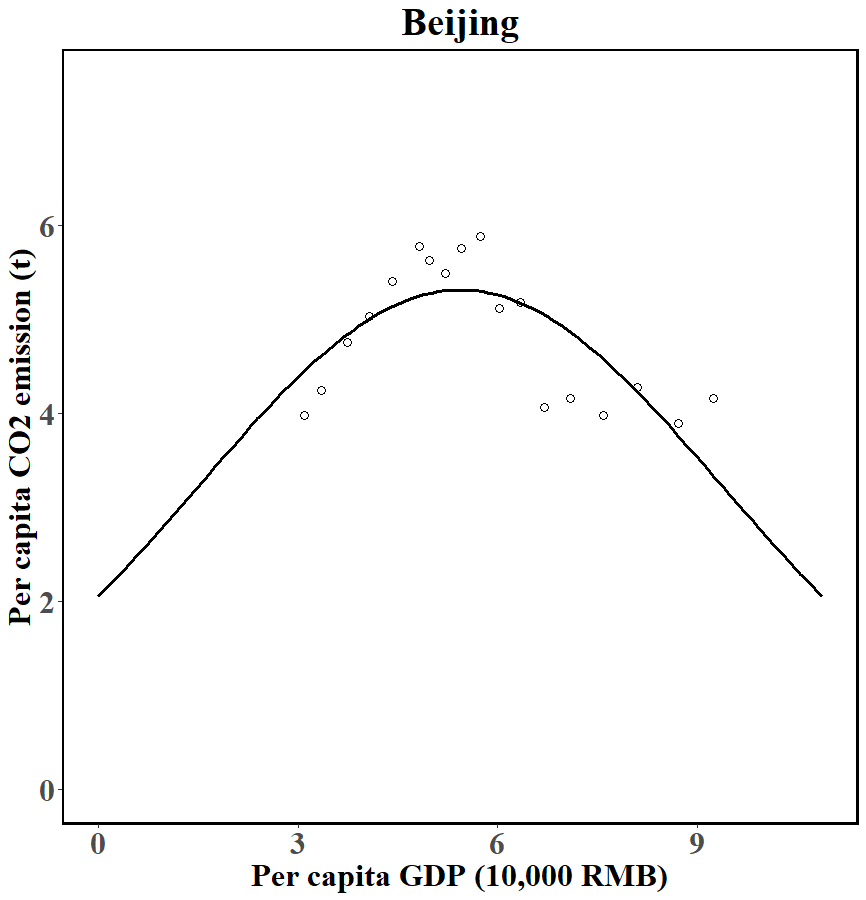

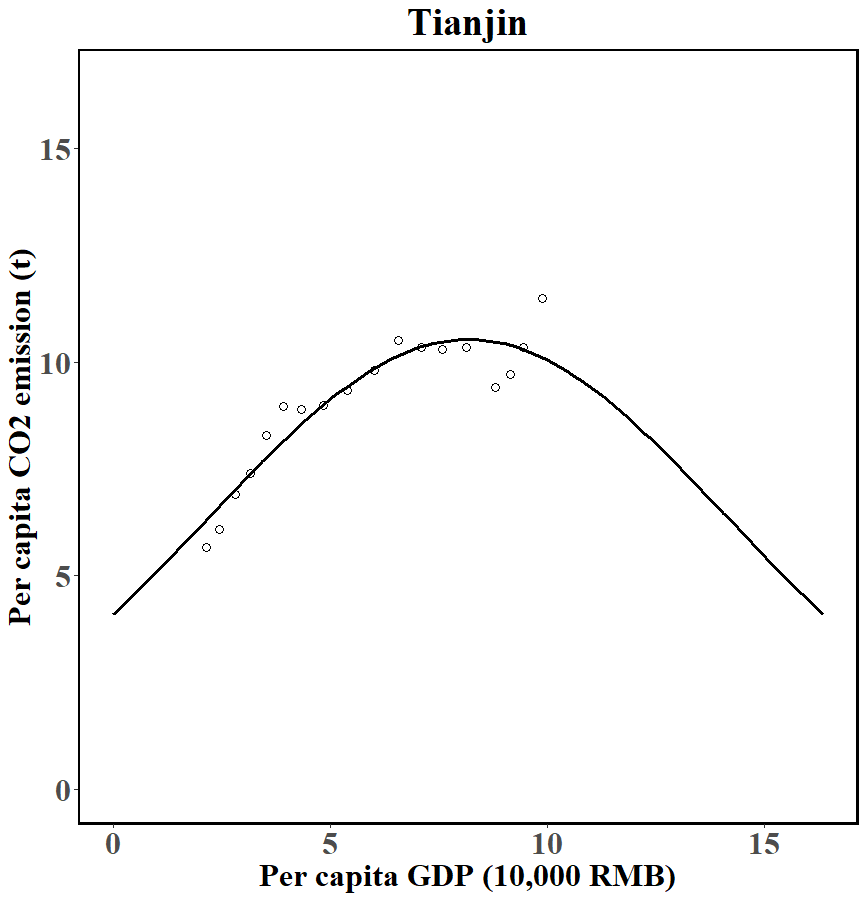

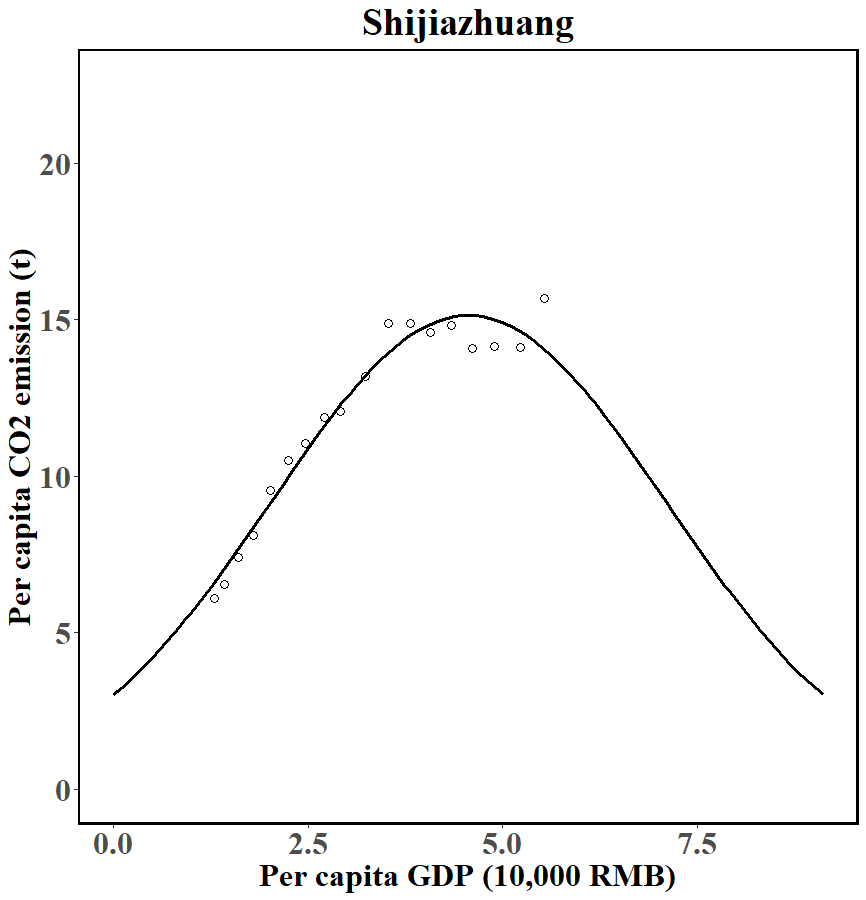

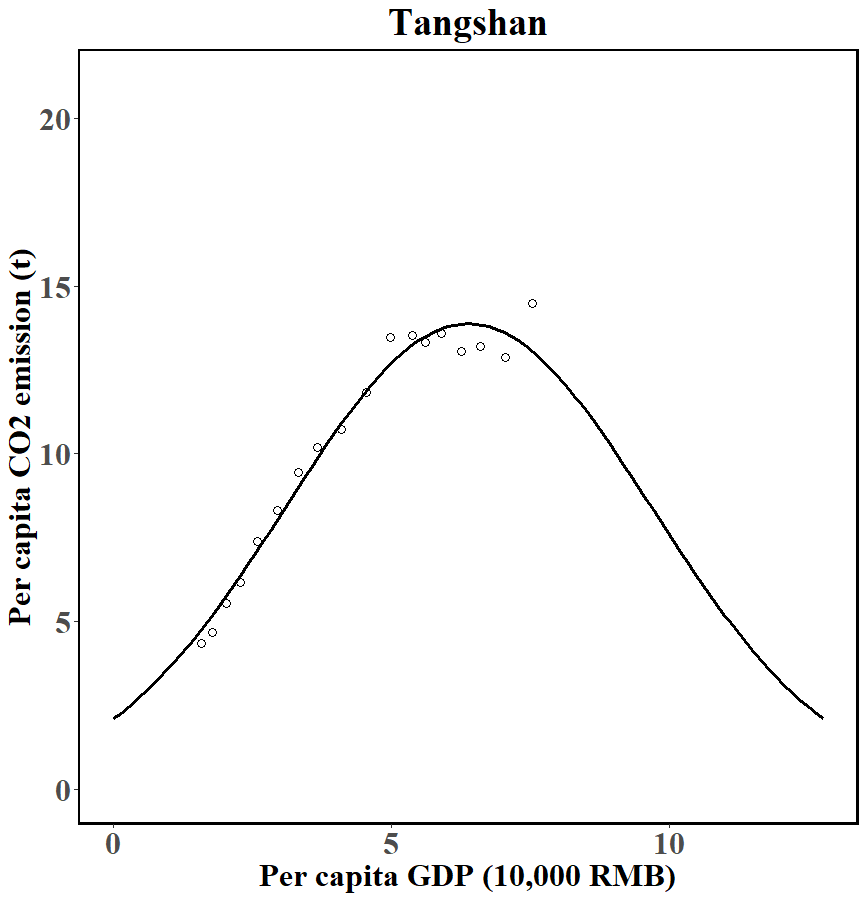

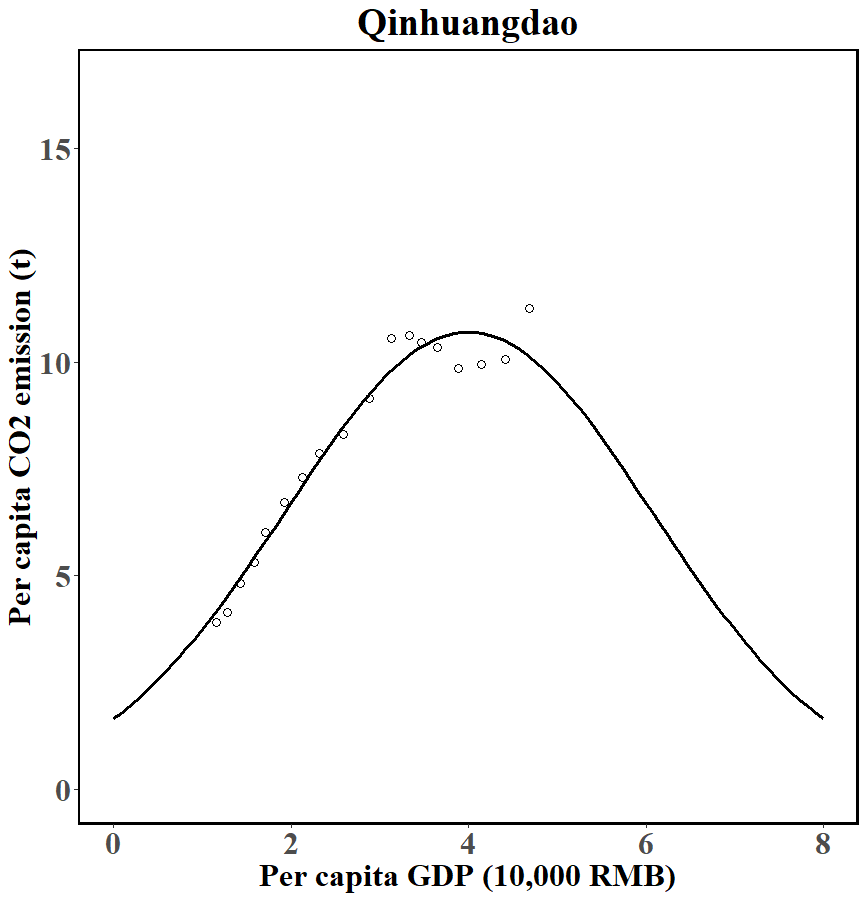

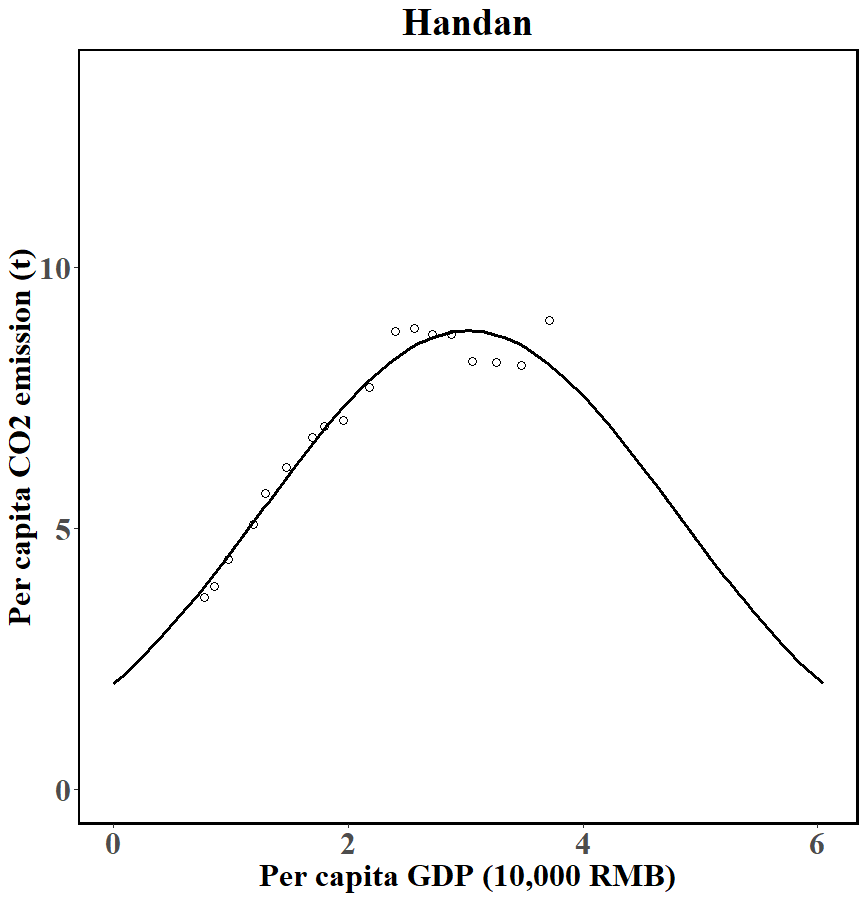

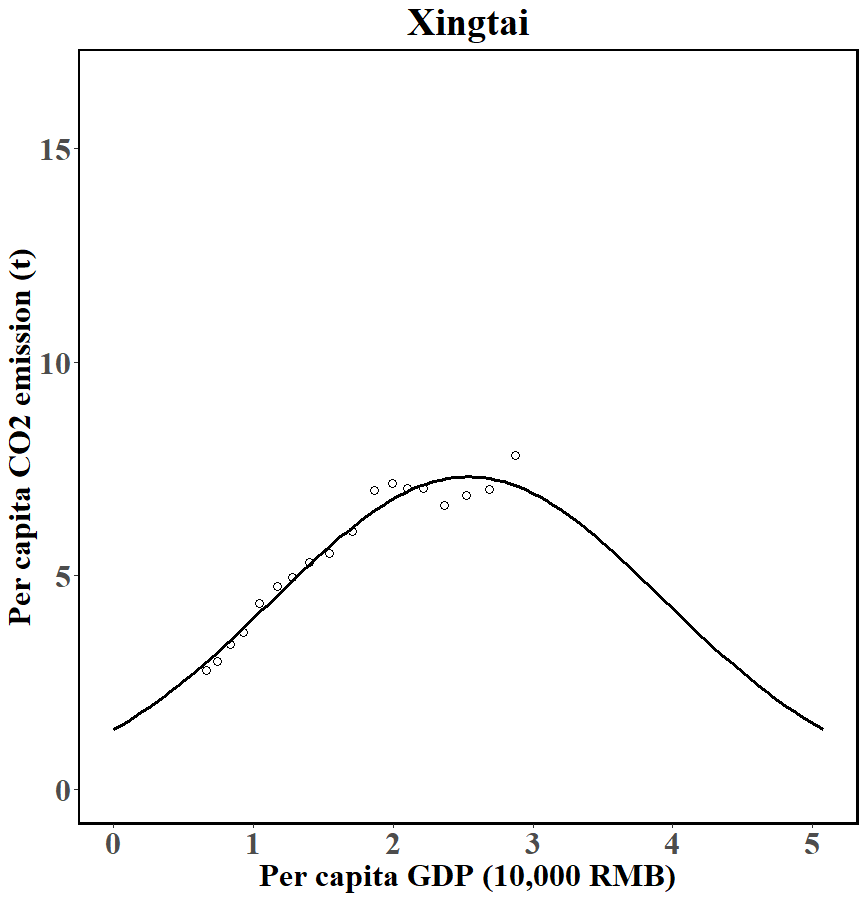

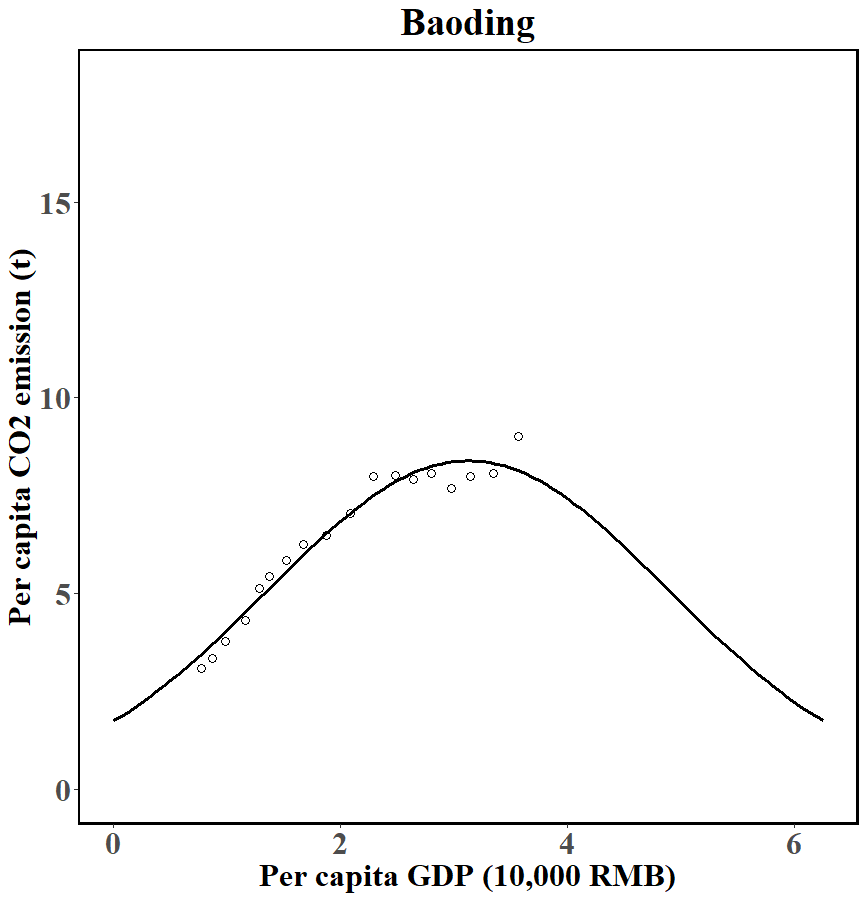

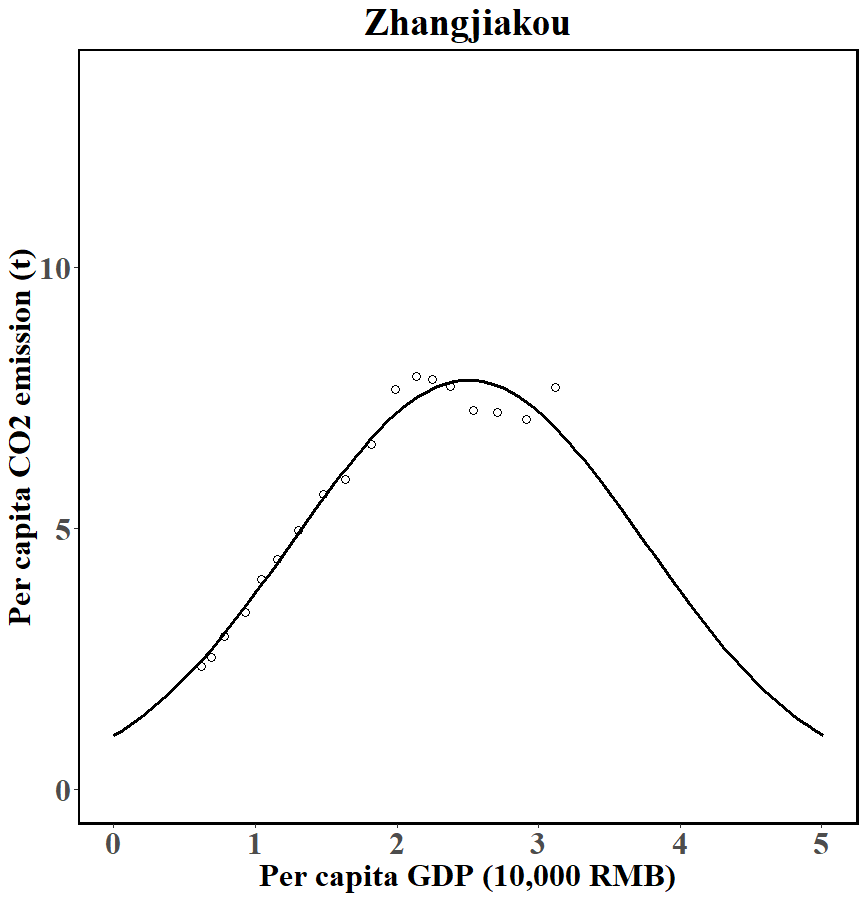

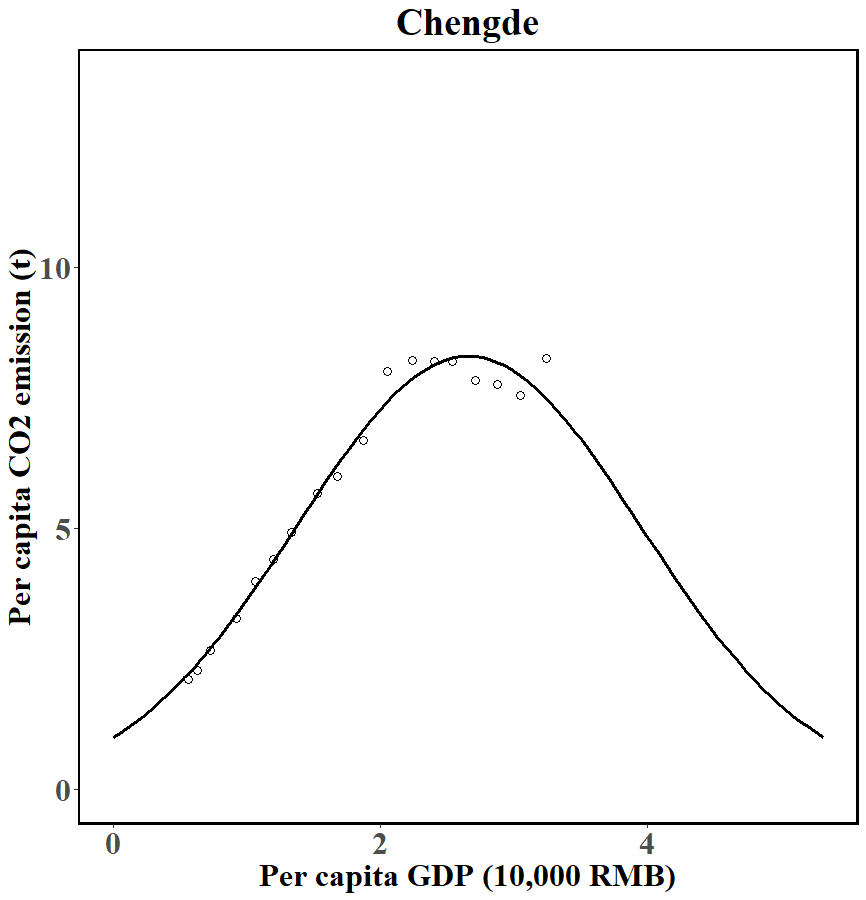

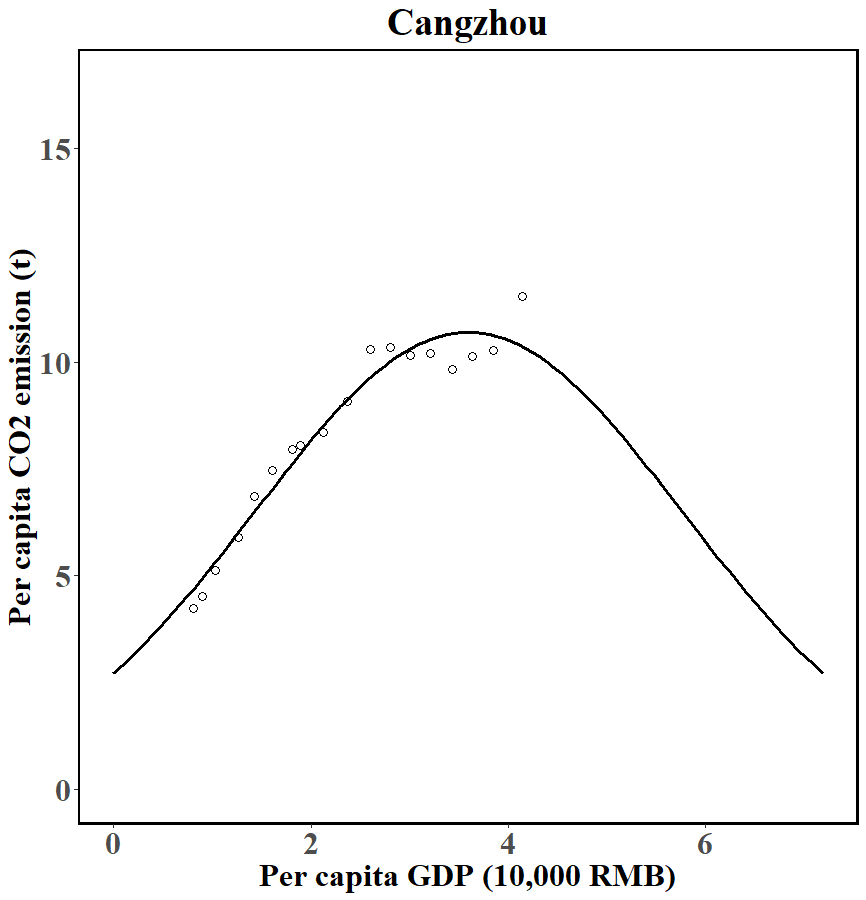

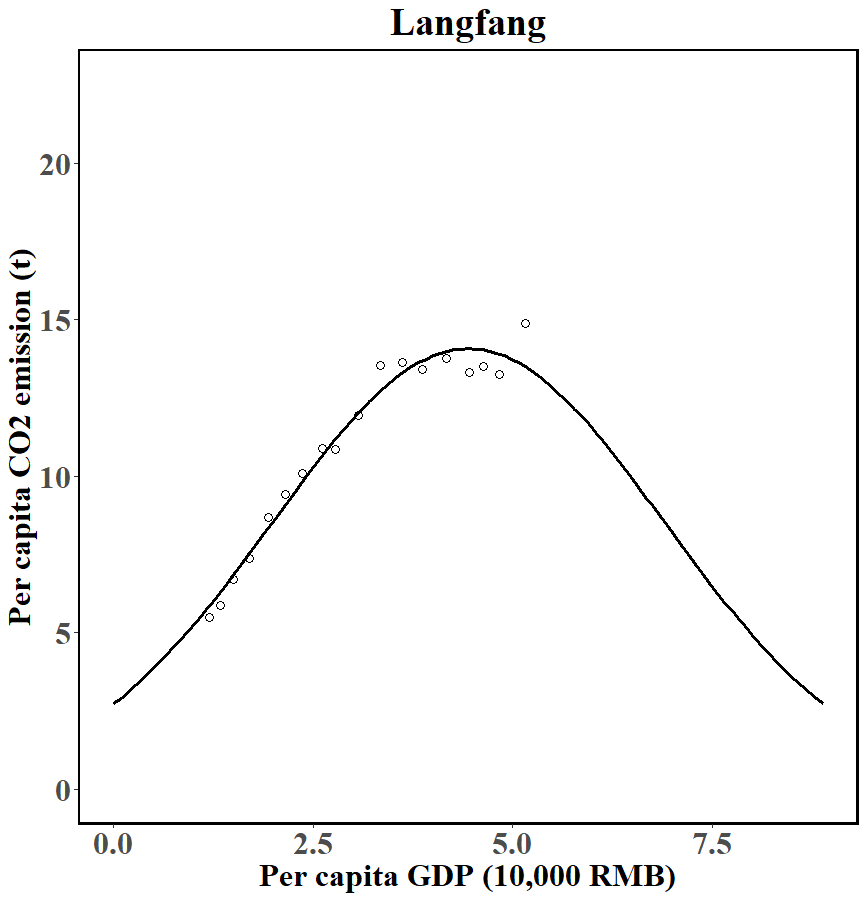

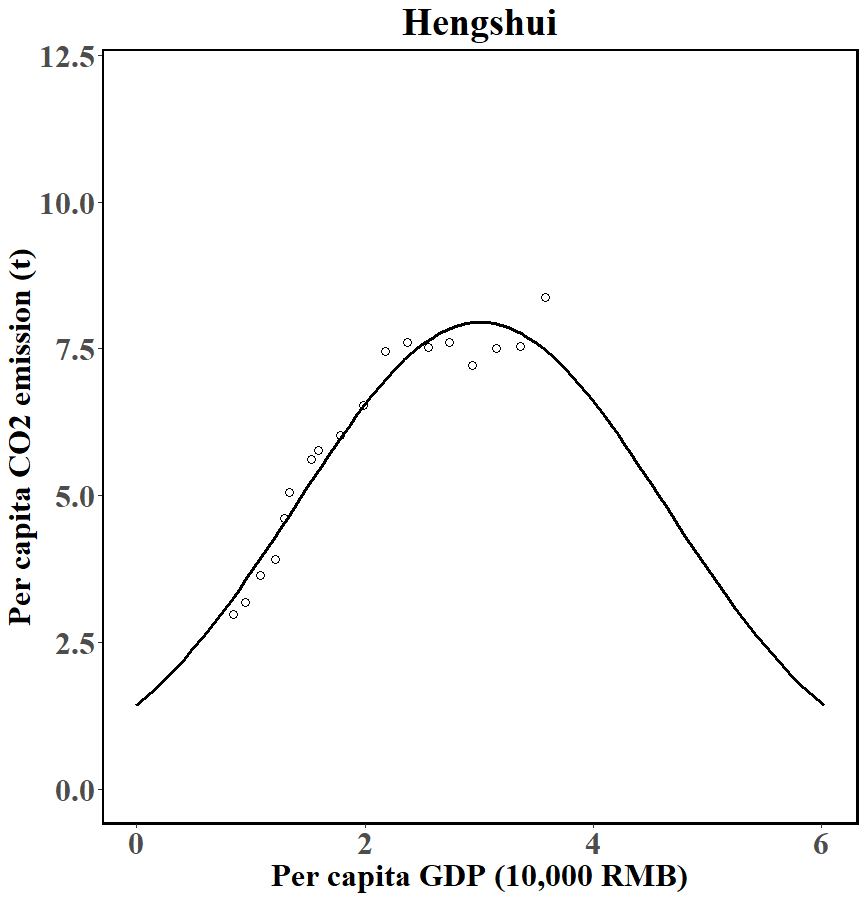

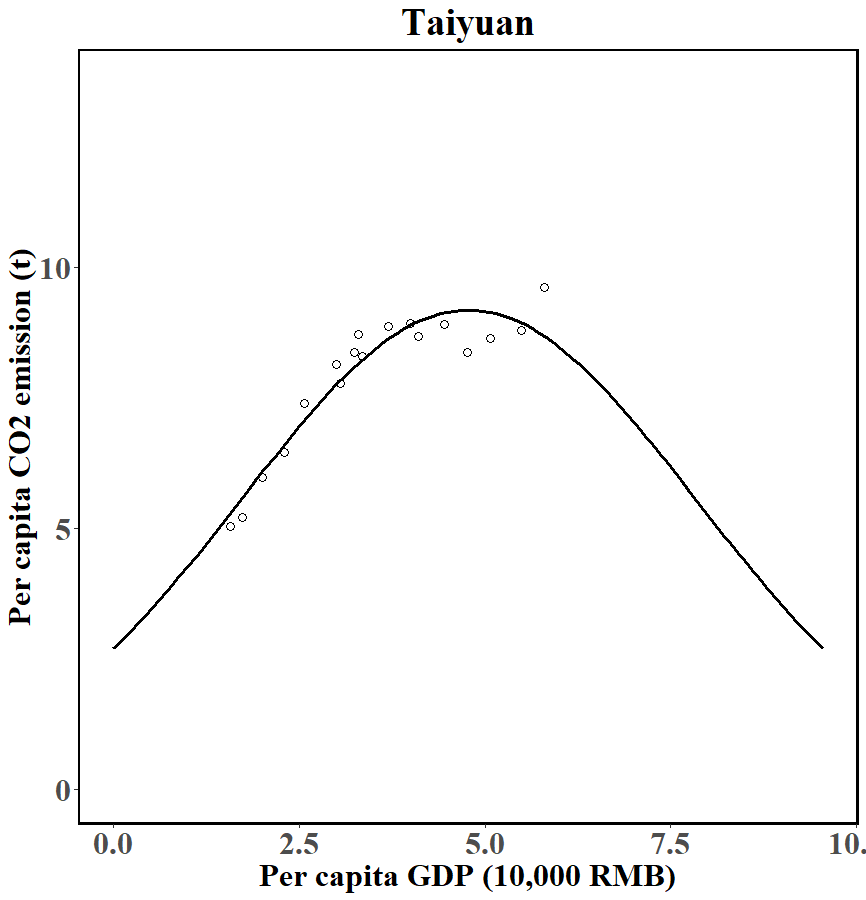

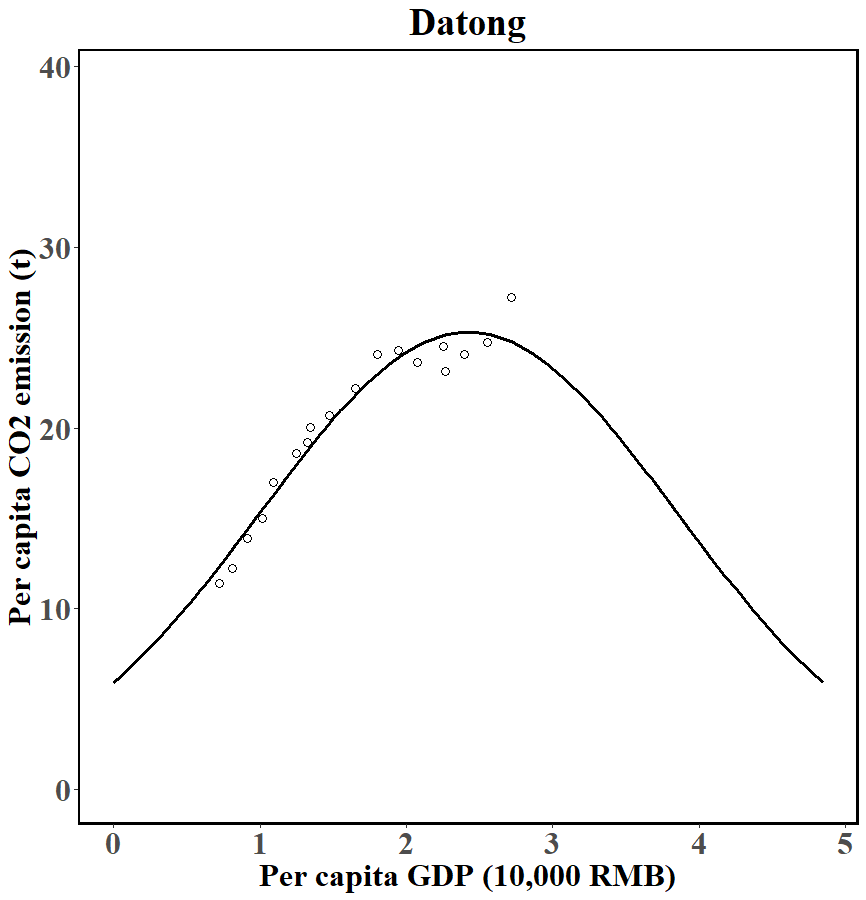

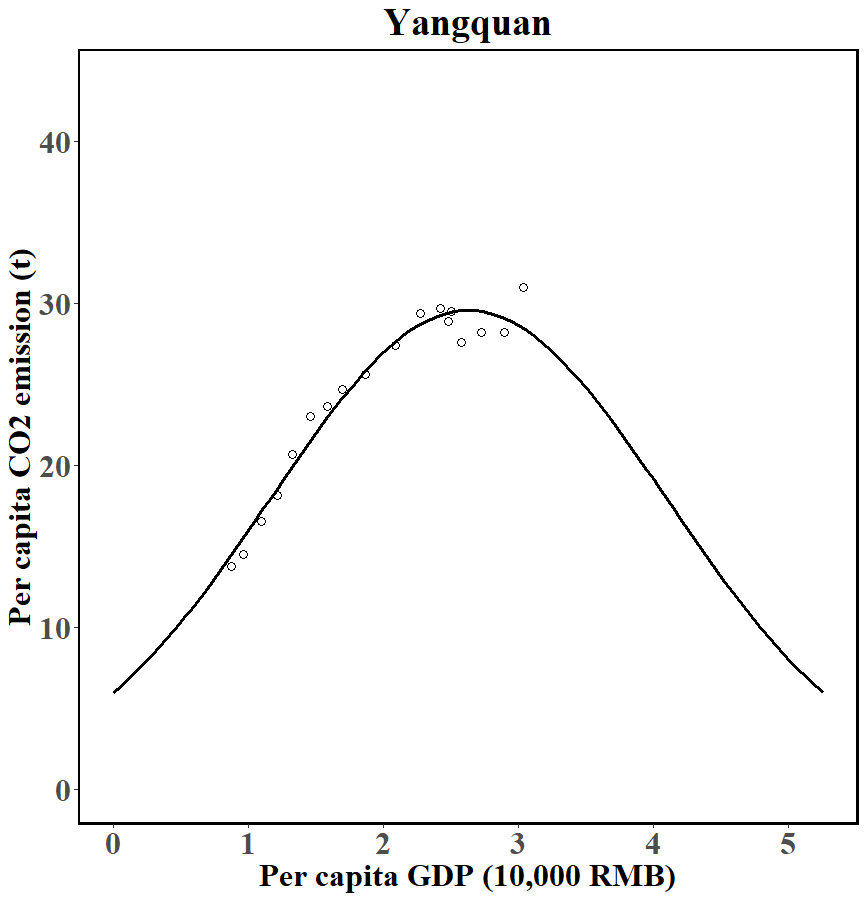

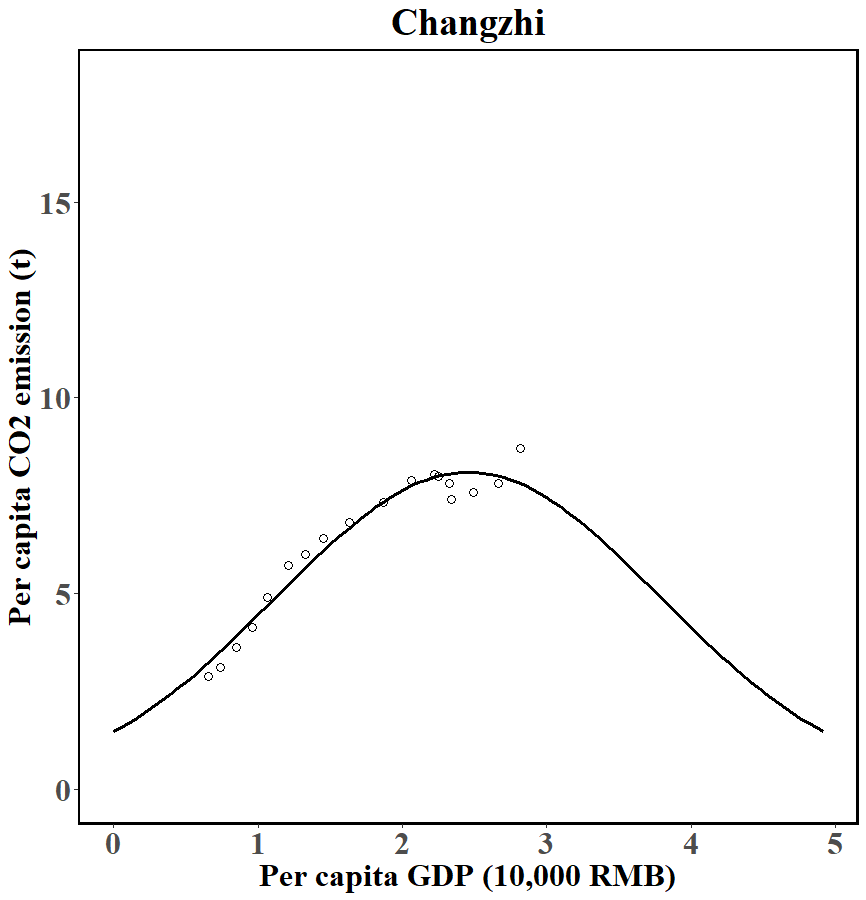

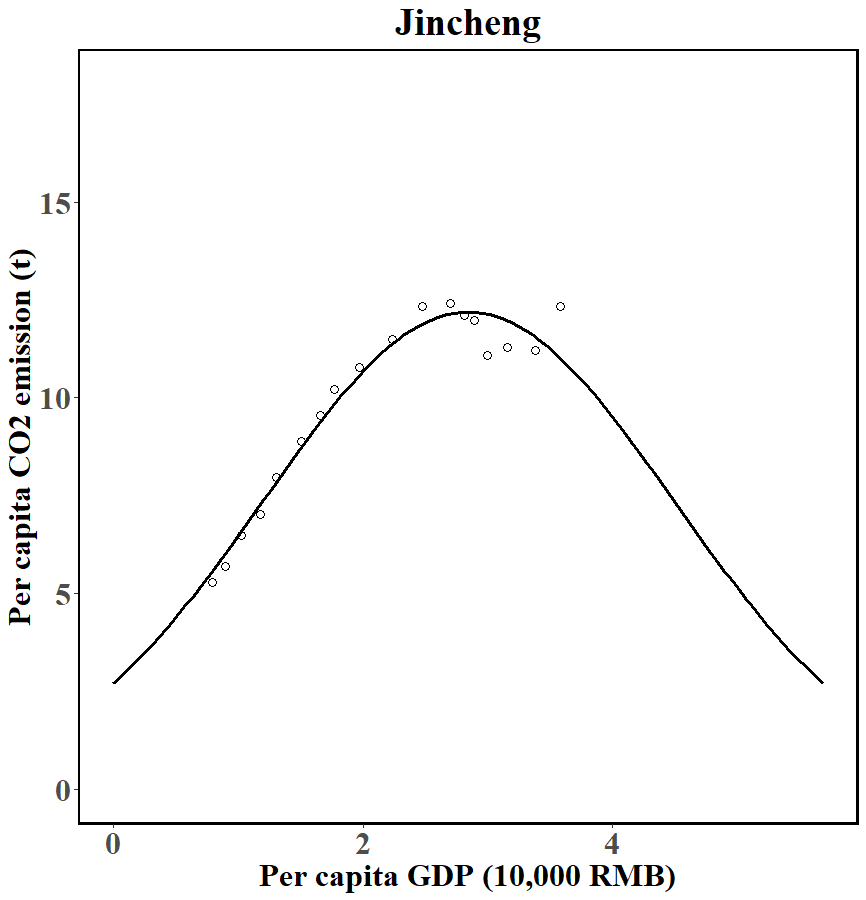

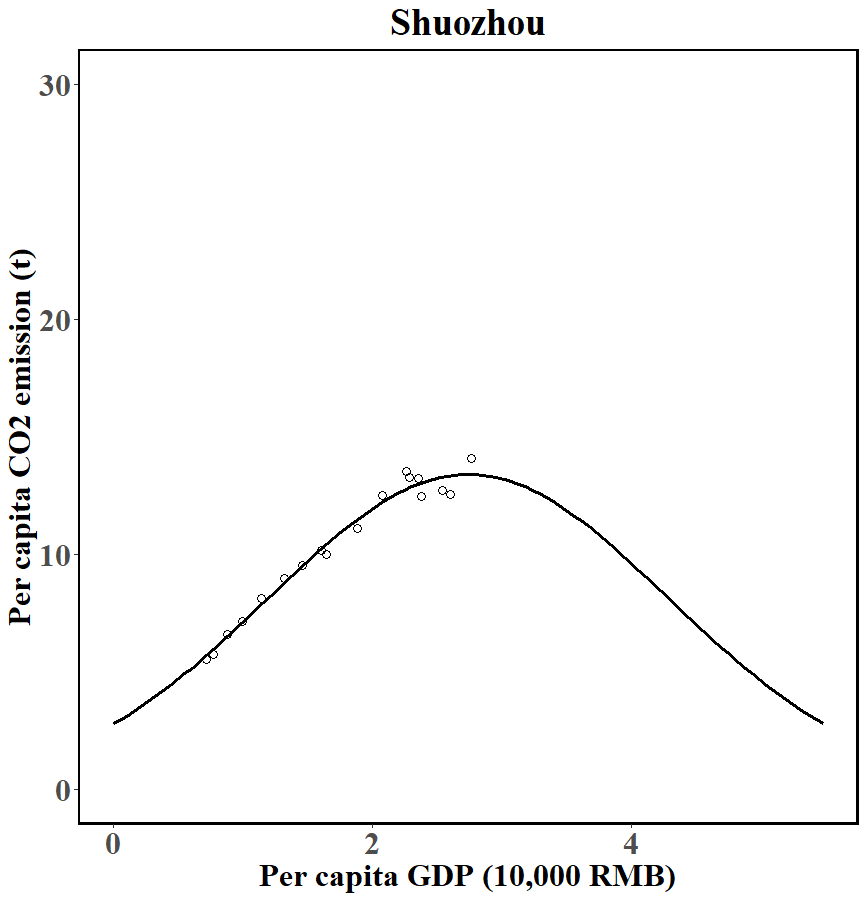

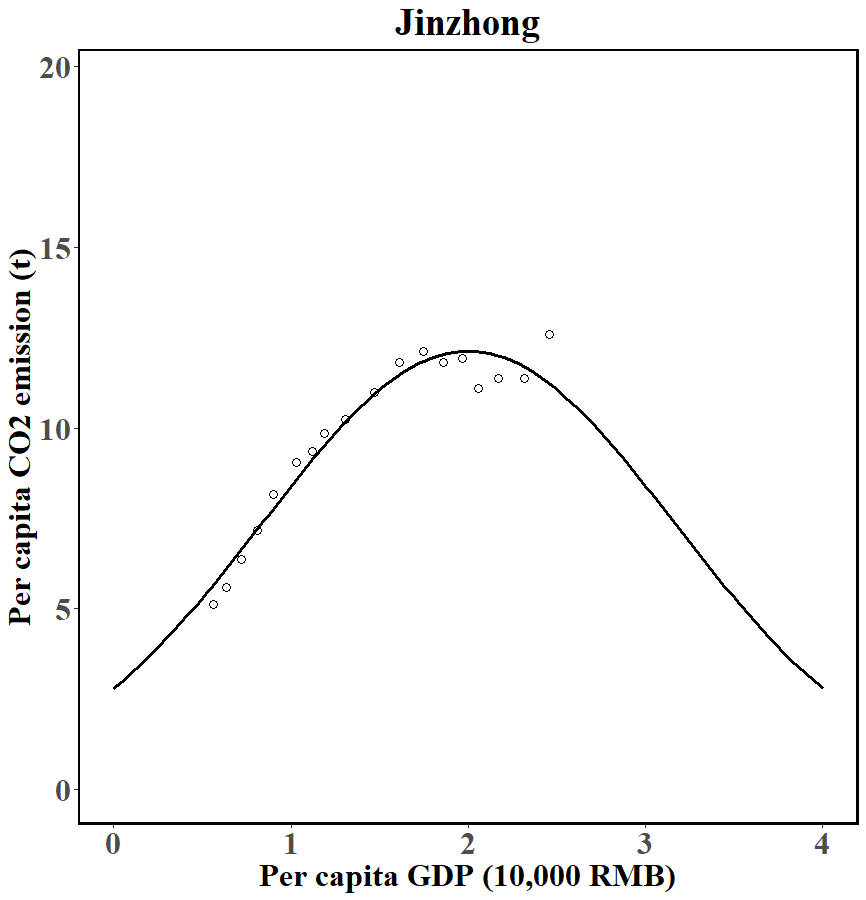

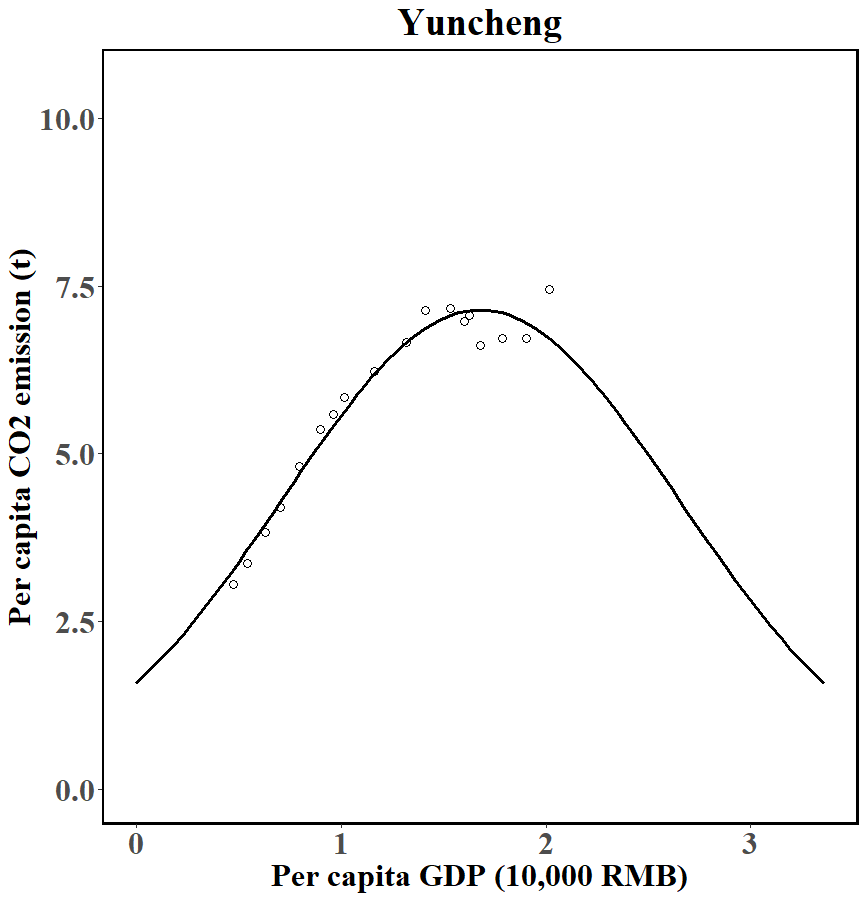

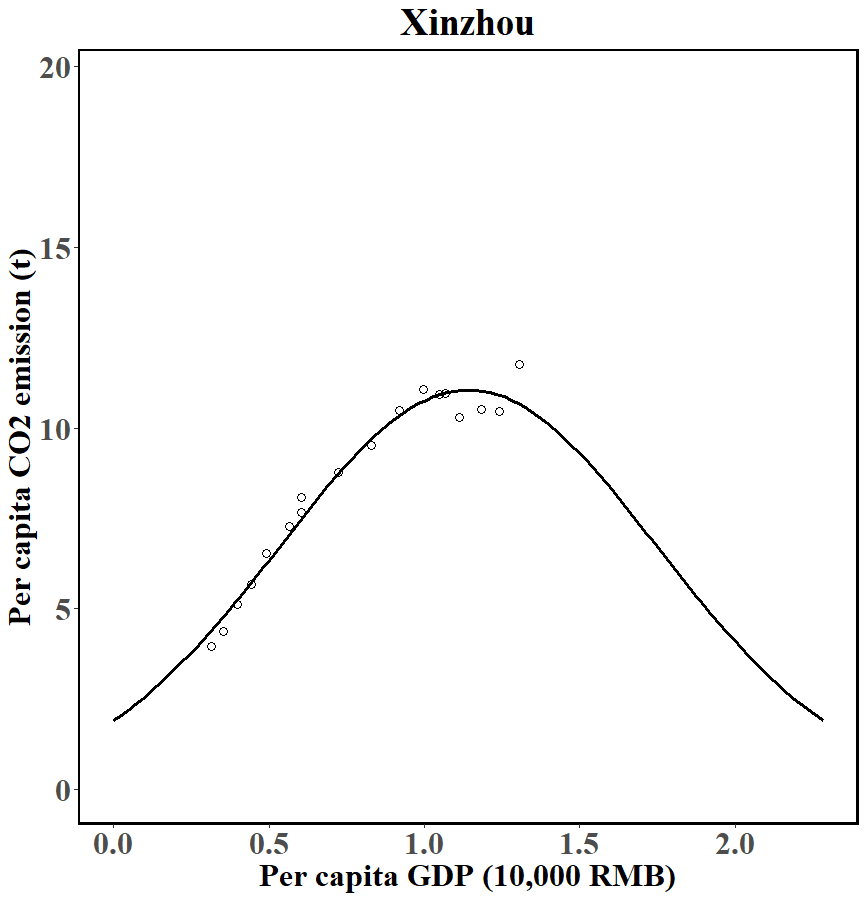

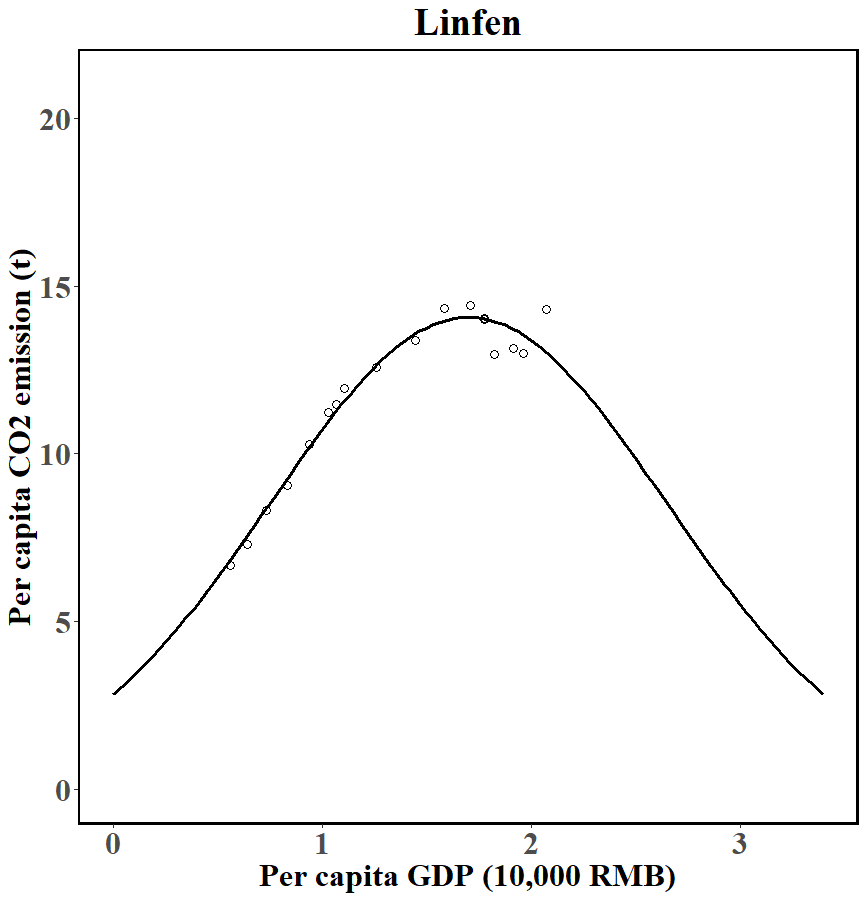

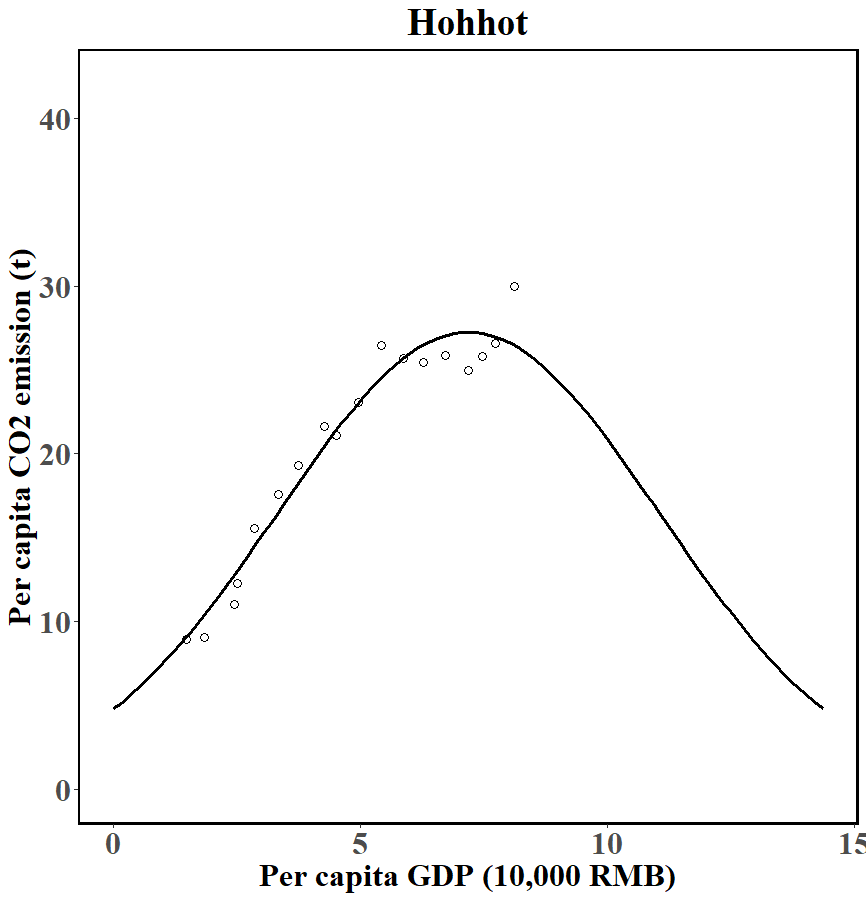

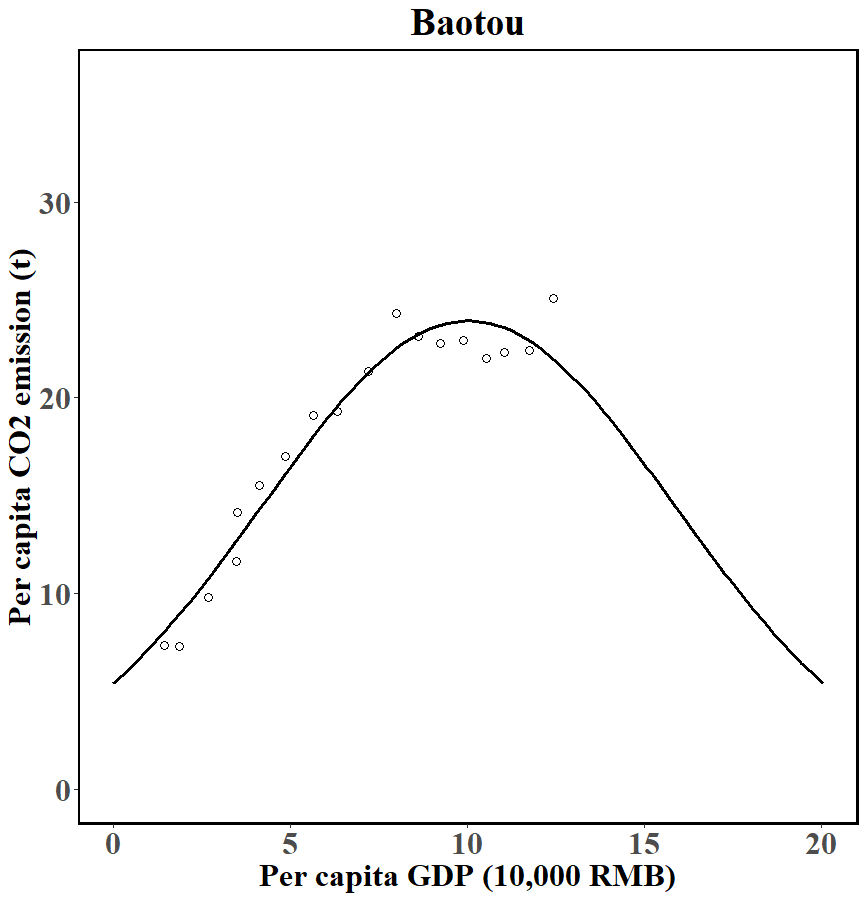

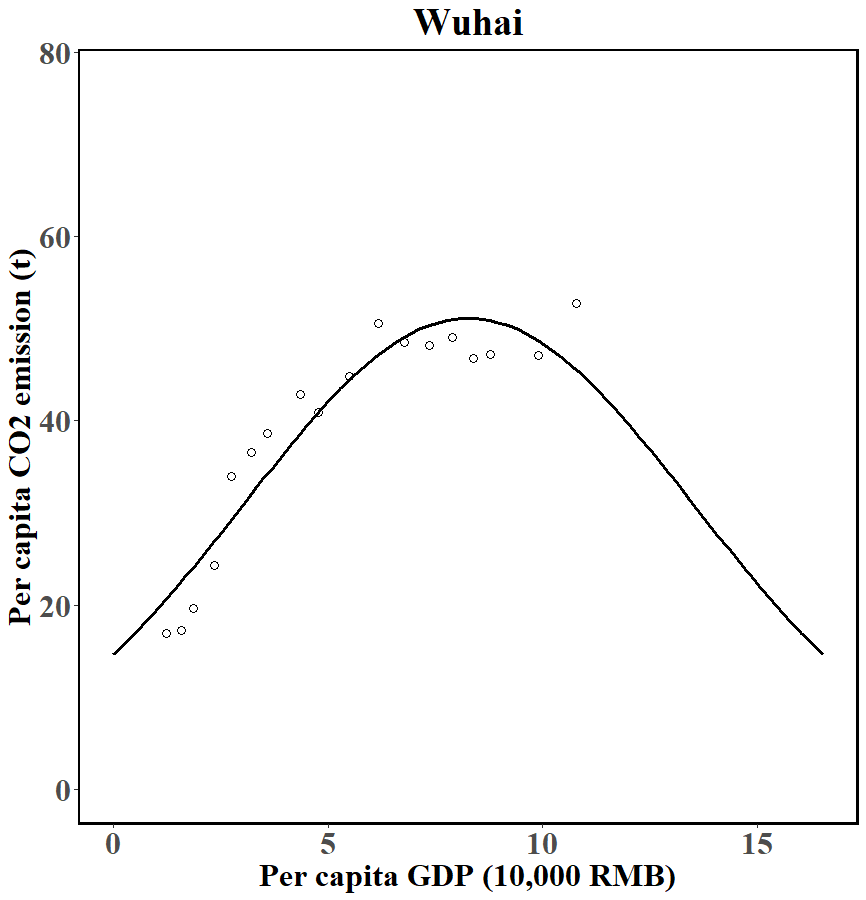

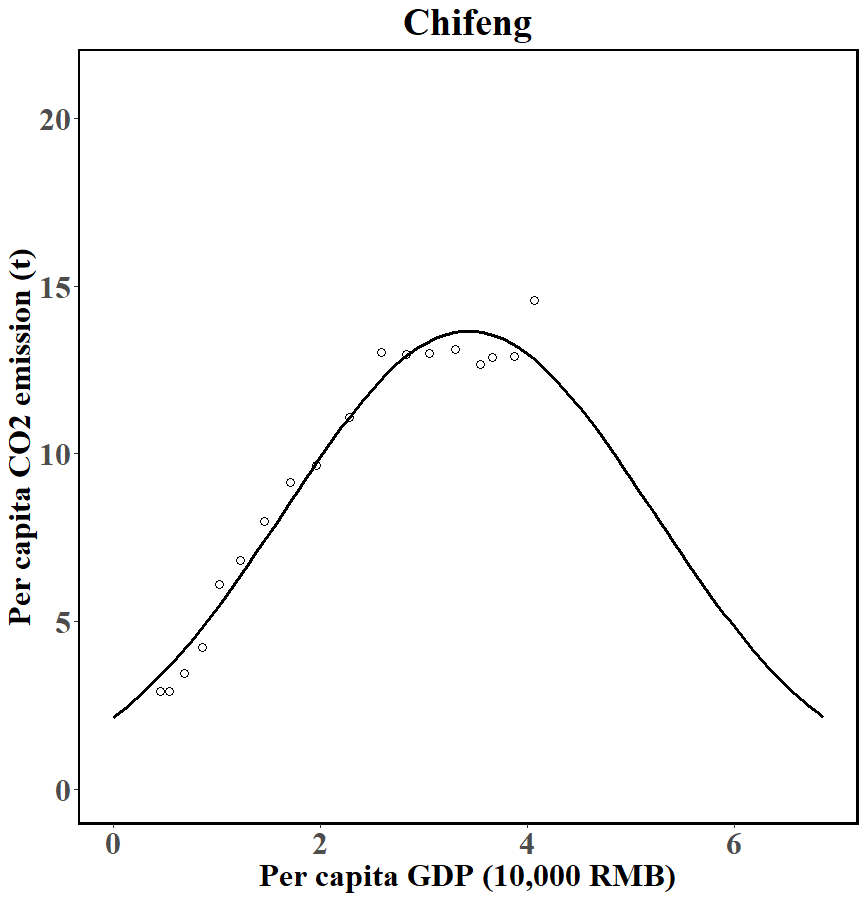

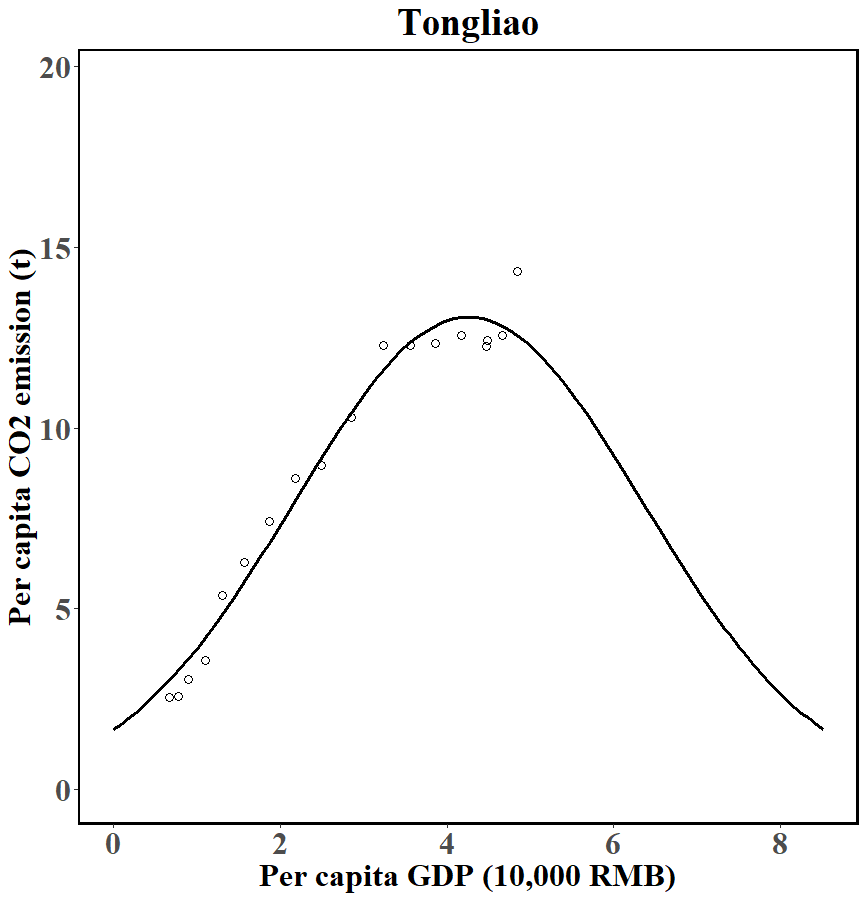

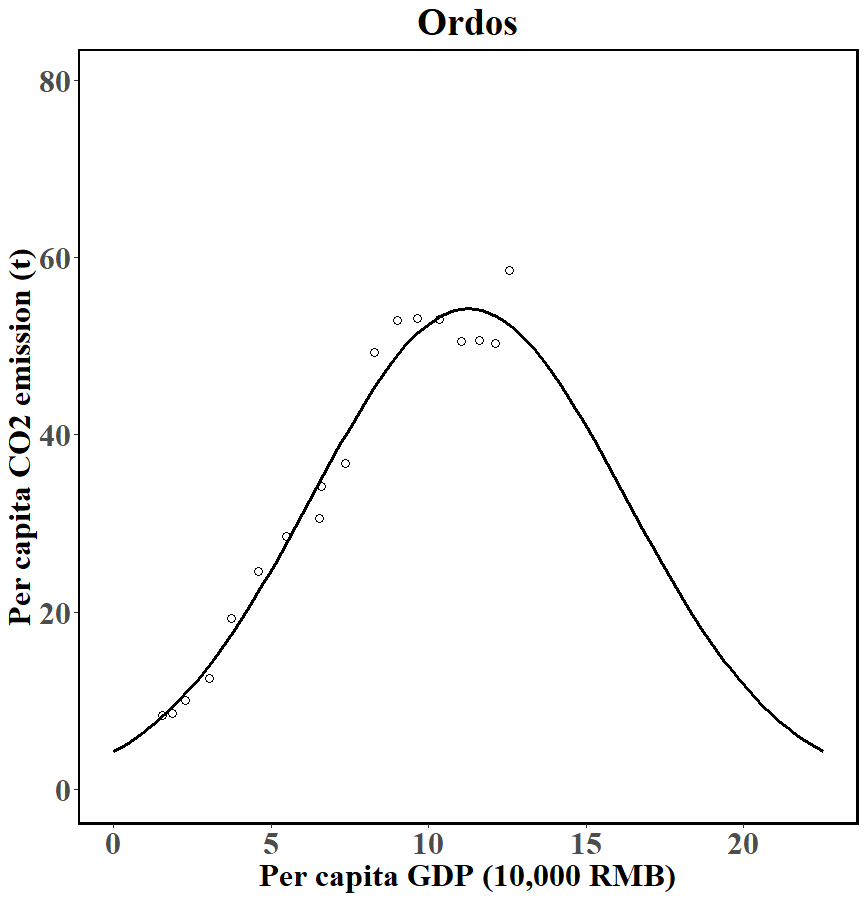

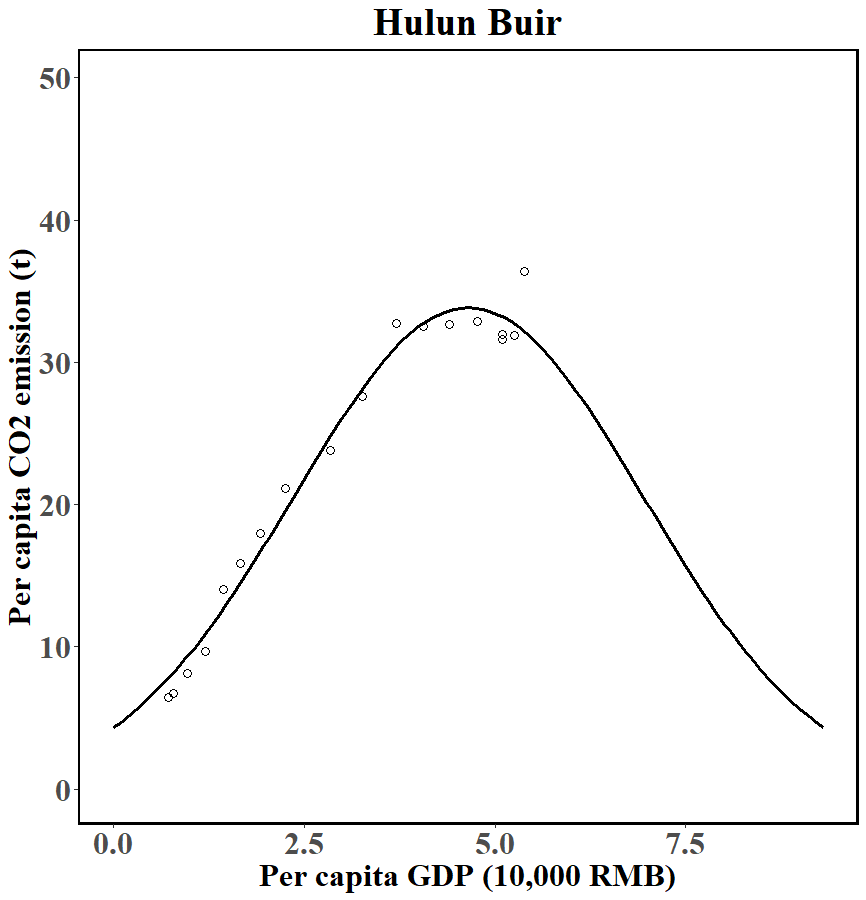

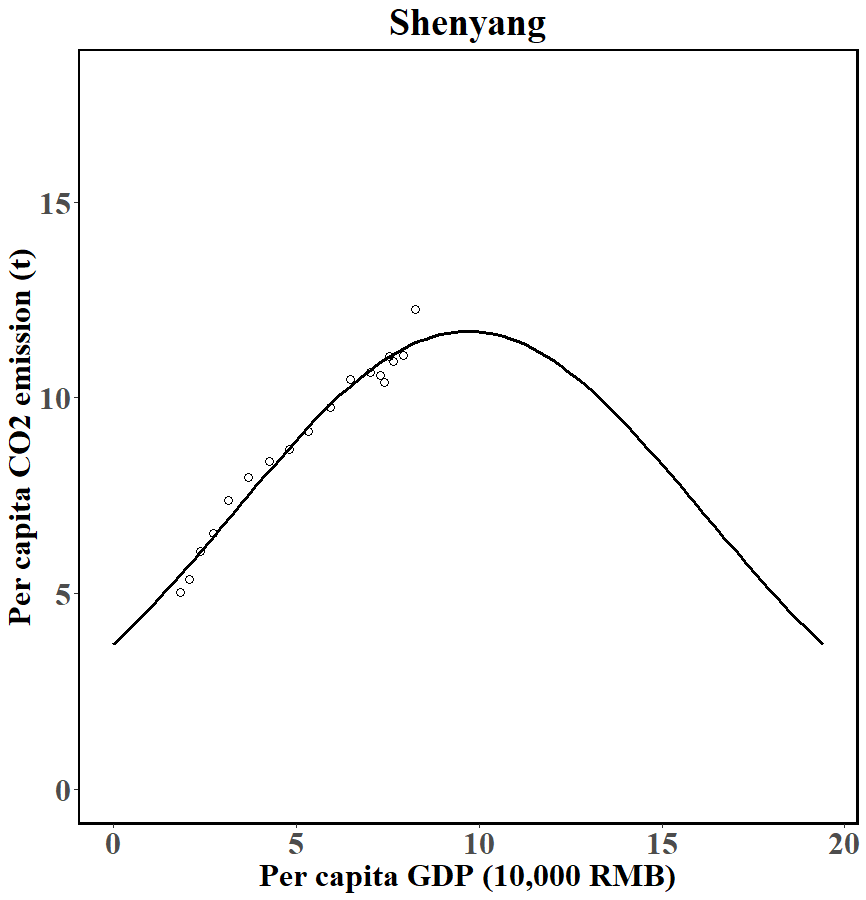

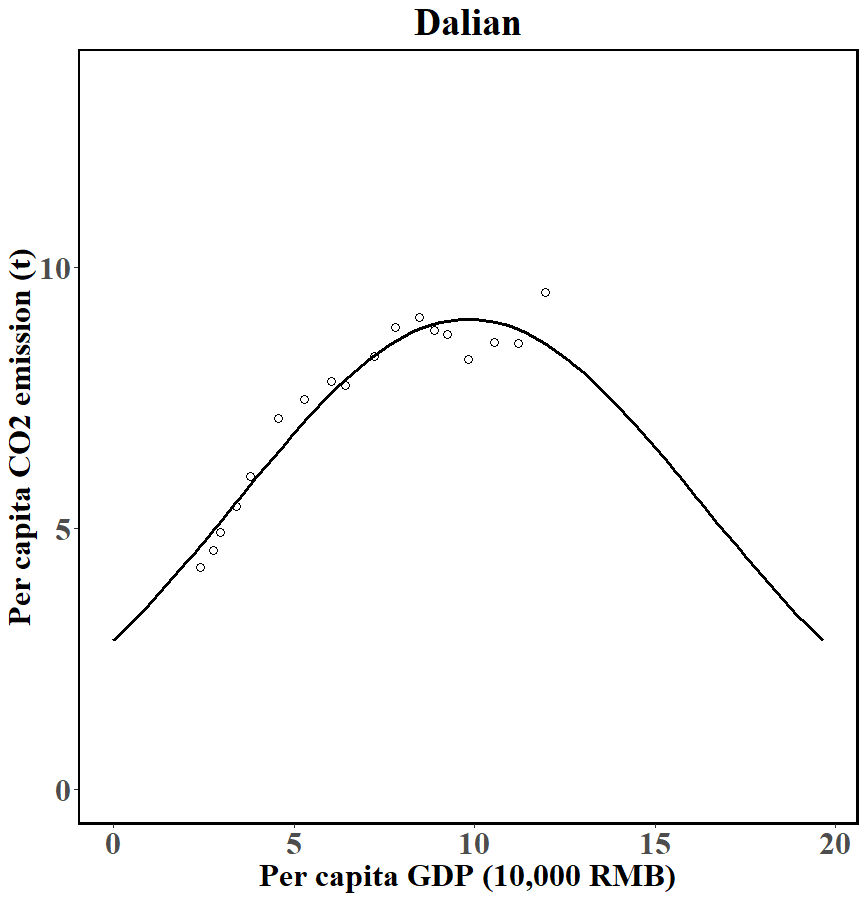

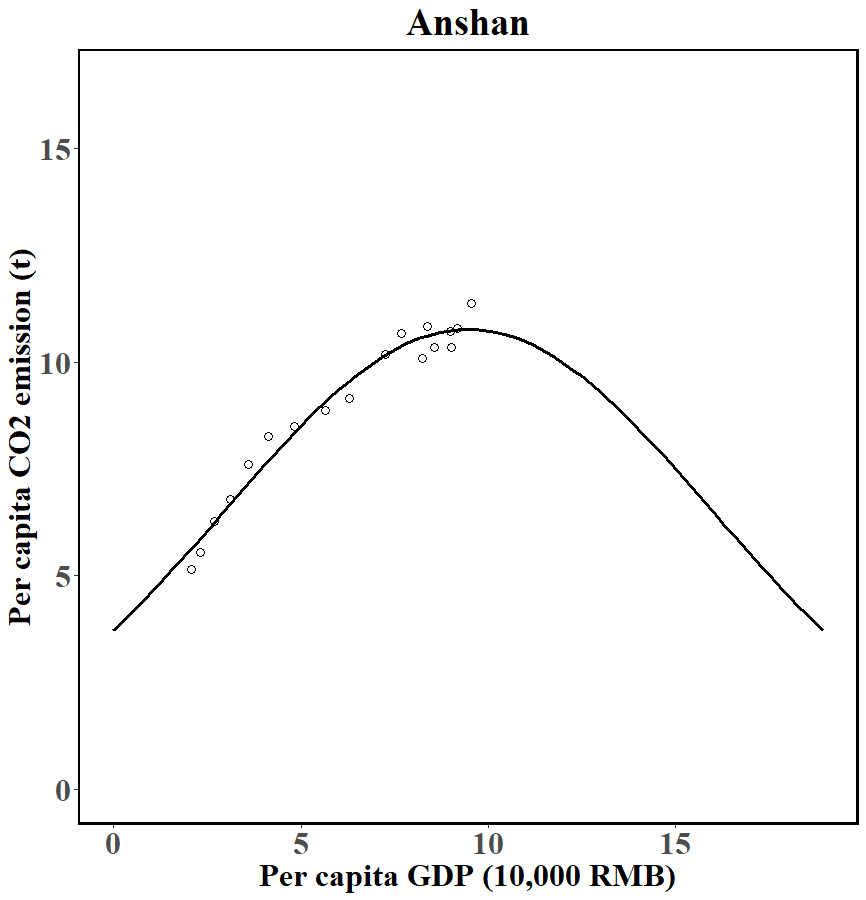

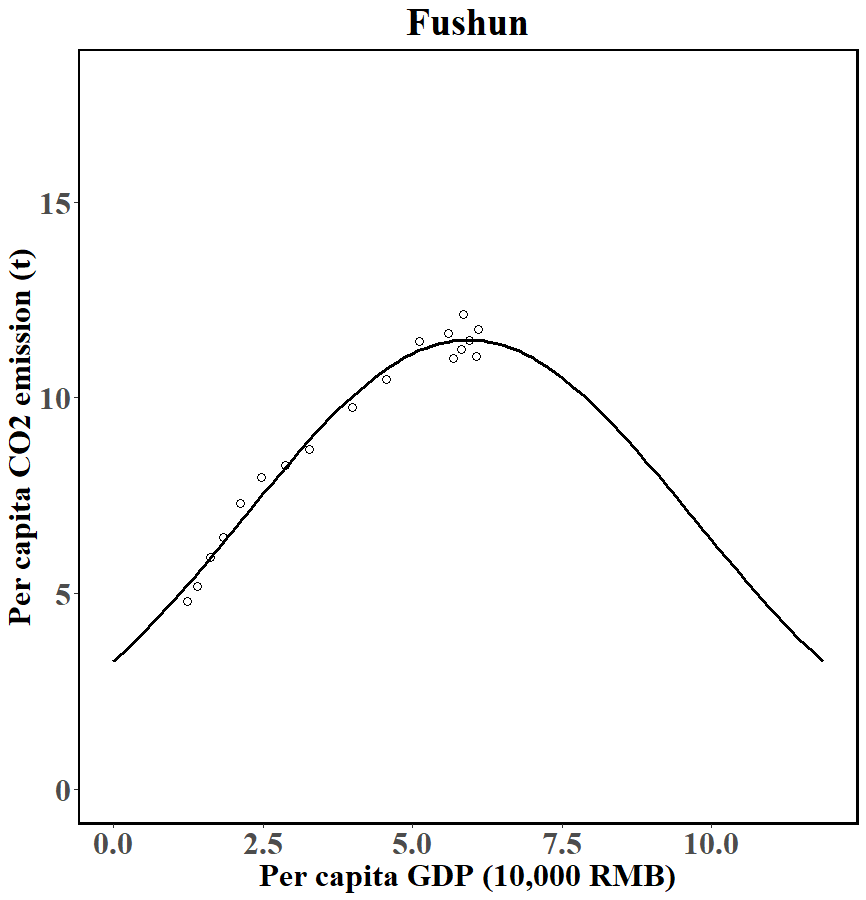

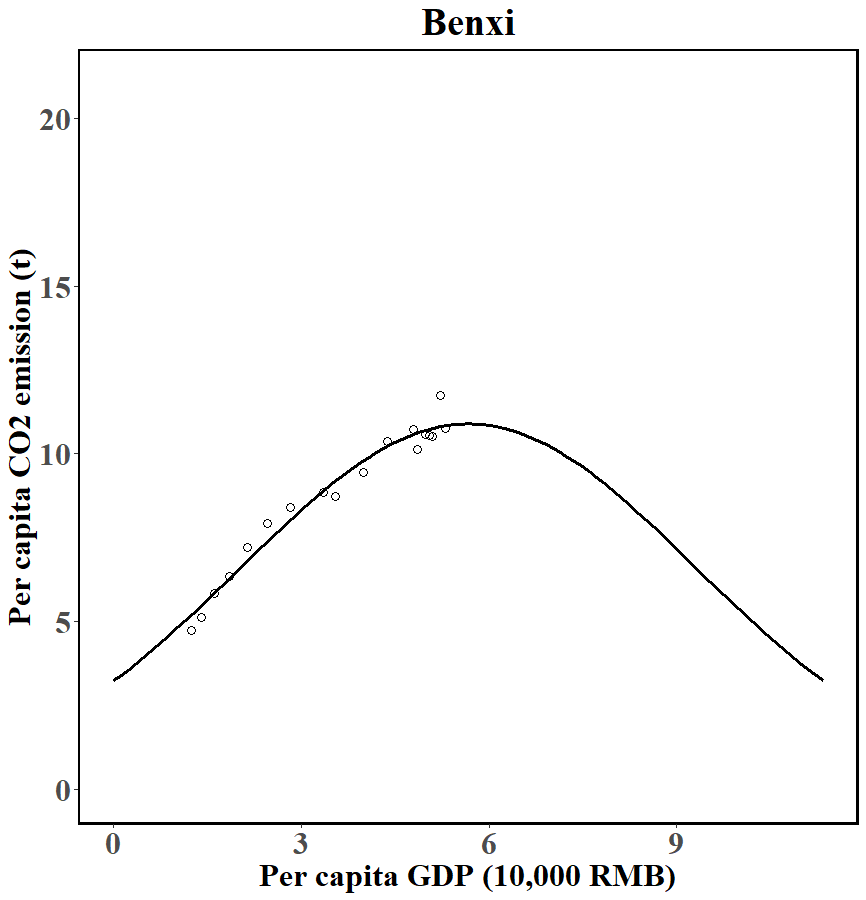


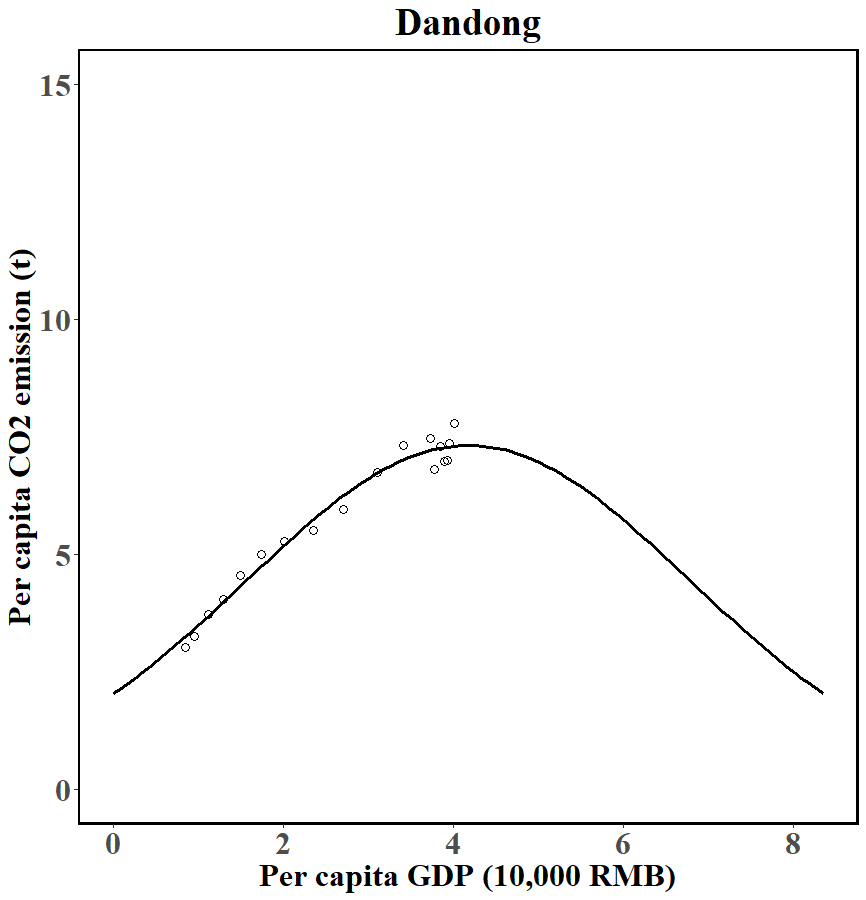

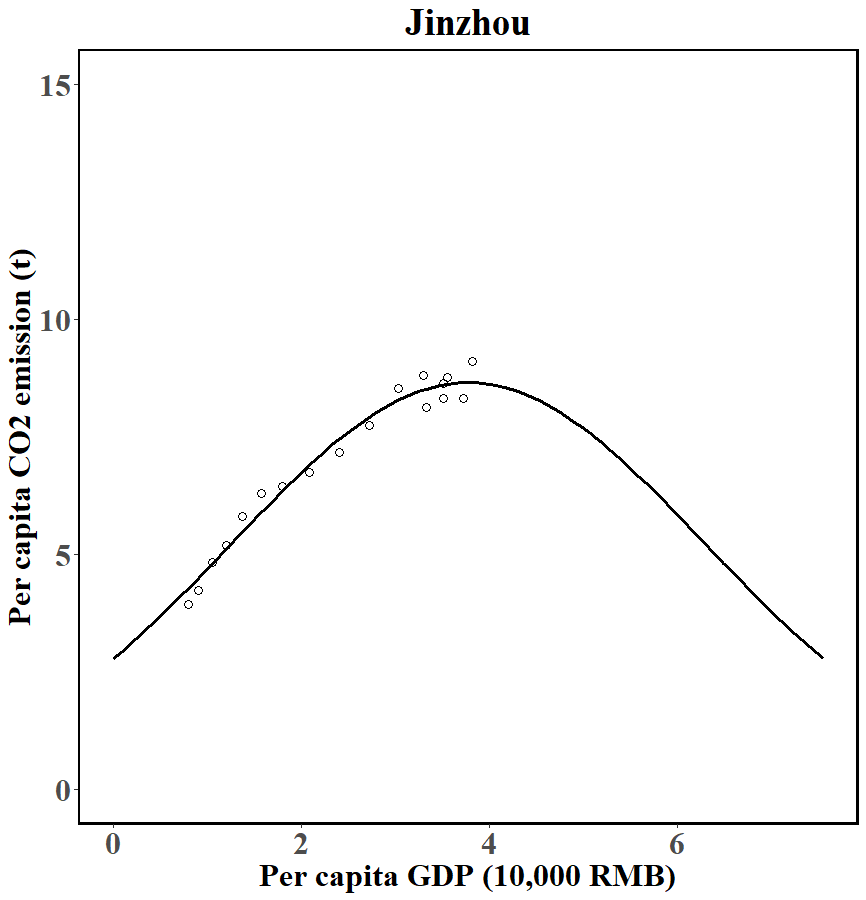

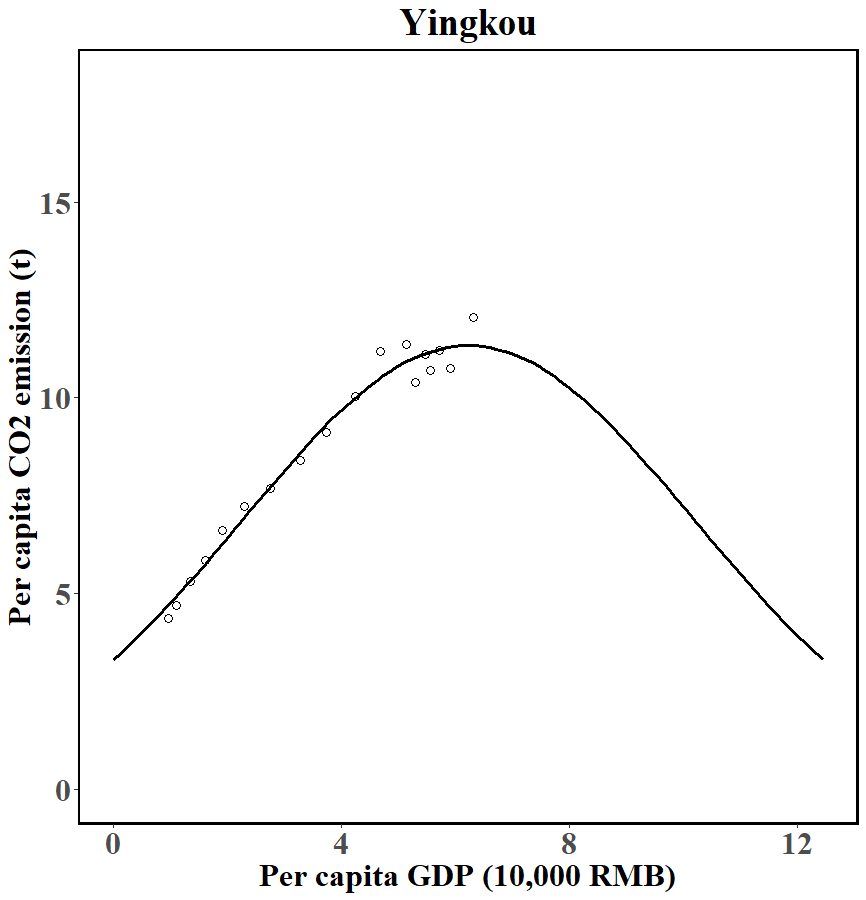

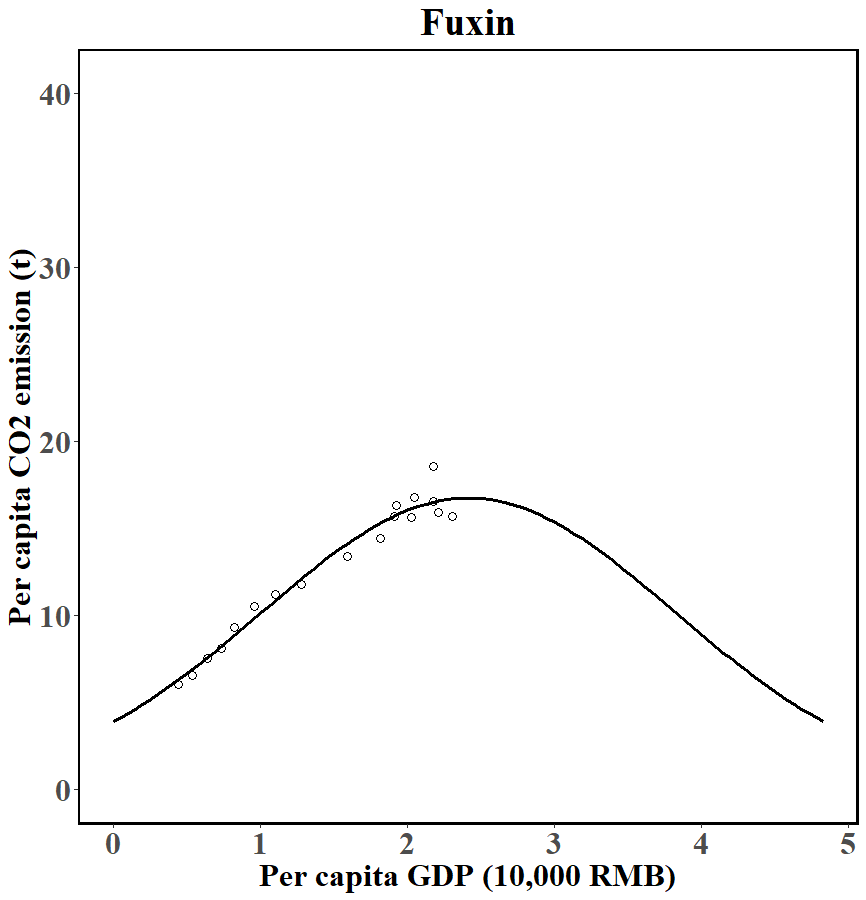

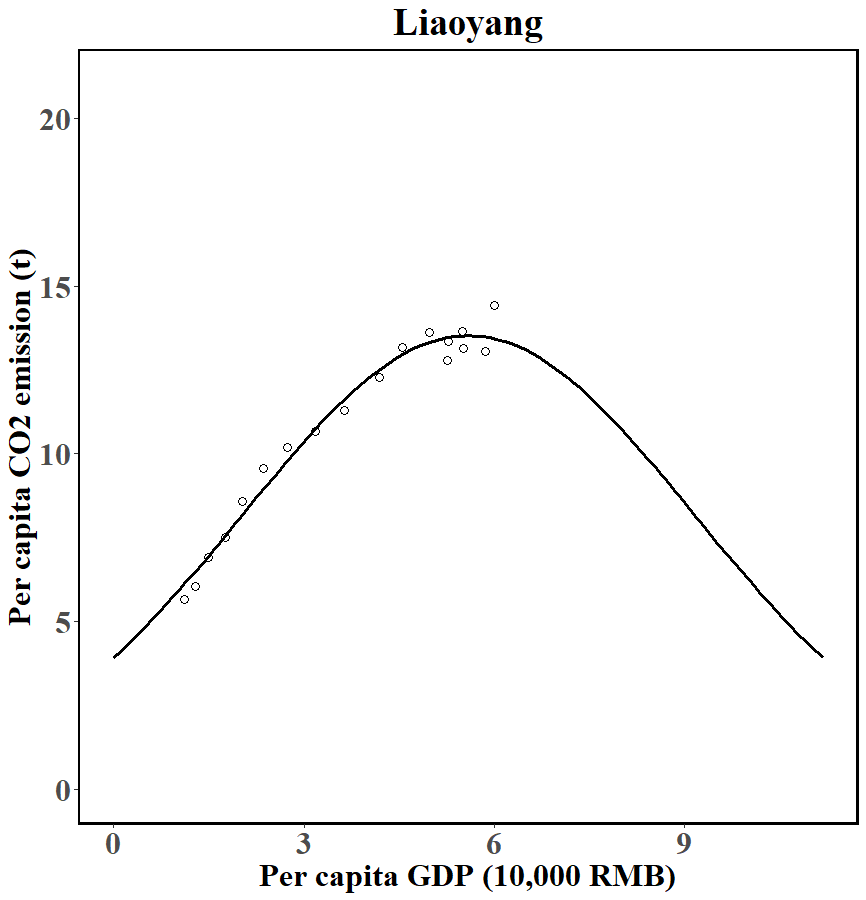
.
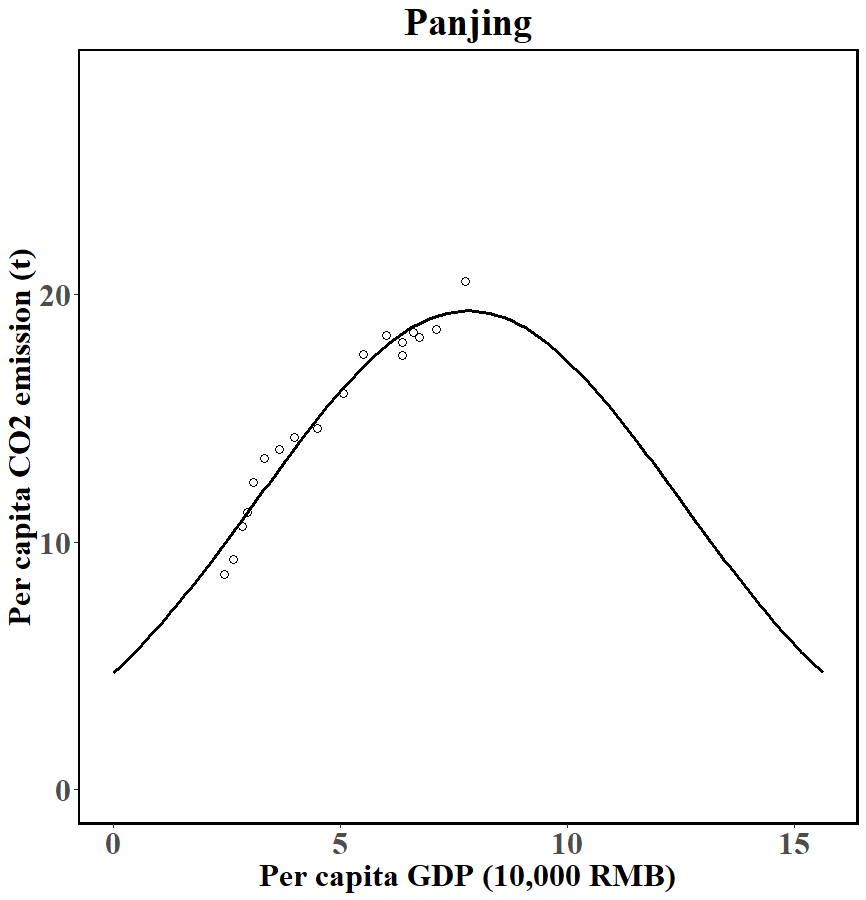

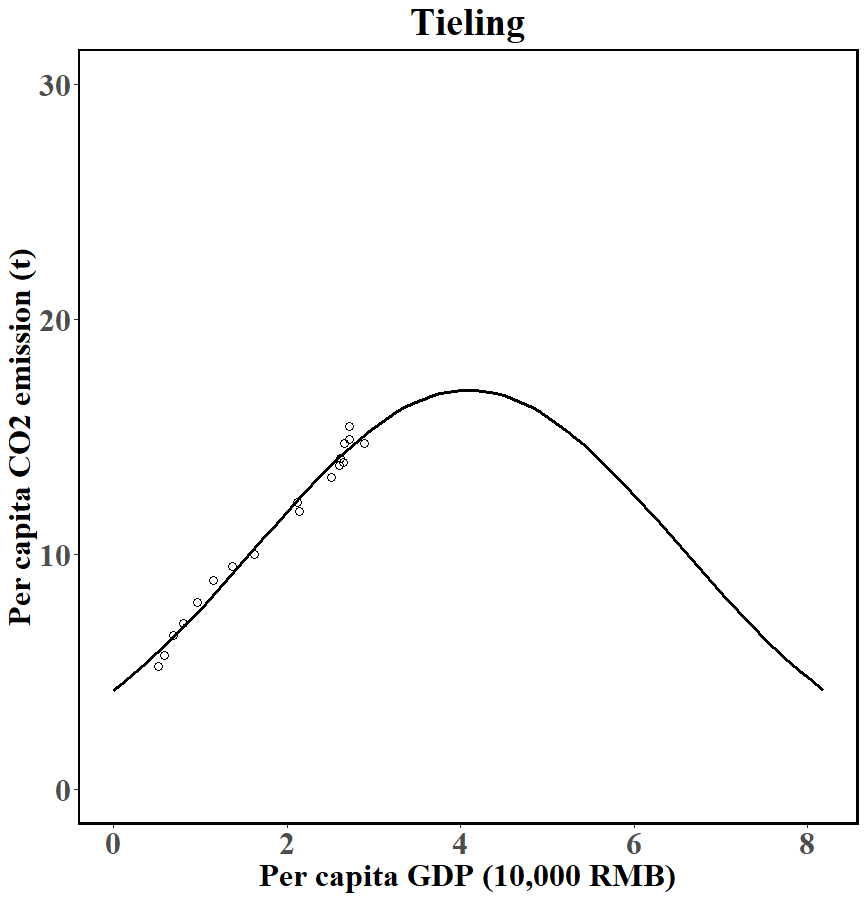

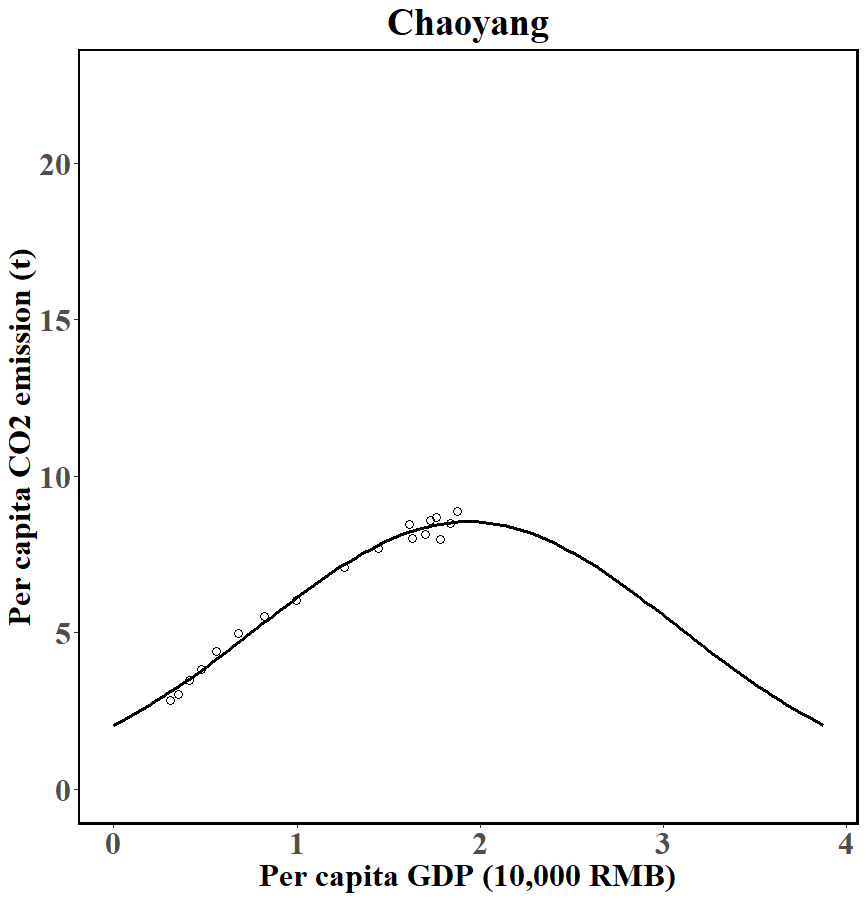

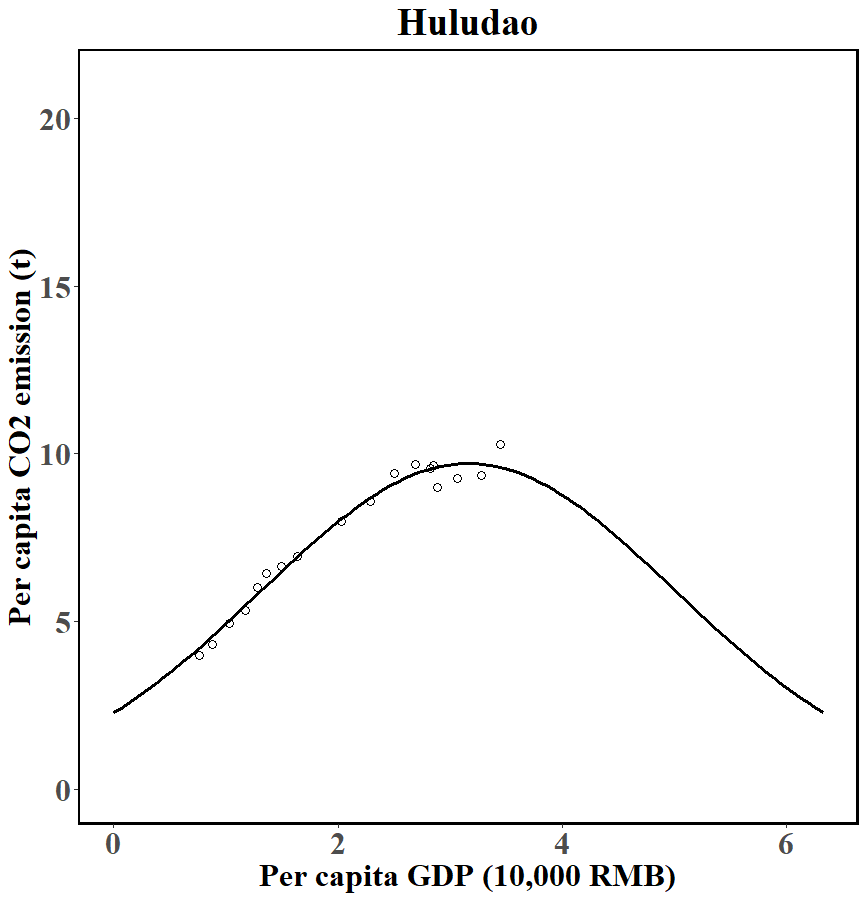

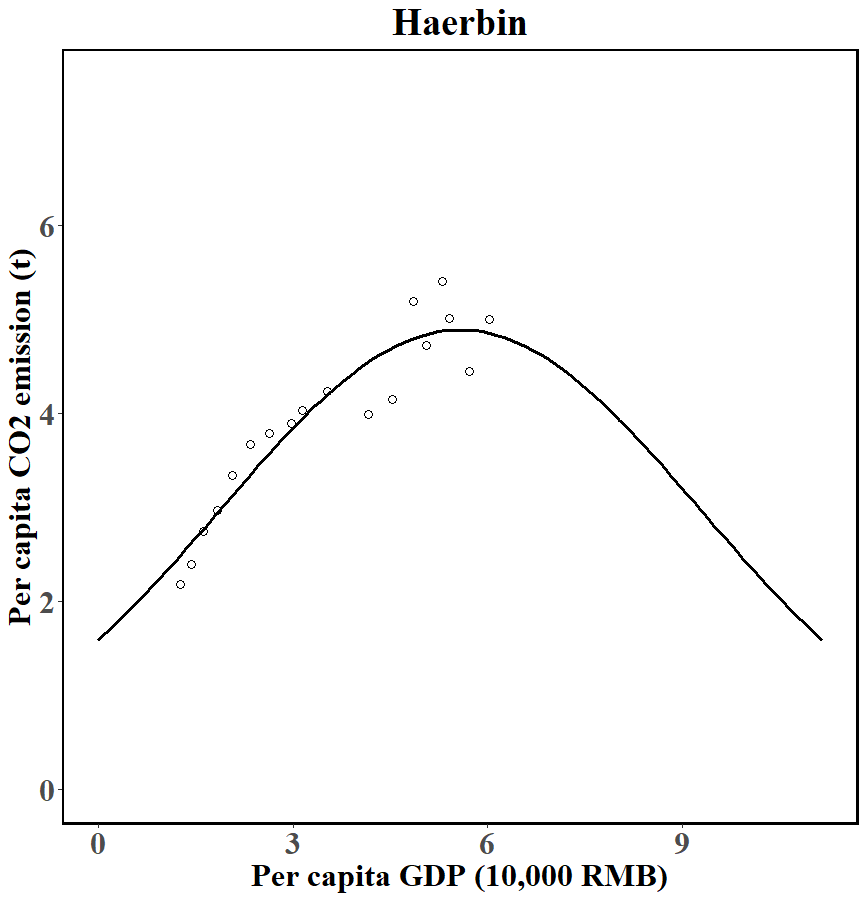

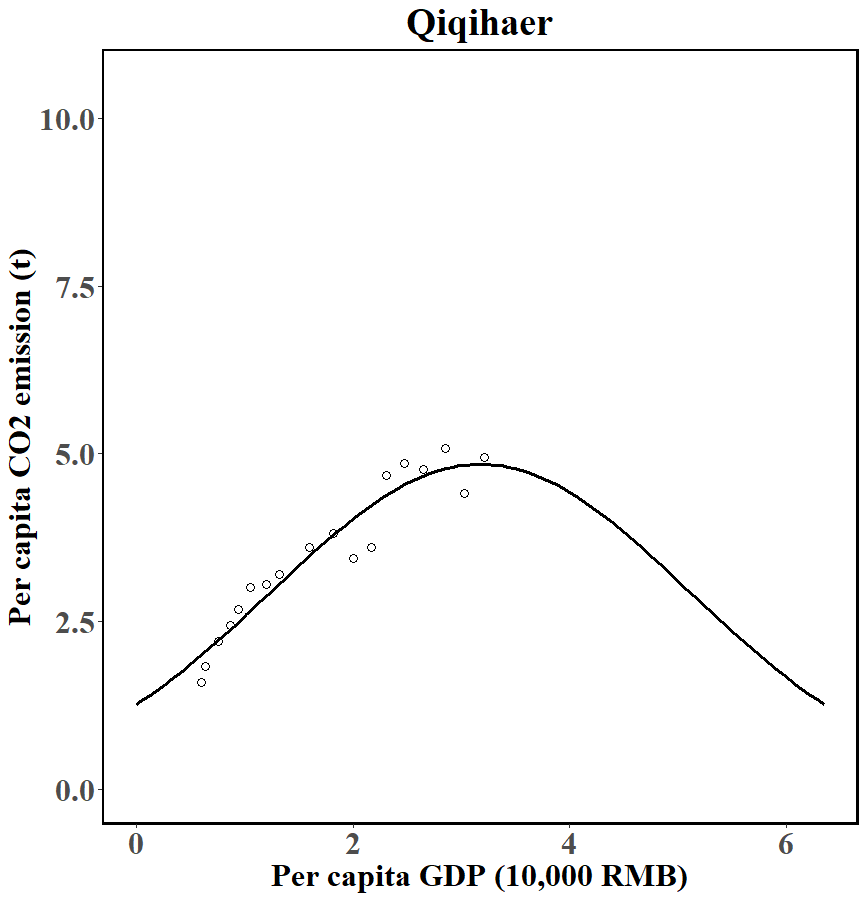

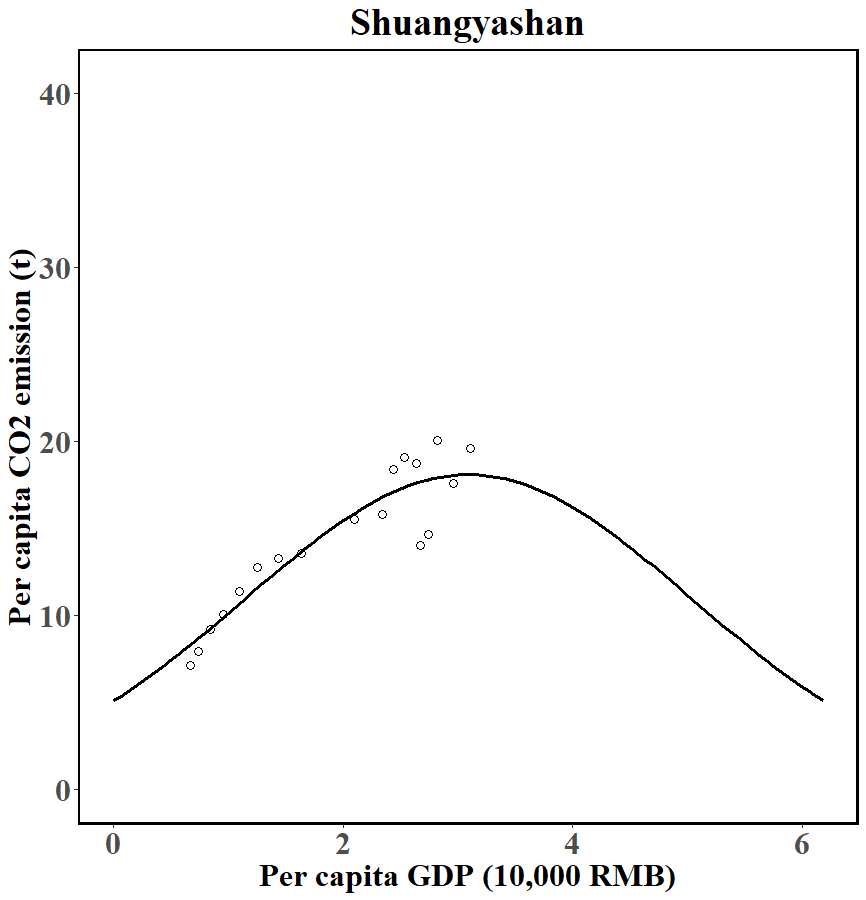

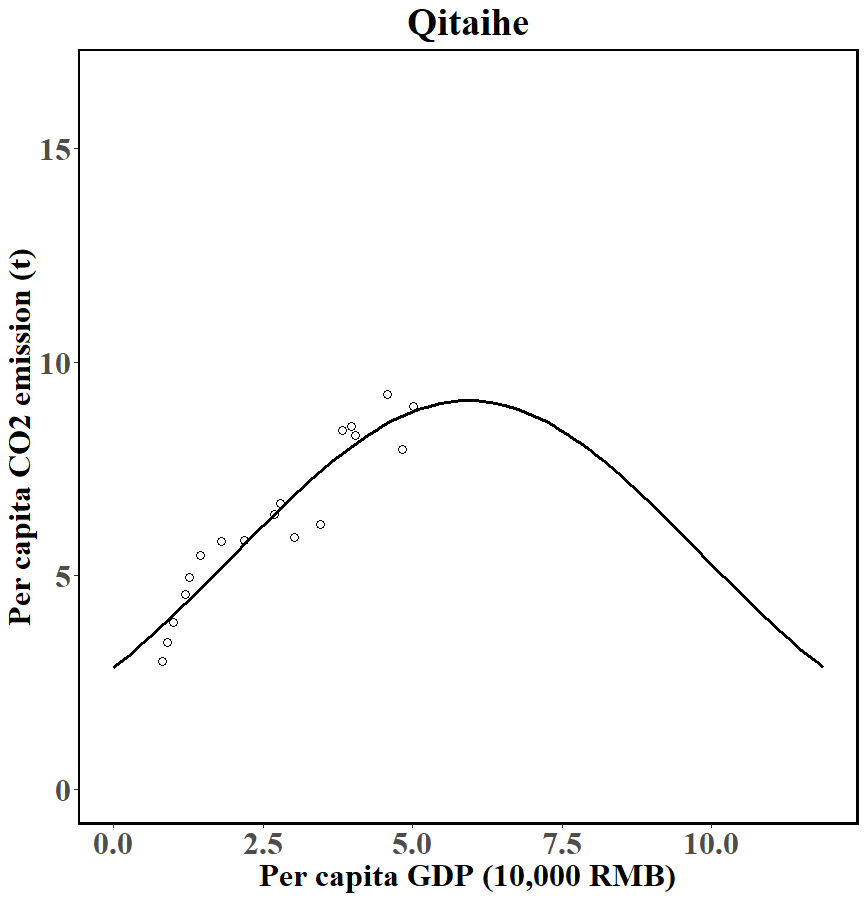

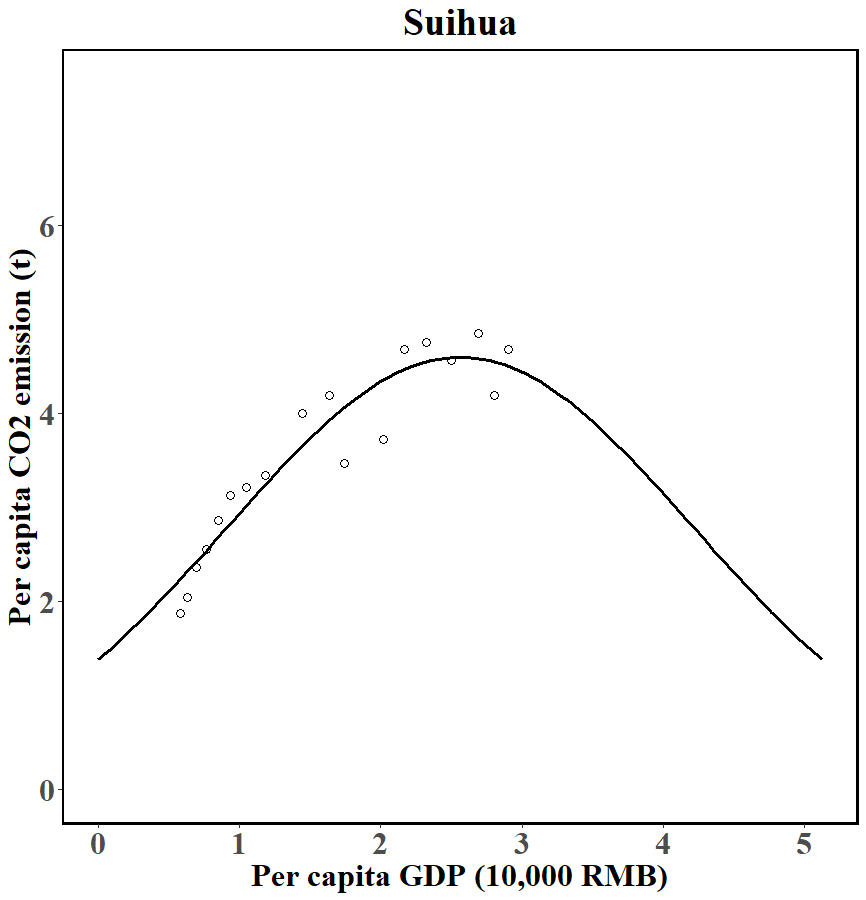

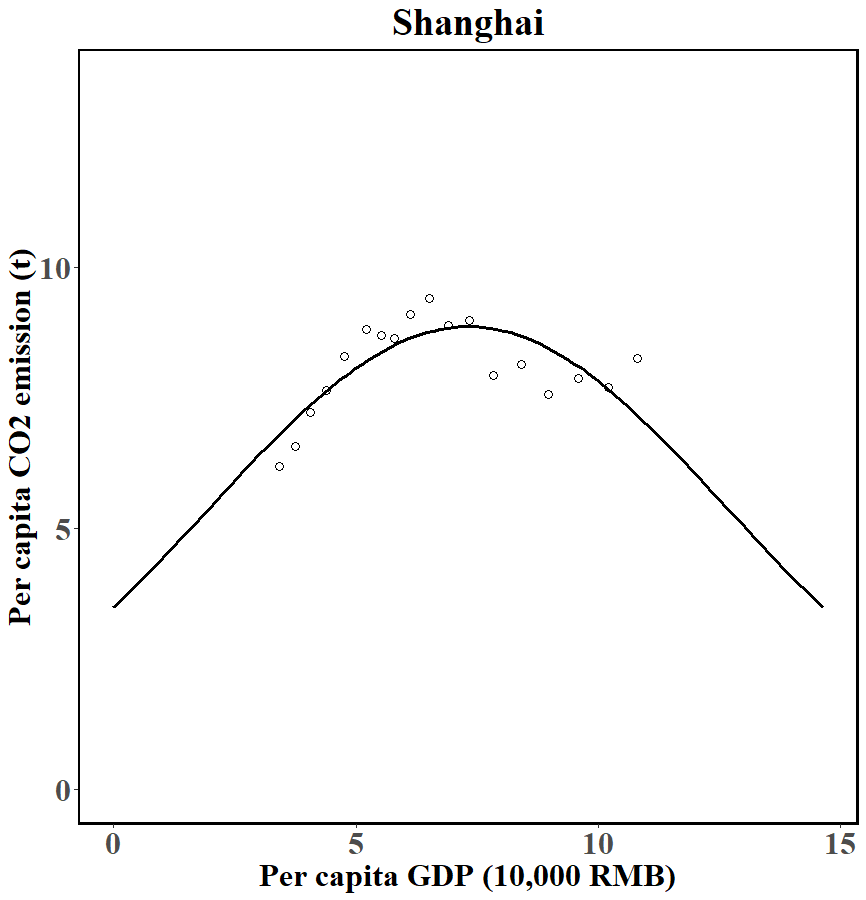

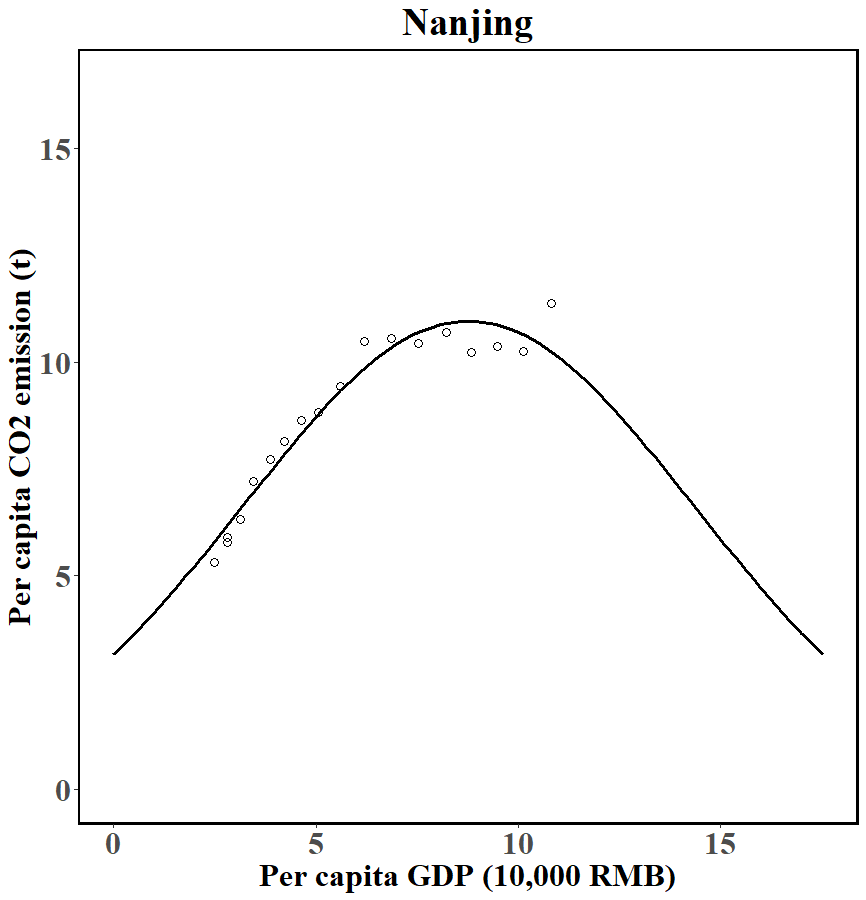

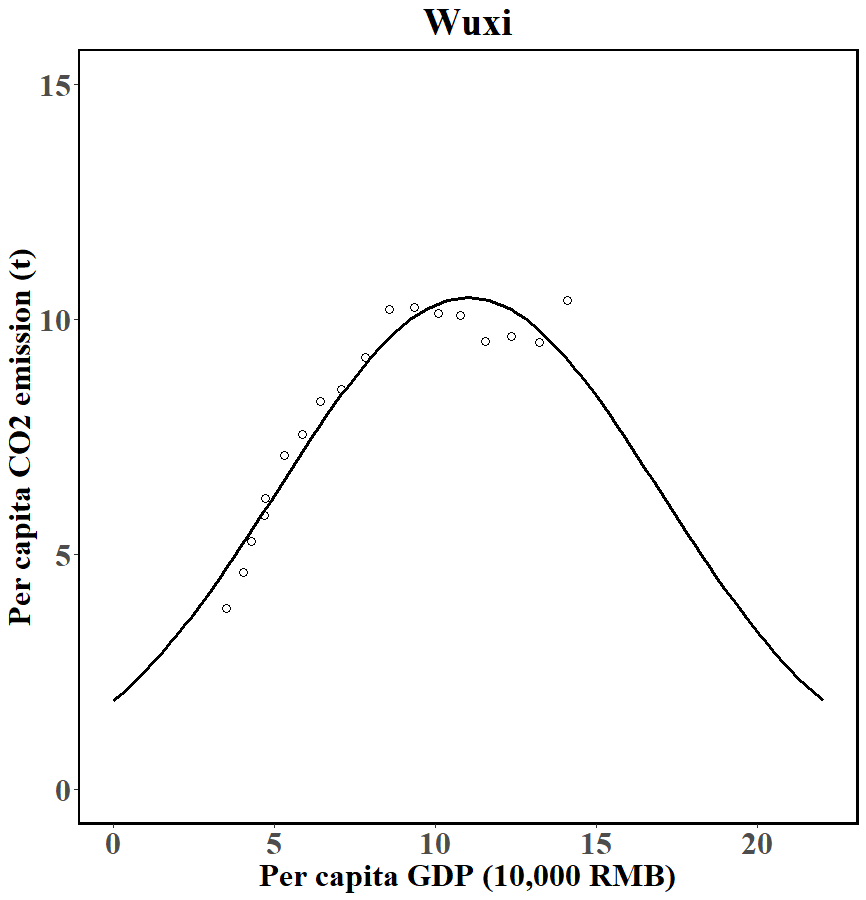

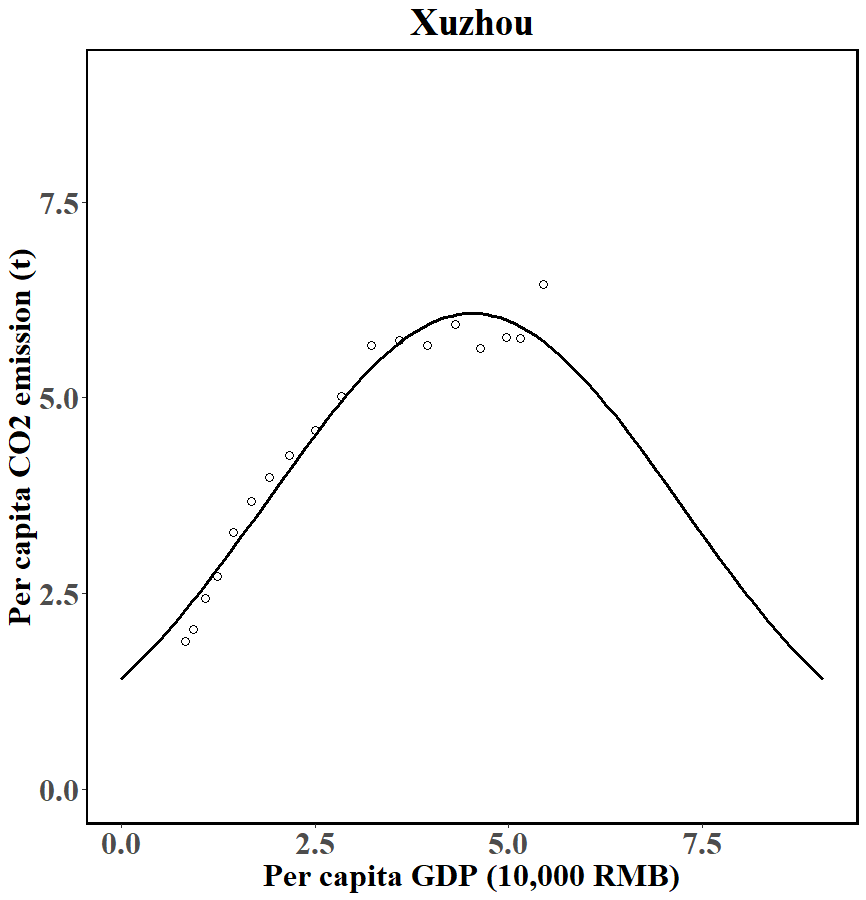

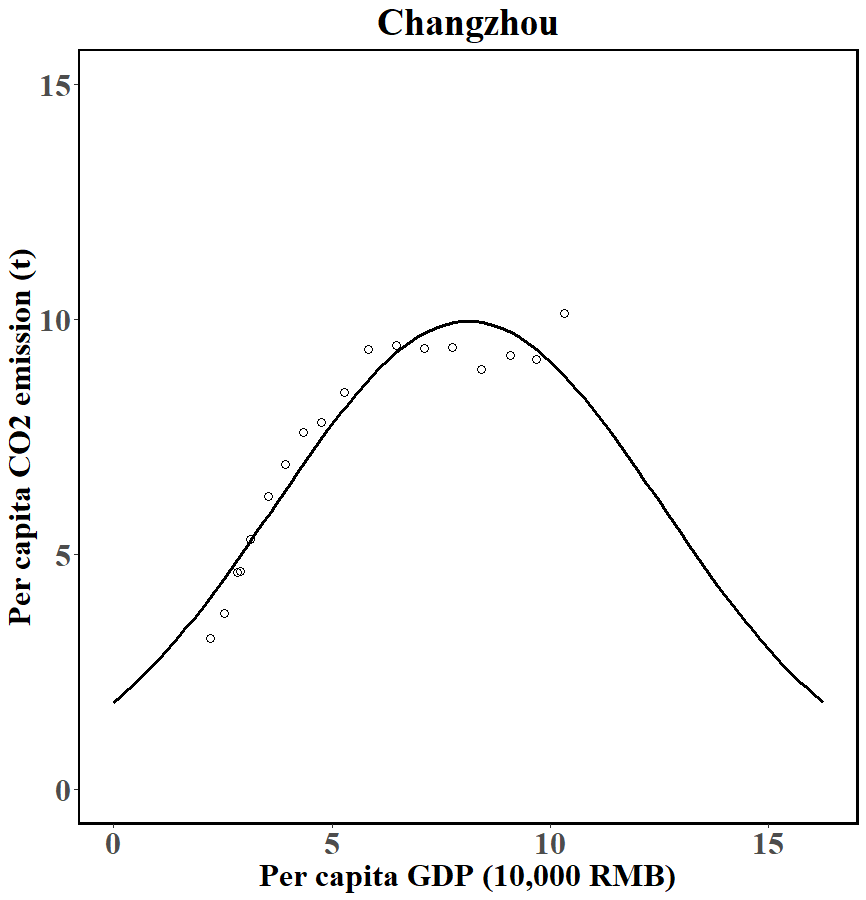

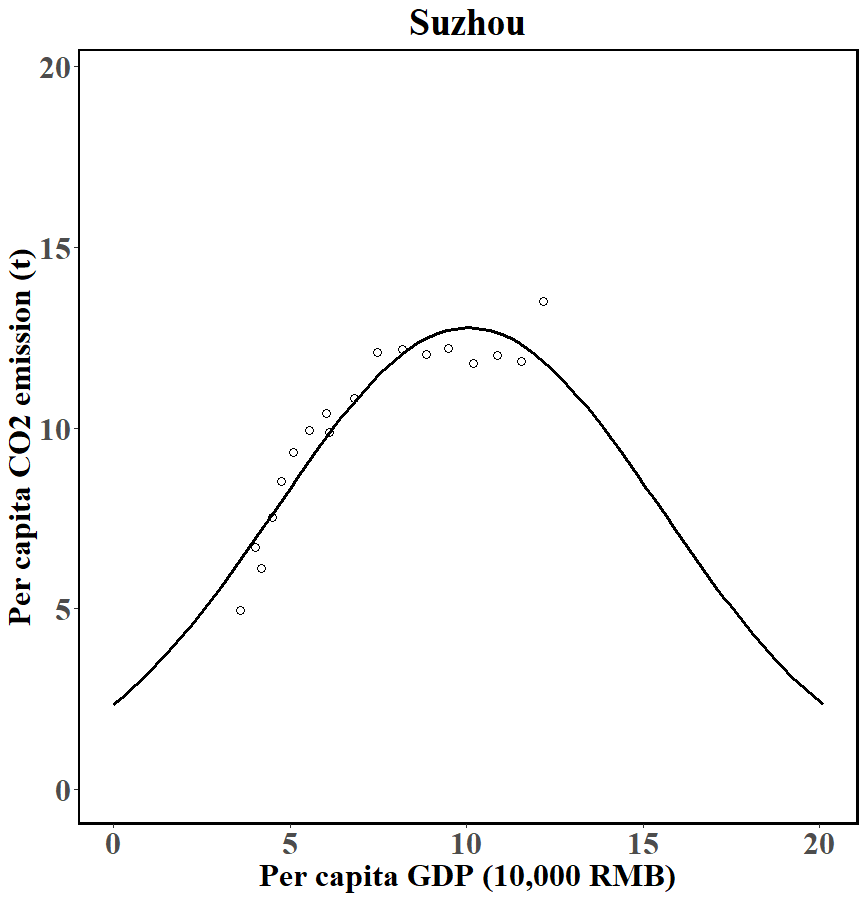

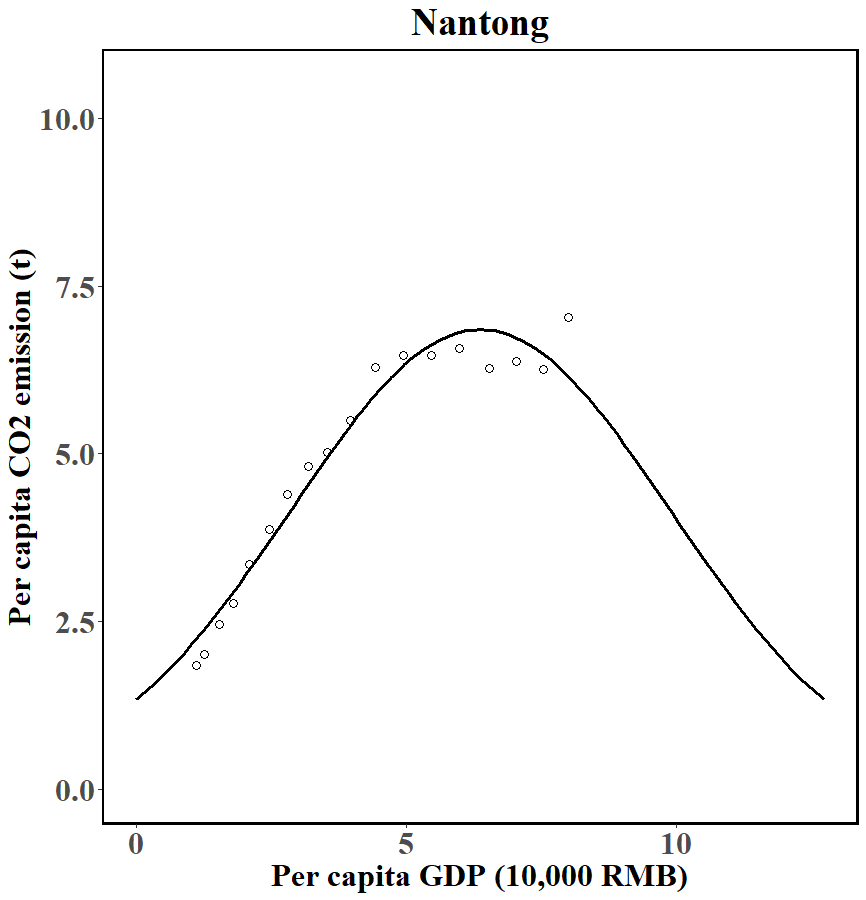

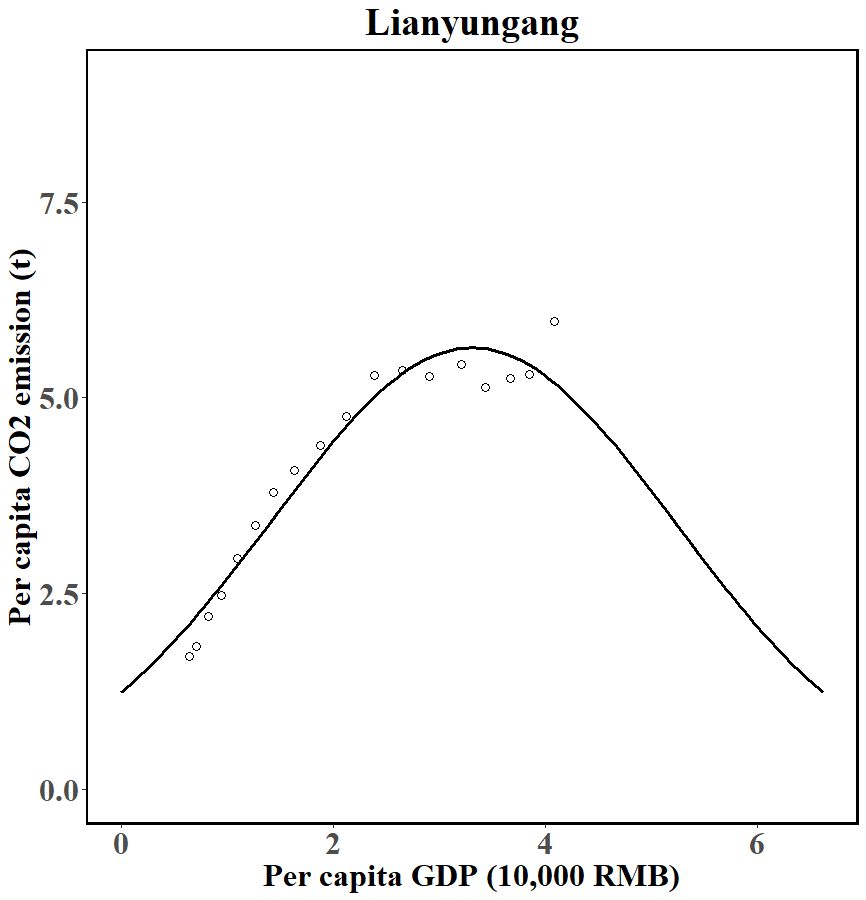

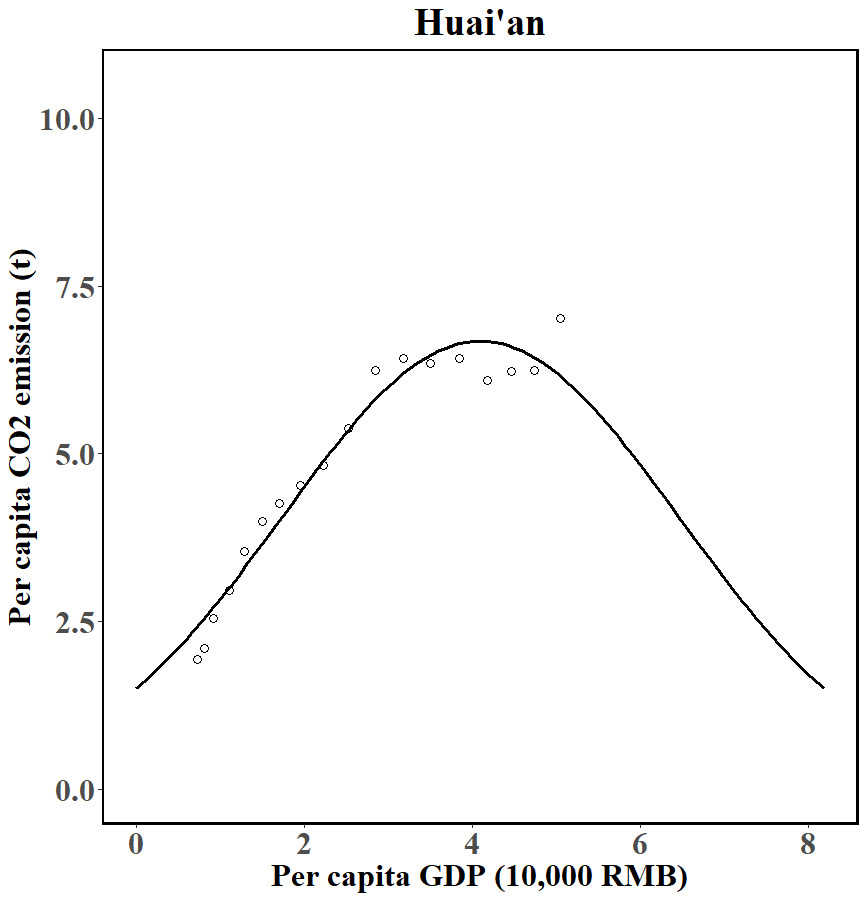

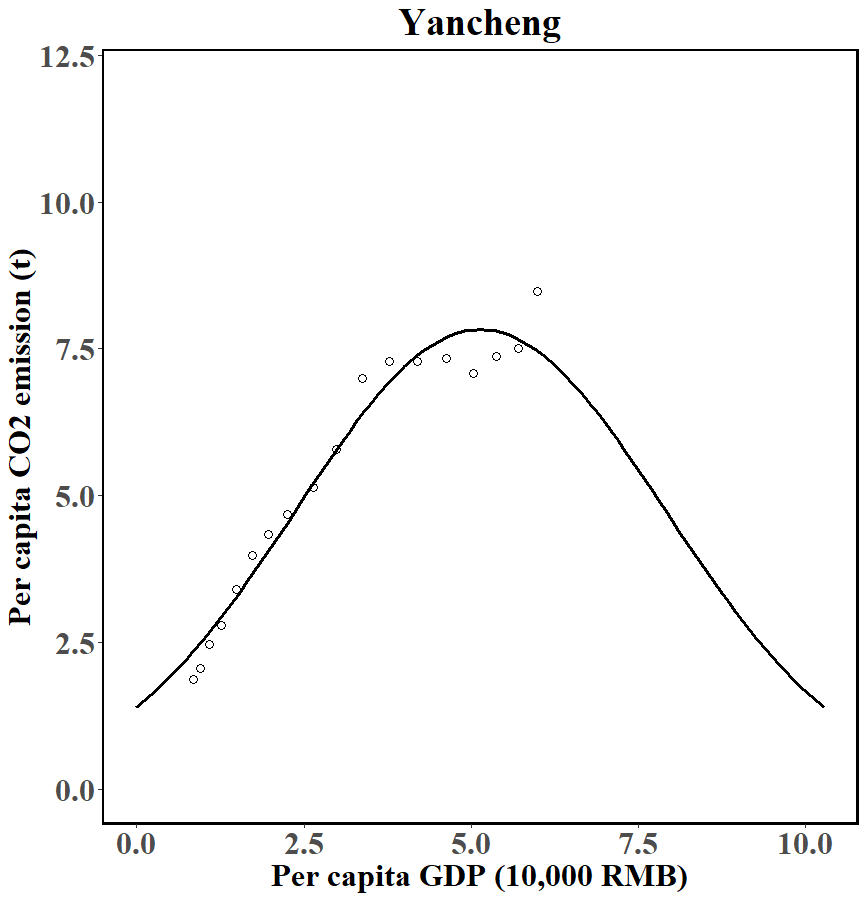

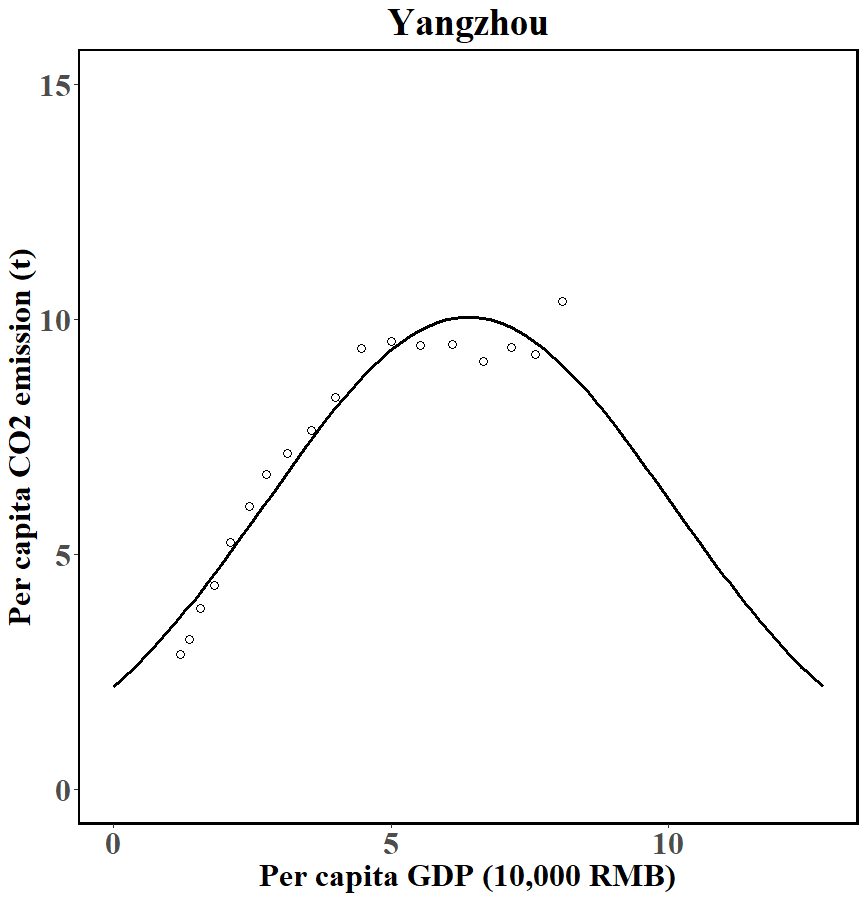

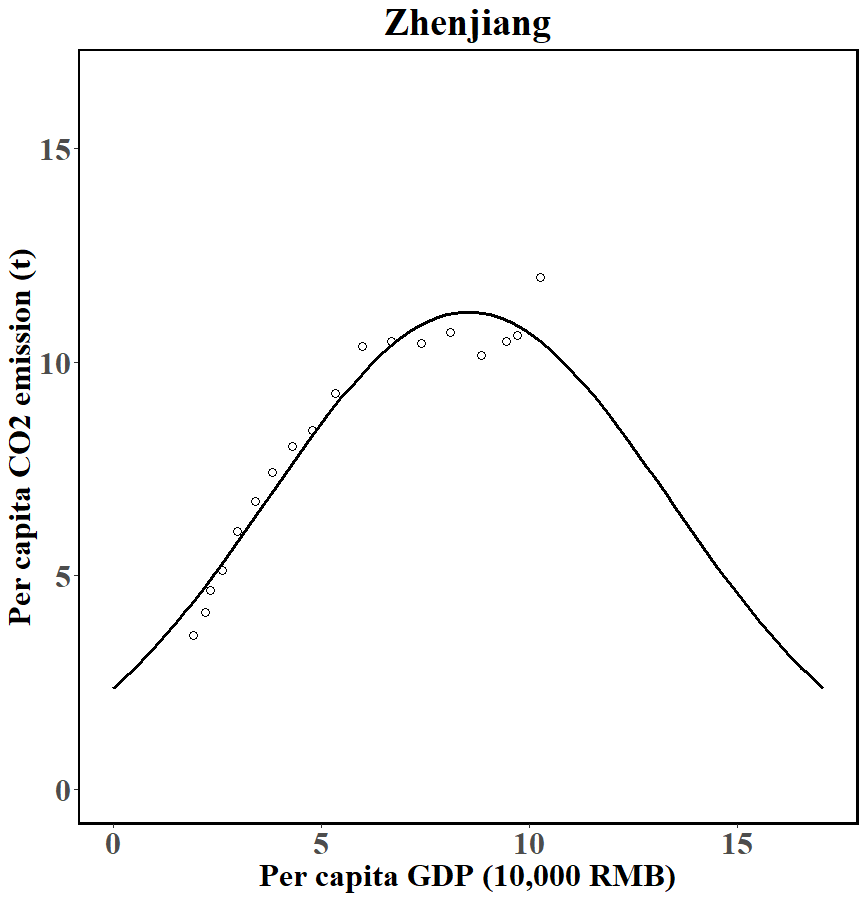

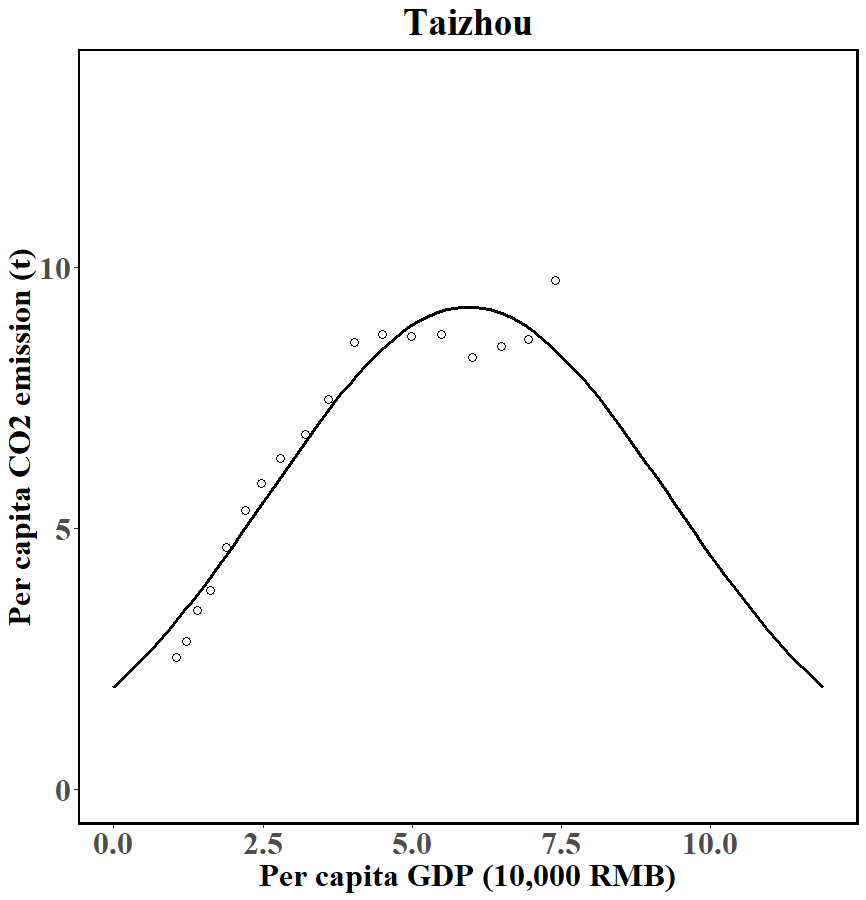

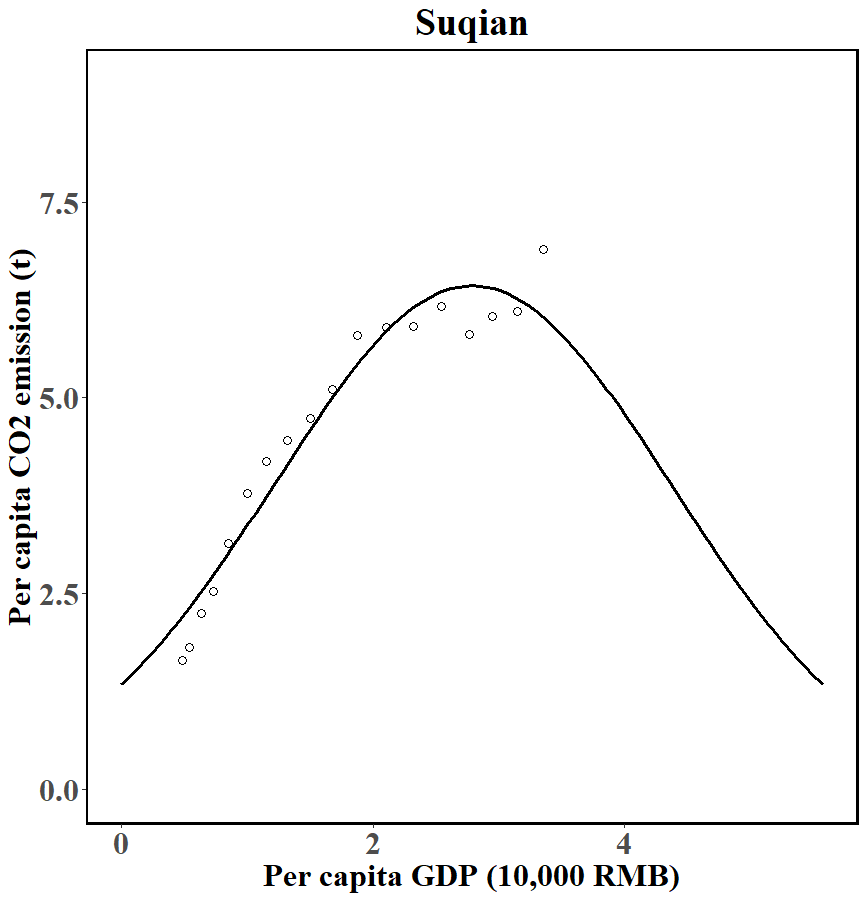

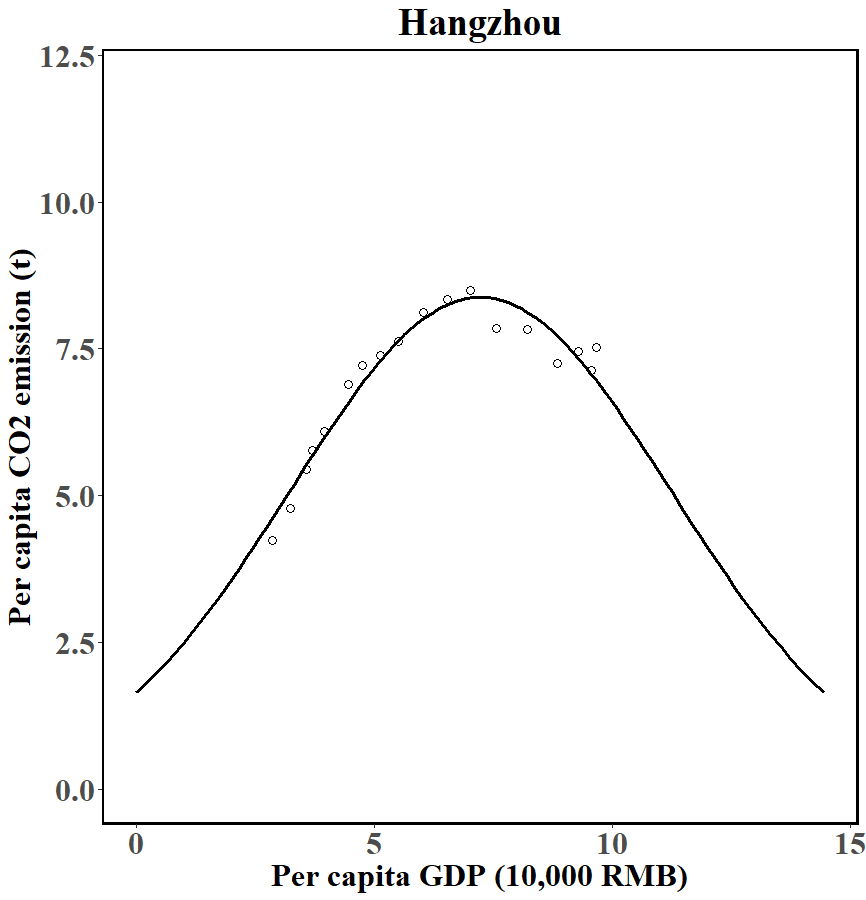

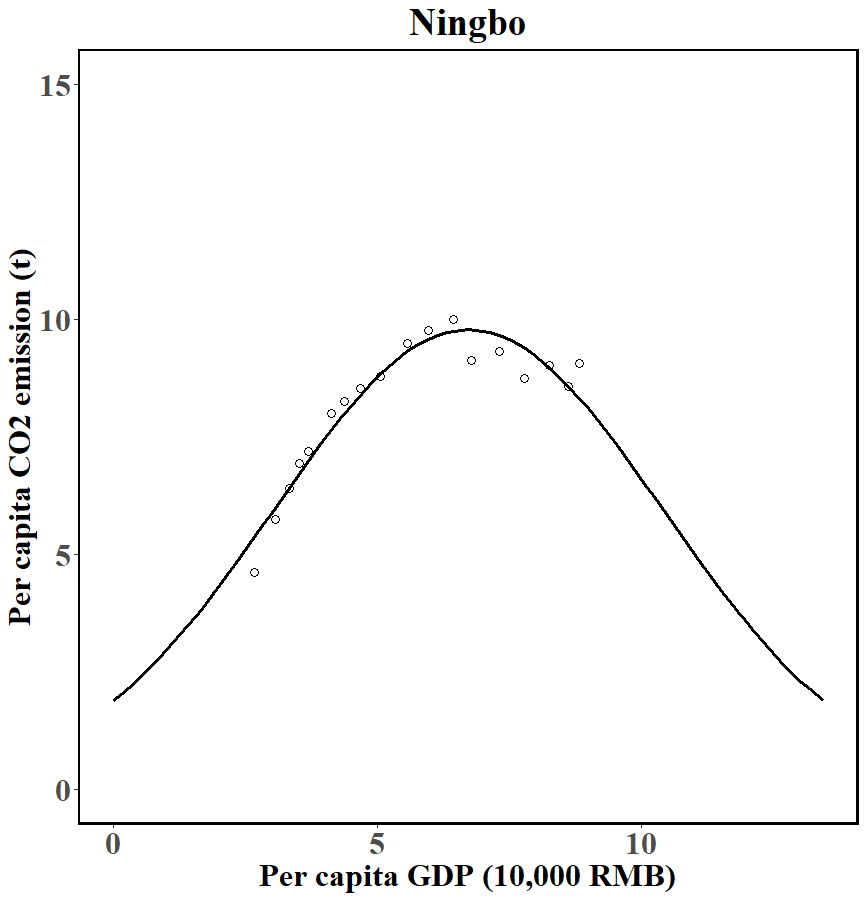

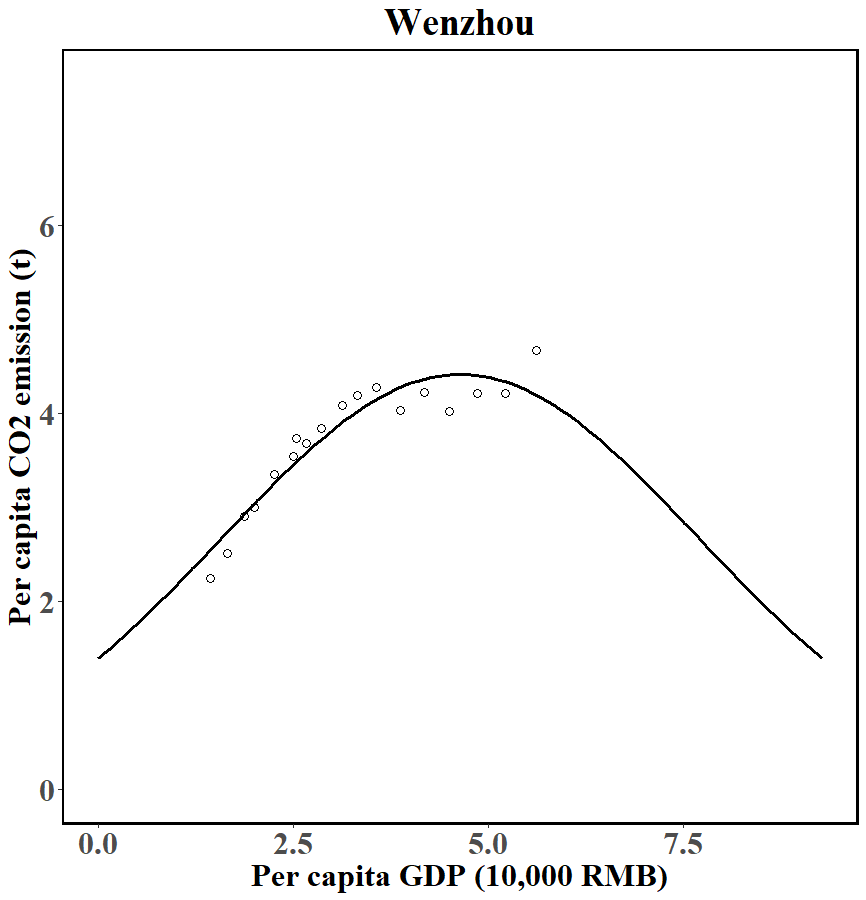

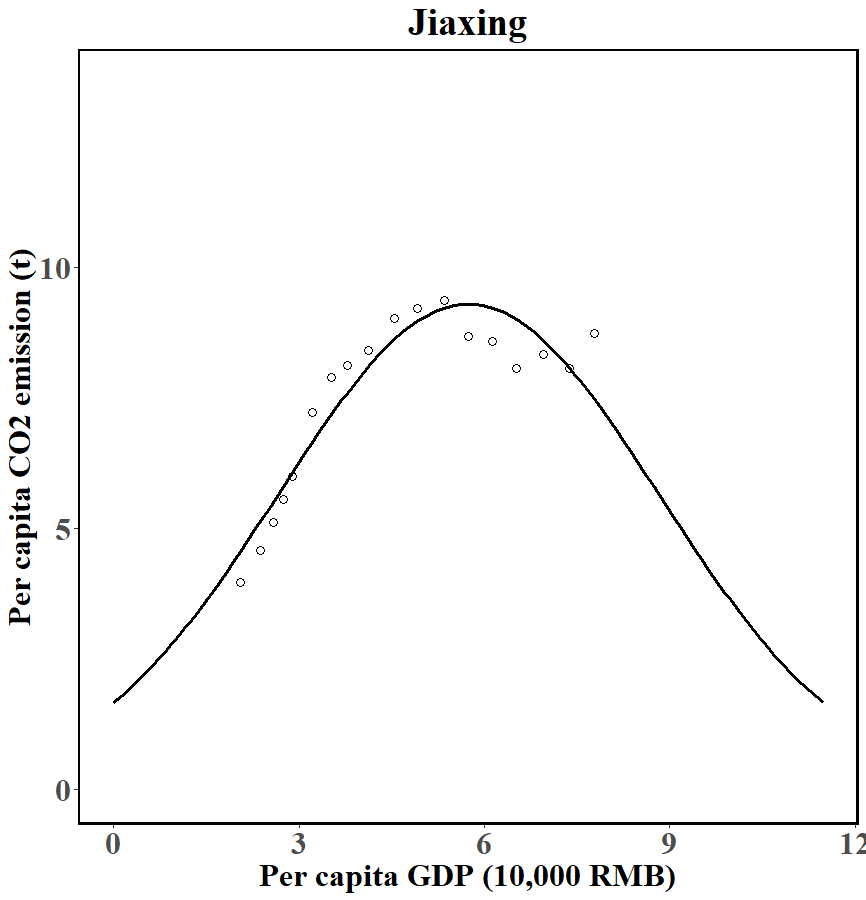

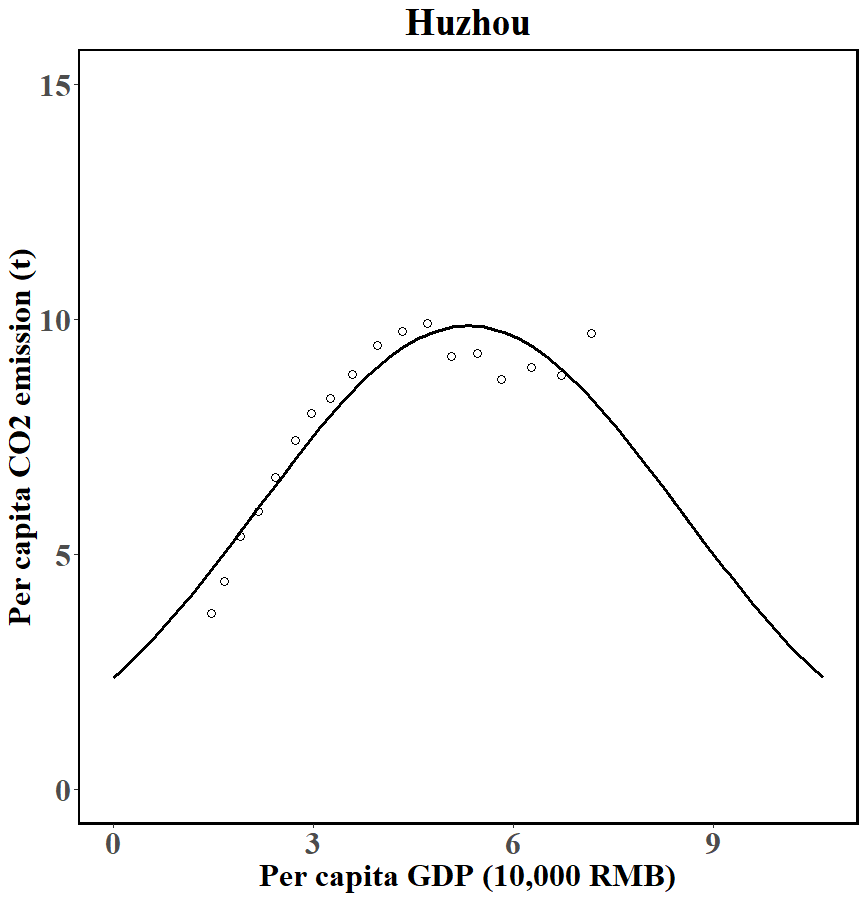


**Figure S3-2** **Gaussian Kuznets curve regression of per capita GDP and CO2 emission per capita for each of the 262 Chinese cities over 2002-2019. Note that the GDP per capita is based on the constant prices in 1997.**

**S4 Illustrations of category division of cities**

**Figure S4-1** **Illustrations of division of cities categorized by population size (a) and economic structure (b). Note that the division was based on the data in 2015.**

**S5 Sensitivity analysis**

**Downscaling CO_2_ emissions for China’s provinces, cities and counties**

As described in the main text, we found that the annual ratios of CO_2_ emissions at all levels to the national CO_2_ emission does not change significantly. We, therefore, assumed that the ratios in most areas at all levels would follow their changing trends in 2018 and 2019. Using Holt-Winters filter method, we forecasted CO_2_ emissions for 30 provinces, 292 cities and 2735 counties in mainland China in 2018 and 2019. Fig. S5-1 depicted the fitting effects between predicted ratios and actual ratios, indicating Holt-Winters filter method here can predict the changes in the ratios of CO_2_ emissions at all levels to the national CO_2_ emission accurately. The projected CO_2_ emissions for China’s provinces, cities and counties in 2018 and 2019 thus are reasonable.

**Figure S5-1** **Fitting effects of the predicted ratios and actual ratios for CO_2_ emission during 1999-2017 in China’s provinces (a), cities (b) and counties (c).**

**Scenario analysis for projecting China’s CO_2_ emission to 2035**

Following the analytic procedure in previous studies (Chen et al. 2020a; Ramírez et al. 2008), we used Monte Carlo simulation approach to test the sensitivity of the scenario analysis in the main text. Fig. S5-2 illustrated the results on China’s overall CO_2_ emission under different scenarios on carbon intensity and economic recovery by 100,000 times simulations in 2021. The median of simulated results is very close to the computed results in the scenario analysis in the main text, suggesting the projection China’s CO_2_ emission in 2021 in the main text is reliable. The definitions of scenarios were given in Table S5-1.

**Figure S5-2** **Monte Carlo simulation results of China’s CO_2_ emission under different scenarios in 2021.**

Further, Fig. S5-3 presented the simulated results of China’s overall CO_2_ emission under different scenarios on carbon intensity and economic recovery during 2021-2035. The simulated trajectories of CO_2_ emission are the similar to the results in Fig. 6 in the main text, and the overall difference are relatively small (Table S5-2), suggesting the scenario analysis on China’s CO_2_ emission in 2021-2035 is robust after considering the uncertainty using Monte Carlo simulation approach.

**Figure S5-3** **Monte Carlo simulation results of China’s CO_2_ emission under different scenarios in 2021-2035.**

**Table S5-1**

**Defininations of different scenarios in the study.**

| Scenario | Definination |
| --- | --- |
| A1 | BAUeconomic growth and BAU low-carbon technology with minimum speed in reducing carbon intensity |
| A2 | BAU economic growth and BAU low-carbon technology with medium speed in reducing carbon intensity |
| A3 | BAU economic growth and BAU low-carbon technology with maximum speed in reducing carbon intensity |
| A4 | Advanced economic growth and BAU low-carbon technology with minimum speed in reducing carbon intensity |
| A5 | Advanced economic growth and BAU low-carbon technology with medium speed in reducing carbon intensity |
| A6 | Advanced economic growth and BAU low-carbon technology with maximum speed in reducing carbon intensity |
| A7 | Moderate economic growth and BAU low-carbon technology with minimum speed in reducing carbon intensity |
| A8 | Moderate economic growth and BAU low-carbon technology with medium speed in reducing carbon intensity |
| A9 | Moderate economic growth and BAU low-carbon technology with maximum speed in reducing carbon intensity |
| A10 | BAU economic growth and moderate low-carbon technology with minimum speed in reducing carbon intensity |
| A11 | BAU economic growth and moderate low-carbon technology with medium speed in reducing carbon intensity |
| A12 | BAU economic growth and moderate low-carbon technology with maximum speed in reducing carbon intensity |
| A13 | Moderate economic growth and moderate low-carbon technology with minimum speed in reducing carbon intensity |
| A14 | Moderate economic growth and moderate low-carbon technology with medium speed in reducing carbon intensity |
| A15 | Moderate economic growth and moderate low-carbon technology with maximum speed in reducing carbon intensity |
| A16 | Advanced economic growth and moderate low-carbon technology with minimum speed in reducing carbon intensity |
| A17 | Advanced economic growth and moderate low-carbon technology with medium speed in reducing carbon intensity |
| A18 | Advanced economic growth and moderate low-carbon technology with maximum speed in reducing carbon intensity |
| A19 | BAU economic growth and advanced low-carbon technology with minimum speed in reducing carbon intensity |
| A20 | BAU economic growth and advanced low-carbon technology with medium speed in reducing carbon intensity |
| A21 | BAU economic growth and advanced low-carbon technology with maximum speed in reducing carbon intensity |
| A22 | Moderate economic growth and advanced low-carbon technology with minimum speed in reducing carbon intensity |
| A23 | Moderate economic growth and advanced low-carbon technology with medium speed in reducing carbon intensity |
| A24 | Moderate economic growth and advanced low-carbon technology with maximum speed in reducing carbon intensity |
| A25 | Advanced economic growth and advanced low-carbon technology with minimum speed in reducing carbon intensity |
| A26 | Advanced economic growth and advanced low-carbon technology with medium speed in reducing carbon intensity |
| A27 | Advanced economic growth and advanced low-carbon technology with maximum speed in reducing carbon intensity |

**Table S5-2**

**Mean absolute percentage errors between scenario results and simulated results using Monte Carlo simulation approach under different scenarios in 2021-2035.**

| Scenario | MAPE | Scenario | MAPE | Scenario | MAPE |
| --- | --- | --- | --- | --- | --- |
| A1 | 7.29 % | A2 | 0.05 % | A3 | 6.89 % |
| A4 | 7.32 % | A5 | 0.08 % | A6 | 6.86 % |
| A7 | 7.29 % | A8 | 0.05 % | A9 | 6.89 % |
| A10 | 6.85 % | A11 | 0.02 % | A12 | 6.45 % |
| A13 | 6.85 % | A14 | 0.02 % | A15 | 6.45 % |
| A16 | 6.85 % | A17 | 0.02 % | A18 | 6.45 % |
| A19 | 0.00 % | A20 | 0.00 % | A21 | 0.00 % |
| A22 | 0.00 % | A23 | 0.00 % | A24 | 0.00 % |
| A25 | 0.00 % | A26 | 0.00 % | A27 | 0.00 % |

**References**

1. Box, G. E., Jenkins, G. M., & Reinsel, G. C., 2011. Time series analysis: forecasting and control (Vol. 734). John Wiley & Sons.
2. Bu, Y., Wang, E., Bai, J., & Shi, Q. (2020). Spatial pattern and driving factors for interprovincial natural gas consumption in China: Based on SNA and LMDI. Journal of Cleaner Production, 263, 121392.
3. Chen, J., Xu, C., Shahbaz, M., & Song, M. (2020a). Interaction determinants and projections of China’s energy consumption: 1997–2030. Applied Energy, 116345.
4. Chen, J., Xu, C., & Song, M. (2020b). Determinants for decoupling economic growth from carbon dioxide emissions in China. Regional Environmental Change, 20(1), 11.
5. China Macroeconomy Forum (CMF). Retrieved 1 January 2021 from <http://ier.ruc.edu.cn/ltzj/hgjjjdbgfbh/jd2019n/2019_2020/index.htm> (in Chinese)
6. Conejo, A. J., Plazas, M. A., Espinola, R., & Molina, A. B. (2005). Day-ahead electricity price forecasting using the wavelet transform and ARIMA models. IEEE transactions on power systems, 20(2), 1035-1042.
7. Department of Ecology and Environment in Inner Mongolia. Retrieved 1 November 2020, from http://www.tanjiaoyi.com/article-31471-1.html (in Chinses).
8. Furht, B. (2010). Handbook of social network technologies and applications. Springer Science & Business Media.
9. Holt, C. C., 1957. Forecasting seasonals and trends by exponentially weighted moving averages. ONR Research Memorandum, Carnegie Institute of Technology.
10. Huang, G. B., Zhu, Q. Y., & Siew, C. K. (2006). Extreme learning machine: theory and applications. Neurocomputing, 70(1-3), 489-501.
11. International Monetary Fund (IMF). Retrieved 1 January 2021 from <https://www.imf.org/external/datamapper/datasets/WEO>
12. Institute for Advanced Research of Shanghai University of Finance and Economics (IAR-SUFE). Retrieved 1 January 2021 from <https://news.sufe.edu.cn/1c/f9/c186a138489/page.htm> (in Chinese)
13. Joo, T. W., & Kim, S. B. (2015). Time series forecasting based on wavelet filtering. Expert Systems with Applications, 42(8), 3868-3874.
14. Liu, Z., Ciais, P., Deng, Z., Lei, R., Davis, S. J., Feng, S.,..& Schellnhuber, H. J. (2020). Near-real-time monitoring of global CO_2_ emissions reveals the effects of the COVID-19 pandemic. Nature Communications, 11(1), 1–12.
15. McKibbin, W. J., & Fernando, R. (2020). The global macroeconomic impacts of COVID-19: Seven scenarios. Asian Economic Papers, 1-55.
16. Mi, Z., Wei, Y. M., Wang, B., Meng, J., Liu, Z., Shan, Y.,... & Guan, D. (2017). Socioeconomic impact assessment of China's CO2 emissions peak prior to 2030. Journal of Cleaner Production, 142, 2227–2236.
17. Mitra, S., & Pal, S. K. (1995). Fuzzy multi-layer perceptron, inferencing and rule generation. IEEE Transactions on Neural Networks, 6(1), 51-63.
18. Ord, K., Fildes, R. A., & Kourentzes, N. (2017). Principles of business forecasting. Wessex Press Publishing Co.
19. Ramírez, A., de Keizer, C., Van der Sluijs, J. P., Olivier, J., & Brandes, L. (2008). Monte Carlo analysis of uncertainties in the Netherlands greenhouse gas emission inventory for 1990–2004. Atmospheric Environment, *42*(35), 8263-8272.
20. Salles, R., Belloze, K., Porto, F., Gonzalez, P. H., & Ogasawara, E. (2019). Nonstationary time series transformation methods: An experimental review. Knowledge-Based Systems, 164, 274-291.
21. Scott, J. (1988). Social network analysis. Sociology, *22*(1), 109-127.
22. Specht, D. F. (1991). A general regression neural network. IEEE transactions on neural networks, *2*(6), 568-576.
23. Stolojescu, C., Railean, I., Moga, S., Lenca, P., & Isar, A. (2010, June). A wavelet based prediction method for time series. In Proceedings of Stochastic Modeling Techniques and Data Analysis (SMTDA2010) International Conference, Chania, Greece (pp. 767-774).
24. Winters, P. R. (1960). Forecasting sales by exponentially weighted moving averages. Management science, 6(3), 324-342.
25. World Bank. Retrieved 1 October 2020 from https://www.worldbank.org/en/publication/global-economic-prospects
26. Zhang, X., Geng, Y., Shao, S., Dong, H., Wu, R., Yao, T., & Song, J. (2020). How to achieve China’s CO2 emission reduction targets by provincial efforts? –An analysis based on generalized Divisia index and dynamic scenario simulation. Renewable and Sustainable Energy Reviews, 127, 109892.
